# Cryo-EM structure of GABA transporter 1 reveals substrate recognition and transport mechanism

**DOI:** 10.1038/s41594-023-01011-w

**Published:** 2023-07-03

**Authors:** Smruti Ranjan Nayak, Deepthi Joseph, Georg Höfner, Archishman Dakua, Arunabh Athreya, Klaus T. Wanner, Baruch I. Kanner, Aravind Penmatsa

**Affiliations:** 1 Molecular Biophysics Unit, Indian Institute of Science, Bangalore, India; 2 Department of Pharmacy, Center for Drug Research, Ludwig Maximilians University of Munich, Munich, Germany; 3 Department of Biochemistry and Molecular Biology, Institute for Medical Research Israel-Canada, Hebrew University, Hadassah Medical School, Jerusalem, Israel; 4 Present Address: Department of Molecular Biosciences, College of Natural Sciences, University of Texas at Austin, Austin, TX USA; 5 Present Address: Biophysics Program, University of Wisconsin–Madison, Madison, WI USA

**Keywords:** Cryoelectron microscopy, Membrane proteins

## Abstract

The inhibitory neurotransmitter γ-aminobutyric acid (GABA) is cleared from the synaptic cleft by the sodium- and chloride-coupled GABA transporter GAT1. Inhibition of GAT1 prolongs the GABAergic signaling at the synapse and is a strategy to treat certain forms of epilepsy. In this study, we present the cryo-electron microscopy structure of *Rattus norvegicus* GABA transporter 1 (rGAT1) at a resolution of 3.1 Å. The structure elucidation was facilitated by epitope transfer of a fragment-antigen binding (Fab) interaction site from the *Drosophila* dopamine transporter (dDAT) to rGAT1. The structure reveals rGAT1 in a cytosol-facing conformation, with a linear density in the primary binding site that accommodates a molecule of GABA, a displaced ion density proximal to Na site 1 and a bound chloride ion. A unique insertion in TM10 aids the formation of a compact, closed extracellular gate. Besides yielding mechanistic insights into ion and substrate recognition, our study will enable the rational design of specific antiepileptics.

## Abstract (web summary)

The GABA transporters (GATs) enforce spatiotemporal control of the inhibitory neurotransmitter GABA at neural synapses. Nayak et al. report the structure of substrate-bound GAT1 in an ion-bound cytosol-facing conformation, providing mechanistic insights into GABA selection and reuptake.

## Main

In the synaptic space, levels of the major inhibitory neurotransmitter, GABA, are mediated by the GABA transporter (GAT) isoforms that belong to the neurotransmitter sodium symporters (NSSs). GABA, discovered in brain lysates^1^, is vital for neuronal synchronization in the cortical neurons and maintains a balance between excitatory and inhibitory neurotransmission at postsynaptic neurons^2^. Altered GABA levels shift this balance and can lead to multiple pathologies such as seizures, anxiety and schizophrenia^3,4^. The uptake of released GABA through GAT isoforms GAT1, GAT2 and GAT3 and the betaine/GABA transporter (BGT1) uses Na^+^ and Cl^-^ ions for the symport of GABA into the neurons and glial cells^5^. GABA transport was originally identified in studies on brain slices^6^.

The GAT isoforms are responsible for the spatiotemporal control of GABA levels in the neural synapses^7^ and constitute an attractive target for enhancing synaptic GABA levels. The GATs belong to the solute carrier 6 (SLC6) family of NSSs, and rGAT1 was the first member from this family to be cloned^8^. GAT1 is also the major neuronal isoform and is the target of the antiepileptic drug tiagabine, which is prescribed for partial seizures^9^. The transporter topology resembles the structure of the amino acid transporter LeuT^10^ and other well-studied SLC6 members such as dDAT^11^, the human serotonin transporter (hSERT)^12^ and the human glycine transporter (hGlyT1)^13^ with 12 transmembrane helices. The topology comprises a set of scaffold helices and a bundle consisting of two symmetric but discontinuous helices, transmembrane helix 1 (TM1) and TM6, and TM2 and TM7. The bundle moves relative to the scaffold to open and close the extracellular pathway. The cytoplasmic pathway opens by the additional movement of TM1a, which pivots from the center of the transporter to swing away from the rest of the bundle^14,15^. NSS members shift through several conformations of outward-open, occluded and inward-open states to move neurotransmitters into neurons and glial cells, against their concentration gradient^15–17^.

In SLC6 members, competitive inhibitors interact at the substrate-binding site^12,18,19^, and non-competitive inhibitors have interaction sites at the extracellular vestibule or the cytosolic half of the transporter as observed in hSERT and hGlyT1 (Refs. ^12,13,17^). The recent cryo-electron microscopy (cryo-EM) structure of human GAT1 at 3.8 Å reveals a cytosol-facing state with tiagabine interacting with the transporter ‘beneath’ (towards the cytosol) the substrate-binding site^20^. The X-ray structures of an engineered dDAT with a GAT1-like binding site reveal interactions of the tiagabine analogues, NO711 and SKF89976a, bound in a bean-shaped primary binding site^21^. An additional molecule of SKF89976a binds to the extracellular vestibule of the transporter, resembling interactions of citalopram and vilazodone in hSERT^12,21,22^. These observations reveal previously unexplored diversity in the context of GAT1 inhibition.

Here, we present the cryo-EM structure of rGAT1 at a resolution of 3.1 Å in a cytosol-facing conformation with densities for substrate, sodium (near Na site 1) and chloride ions. The rGAT1 structure reveals a closed extracellular gate facilitated by the interactions between residues from the bundle of gating helices and the scaffold helices with a solvent-accessible substrate-binding site facing the cytosol. The modified rGAT1 retains GABA uptake activity and displays direct high-affinity interaction with a GAT1 inhibitor, NO711, in cell membranes. Comparisons of the substrate-bound rGAT1 with an AlphaFold2 model of GAT1 in the outward-open conformation reveal valuable insights into substrate recognition and the transport mechanism involved in GABA reuptake and its inhibition.

## Results

### Epitope transfer facilitates rGAT1 structure determination

**Fig. 1:**
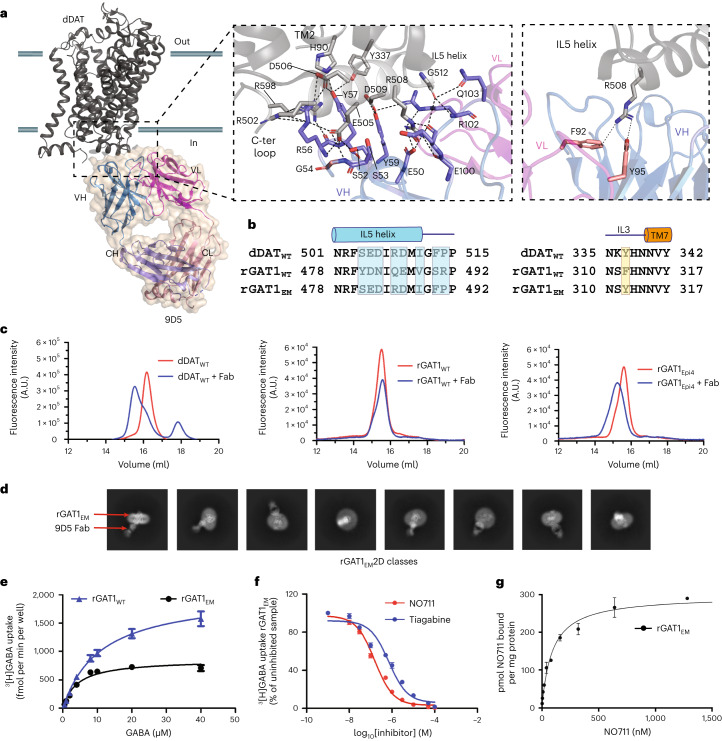
Epitope transfer from dDAT facilitates GAT1 reconstruction. **a**, X-ray structure of dDAT interacting with the Fab, 9D5 (PDB 4XP1), at the cytosolic face with heavy (blue) and light (pink) chain epitope interactions displayed as inset images. Interactions are given as dashed lines. **b**, Sequence alignment of the epitope region around IL3 and IL5 in dDAT compared with rGAT1_WT_. The epitope substitutions made in rGAT1_WT_ to prepare the engineered construct, rGAT1_EM_, are highlighted in colored boxes. **c**, FSEC results displaying shifts in the GFP fluorescence peak elution volume in the complexed and uncomplexed forms of rGAT1_WT_ and rGAT1_Epi4_ to highlight the presence of 9D5 interactions in the engineered rGAT1 construct. A.U., arbitrary units. **d**, 2D classes show the presence of Fab interactions with rGAT1_EM_ in multiple orientations. **e**, ^3^H-GABA uptake assays were performed in two independent measurements (*n* = 2), each carried out in triplicate. Each point represents a mean of six measurements, and error bars represent s.e.m. The two independent datasets are displayed in Supplementary Figs. 4 and 5. The *K*_m_ and *V*_max_ values are 11 μM and 2,023 fmol per well per min for rGAT1_WT_ and 4.2 μM and 855.7 fmol per well per min for rGAT1_EM_. **f**, Measurement of tiagabine and NO711 inhibition potency against rGAT1_EM_ indicates an IC_50_ of 704 nM and 154.9 nM, respectively. Uptake measurements were performed as two independent experiments (*n* = 2), each carried out in triplicate. Each data point in the graph represents an average of all six measurements with error bars representing s.e.m. **g**, Binding measurements of rGAT1_EM_ expressing membranes with NO711, a tiagabine analogue. The affinity (*K*_d_) displayed by the interaction is 80 nM in comparison to 50 nM for rGAT1_WT_. Data points in the binding curve represent specific binding (means ± s.d.) calculated from total and non-specific binding measured by LC–MS shown in Supplementary Figs. 5–6 of one trial of four (*n* = 4) (rGAT1_EM_) independent experiments, each performed in triplicate, with error bars representing s.d. Source data

**Extended Data Fig. 1:**
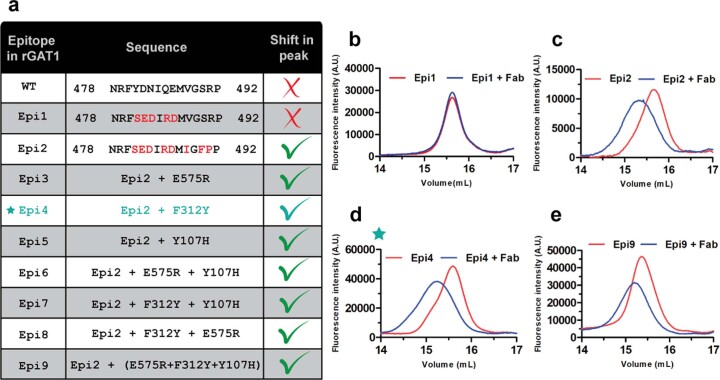
Epitope engineered constructs screened using FSEC. **a**, Table of epitope mutants sequentially engineered into rGAT1_WT_ and the list of residues altered for each construct. Presence of a shift in peak profile upon incubating with 9D5 indicated with checks and crosses, suggest their ability to bind to the Fab, 9D5. **b-e**, FSEC profiles of a few epitope mutants with and without incubating with the Fab. rGAT1_WT_ and rGAT1_Epi1_ constructs did not display any 9D5 binding (shift in peak profile) whereas other epitope mutants could bind to 9D5. The rGAT1_Epi4_ mutant exhibited the maximum peak shift among all epitope mutants (highlighted with green star). Source data

Cryo-EM structure determination of small integral membrane proteins requires the use of chaperones that enhance the size of the complex and facilitate particle alignment during image processing^23^. We generated a chaperone against GAT1 through the use of epitope transfer from dDAT to rGAT1 by substituting equivalent residues in intracellular loop 3 (IL3) and IL5 of rGAT1 to resemble dDAT (Fig. 1a,b, Extended Data Fig. 1 and Supplementary Fig. 1). This allowed the Fab, 9D5, previously used to crystallize dDAT, to interact with rGAT1 for cryo-EM structure elucidation. A series of mutant constructs (Epi) were designed that include multiple substitutions in the IL5 of rGAT1 and a single residue substitution in IL3 (Extended Data Fig. 1 and Supplementary Fig. 1). The region is the primary site of dDAT–9D5 interactions, particularly with the complementarity determining regions of the heavy chain in the Fab (Fig. 1a,b). The heavy chain has a maximal interfacial area of 545 Å^2^, and the light chain displays minimal interactions, with a surface area of 67 Å^2^. These substitutions yielded a construct of rGAT1 (Epi4) that displays maximal shifts in comparison to other engineered epitope constructs and interacts with the Fab when observed in the 2D classes (Fig. 1c,d). We used the Epi4 mutant combination, along with an amino-terminal deletion of 37 residues (Δ2–38) that are predicted to be disordered in an rGAT1 AlphaFold2 model, as the primary construct in this study, referred to as rGAT1_EM_.

**Extended Data Fig. 2:**
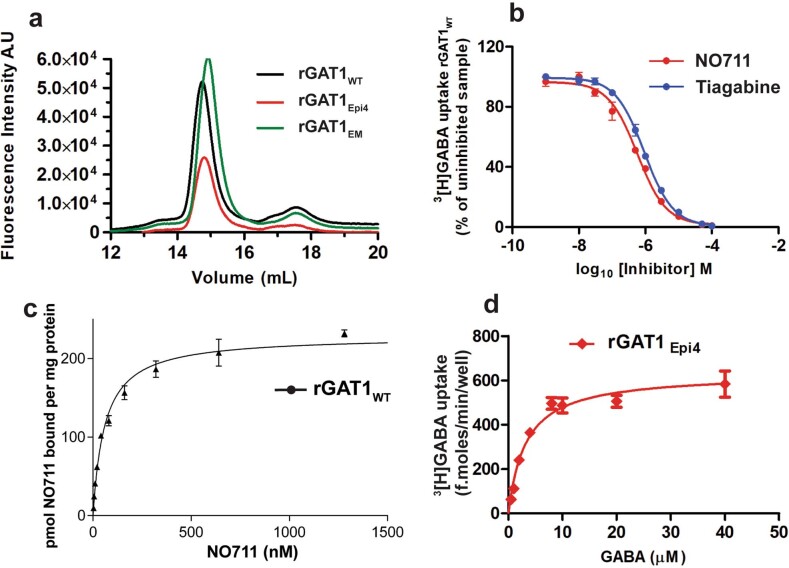
^3^[H]-GABA uptake and Inhibitor binding assays. **a**, Expression profiles of rGAT1_WT_, rGAT1_Epi4_ and rGAT1_EM_ observed using FSEC. **b**, Inhibition of ^3^H-GABA uptake through rGAT1_WT_ by tiagabine and NO711 displays IC_50_ values of 918 nM and 563 nM, respectively. Each data point in the graph represents an average of six measurements obtained from two independent sets (n = 2) each done in triplicate with error bars representing SEM (Supplementary Table 1, Supplementary Fig. 3). **c**, MS-based binding measurements of rGAT1_WT_ expressing membranes with inhibitor NO711 displaying a *K*_d_ value of 50 nM. Data points represent specific binding (means ± s.d) calculated from total and non-specific binding measured by LC-MS shown in Supplementary Figs. 4 & 7 of one of three independent experiments (n = 3), each performed in triplicate. Error bars in binding curve represent s.d. **d**, ^3^H-GABA uptake of rGAT1_Epi4_ mutant displaying *K*_M_ of 3.2 μM (Supplementary Table 1). Each data point in the uptake curve is an average of six measurements obtained from two independent sets (n = 2) each done in triplicate (Supplementary Fig. 5). The error bars represent SEM. Source data

**Extended Data Fig. 3:**
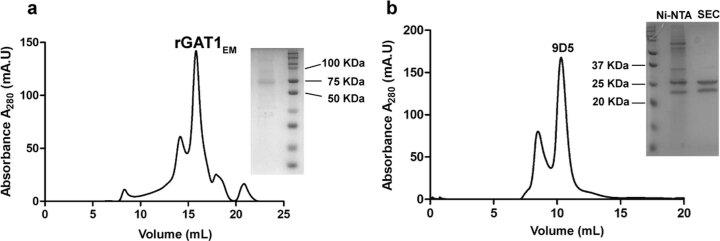
Purification of rGAT1_EM_ and Fab-9D5. **a**, SEC profile of rGAT1_EM_ in a Superose 6 Increase 10/300 GL column (Cytiva) and SDS PAGE analysis of the peak fraction. **b**, SEC profile of 9D5 with GFP in a Superdex 75 Increase 10/300 GL column and SDS PAGE analysis of the peak fraction. One SEC profile and one SDS-PAGE gel is being shown for the heterologous expression and purification of rGAT1_EM_ and Fab 9D5 used to prepare the rGAT1_EM_-9D5 complex grids in this study. Source data

**Extended Data Fig. 4:**
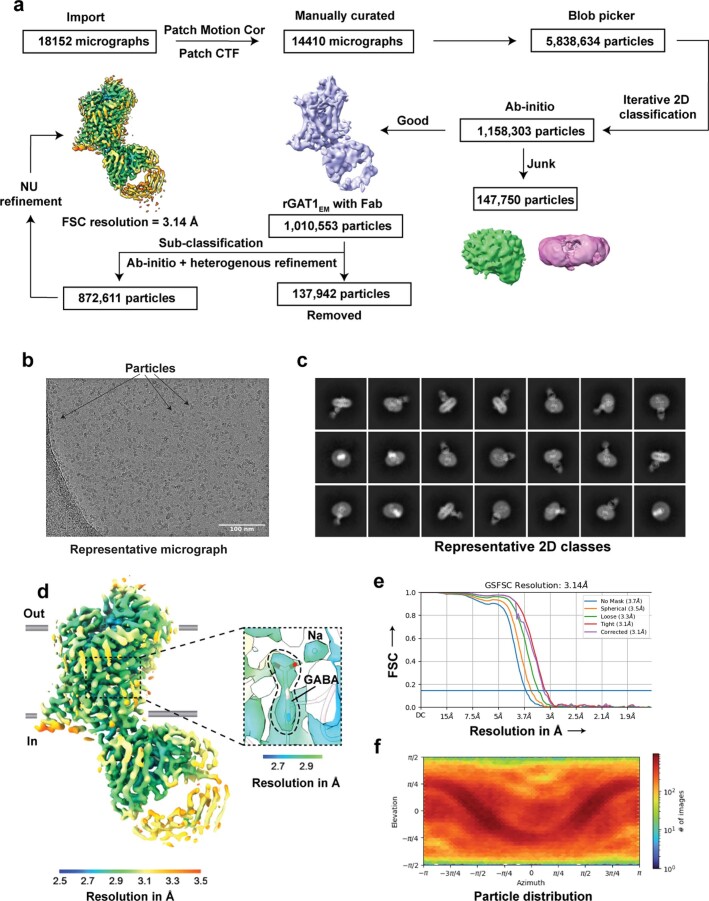
Structure determination using cryo-EM. **a**, Workflow of cryo-EM data processing of rGAT1_EM_ in complex with an antibody 9D5-Fab, in the inward-open conformation. The entire data processing was done in CryoSPARC^48^. After motion-correction and CTF estimation, exposures were curated to remove poor micrographs. Particles were picked and sorted using 2D classifications followed by ab-initio reconstruction of 3D model. The particles from good ab-initio classes were merged and further 3D classified and refined through heterogeneous refinement. A final set of particles (872,611) was refined using non-uniform refinement yielding a map of 3.1 Å resolution. **b**, Representative cryo-EM micrograph of rGAT1_EM_-Fab complex particles. Scale bar represents 100 nm. **c**, Representative 2D classes showing different orientation of micelle surrounded rGAT1_EM_ bound to 9D5-Fab. **d**, Cryo-EM density map colored by local resolution ranging from 2.5 Å to 3.5 Å. A bulk of the map has a resolution better that 3.0 Å. Insets show the binding pocket with GABA and ion densities colored as per local resolution in both top and bottom views reflecting a resolution in the range of 2.7–2.9 Å **e**, Gold standard FSC curves for different masking routines during refinement of rGAT1. **f**, Angular distribution of particles displays a near complete coverage of all possible orientations of rGAT1 without orientation bias.

The rGAT1_EM_ is a functional construct catalyzing^3^H-GABA uptake with a lower Michaelis constant, *K*_m_ (4.2 μM), compared to wild-type rGAT1 (rGAT1_WT_), which has a *K*_m_ value of 11 μM and a lowered *V*_max_ (Fig. 1e, Supplementary Table 1 and Extended Data Fig. 2) despite having expression levels similar to rGAT1_WT_ (Extended Data Fig. 2a and Supplementary Table 1). rGAT1_EM_ is also inhibited by tiagabine and NO711, with inhibition potencies (K_0.5_) of 700 nM and 155 nM, respectively, similar to rGAT1_WT_ (Fig. 1f, Extended Data Fig. 2b and Supplementary Table 1). Liquid chromatography-electrospray ionization coupled with tandem mass spectrometry (LC-ESI–MS/MS)-based inhibitor binding assays with membranes containing rGAT1 constructs yielded dissociation constants (*K*_d_) of 50 nM for rGAT1_WT_ and 80 nM for rGAT1_EM_ for the inhibitor, NO711, which are consistent with values for mouse and human GAT1 (Fig. 1g, Extended Data Fig. 2c and Supplementary Table 1c)^24^. The purified rGAT1_EM_ remains bound to 9D5 on the cryo-EM grid, as seen in 2D classes for which no orientation bias was observed during cryo-EM structure determination (Extended Data Figs. 3 and 4).

### rGAT1 structure displays a cytosol-facing conformation

**Fig. 2:**
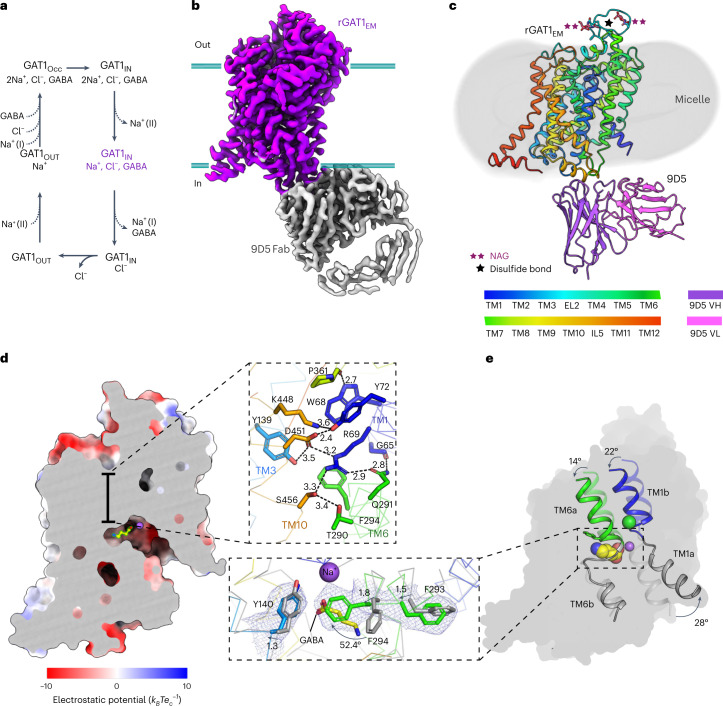
Cryo-EM structure of rGAT1_EM_ in the cytosol-facing state. **a**, Transport intermediates of GAT1 displaying cyclical inward- to outward-open states of the rGAT1. The GAT1 bound to GABA, a sodium at site 1 and a chloride ion is the primary conformation observed in the study (in color). **b**, Cryo-EM map of the refined rGAT1_EM_ structure (deep purple) bound to 9D5 (grey). The VH and VL part is ordered, although the constant domains lack clear density due to inherent disorder. **c**, The 12 transmembrane helix structure of rGAT1 in the detergent micelle (grey) displaying the transmembrane helices colored in spectrum. The VH (purple) and VL (pink) domains of the Fab were modeled and the constant domains were not modeled due to incomplete densities. *N*-glycosylation sites (NAG) and the disulfide bond are indicated in the extracellular loop 2. **d**, Electrostatic surface cutaway of rGAT1 displays substrate cavity exposed to solvent and the substrate GABA. The inset shows the thick extracellular gate and the hydrogen bond network of the residues that comprise the closed gate. Interaction distances are represented as dashed lines with values representing angstroms. **e**, The movement of TM1b and TM6a by 22° and 14°, respectively, obtained through structural comparison of an rGAT1 AlphaFold2 model in the outward-open state, allows closure of the extracellular gate. The inset shows the movement of Phe294 in the TM6 non-helical region that controls solvent access to the binding pocket and the side chain densities of Tyr140, Phe294 and Phe293 at σ level of 6.0.

**Extended Data Fig. 5:**
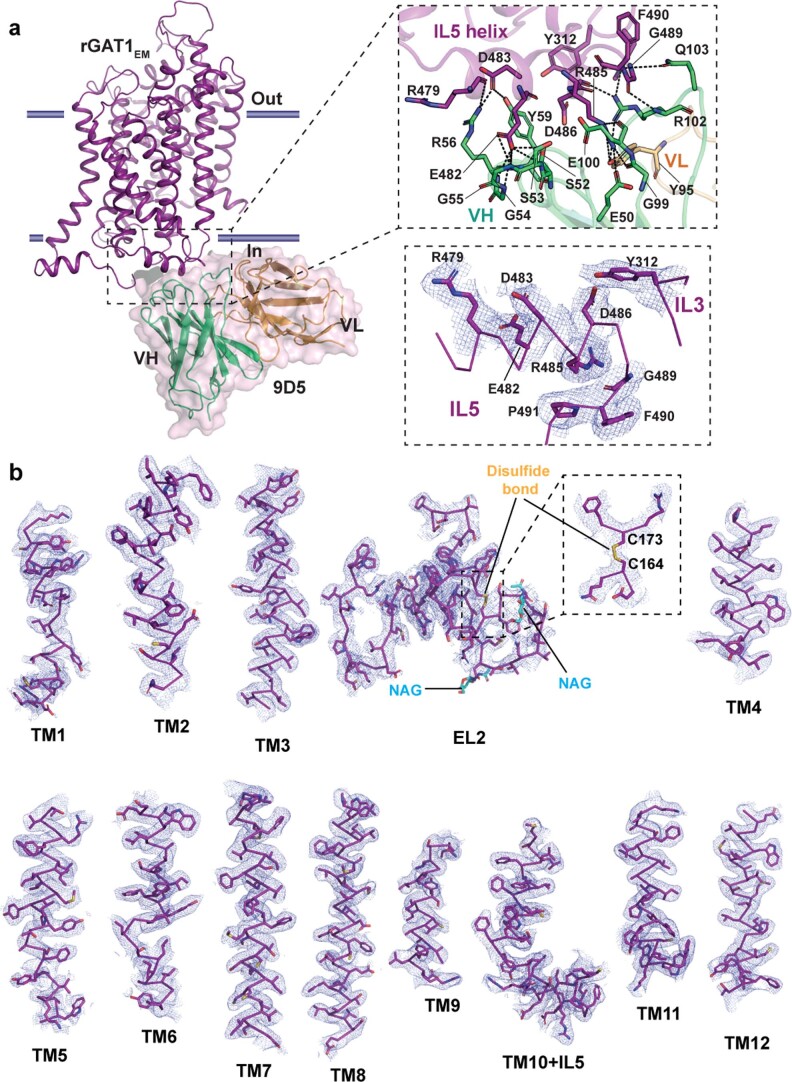
rGAT1_EM_-Fab epitope and transporter densities. **a**, Epitope interactions of rGAT1_EM_ with the variable regions of heavy and light chains 9D5 modeled into the density and **t**he epitope mutant residues are fit into unambiguous densities in the map modeled at 6.0σ. **b**, Transmembrane helices TM1-12 and extracellular loop2 of rGAT1_EM_ fit into cryo-EM density map contoured at a level (σ) of 4.5 display clear presence of side chain densities, disulfide bond and N-glycosylation sites.

The cryo-EM structure of rGAT1_EM_ complexed to a Fab was reconstructed to a resolution of 3.1 Å. The structure displays an intermediate conformation in the transport cycle (Fig. 2a,b, Extended Data Fig. 4 and Table 1) and the map quality is better than 3.0 Å in the core regions of the transporter. All transmembrane helices embedded within the detergent micelle could be modeled unambiguously at this resolution along with clear side chain densities (Fig. 2c and Extended Data Fig. 5a,b). We further modeled the extra- and intracellular loops along with two *N*-glycosylation sites in EL2 and a disulfide bridge between the conserved residues Cys164 and Cys173 into the density (Fig. 2c and Extended Data Fig. 5b). The rGAT1_EM_ structure displays a cytosol-facing conformation with a closed extracellular gate and is open to the cytosolic face (Fig. 2d). The interactions at the closed extracellular gate comprise a network of hydrogen bonds between the rocking-bundle helices of TM1 and TM6 with TM3 and TM10 and also include an interaction between EL4 and TM1 (Fig. 2d, inset).

**Table 1:** Cryo-EM data collection, refinement and validation statistics

	rGAT1_EM_ (EMD 34167) (PDB 8GNK)
**Data collection and processing**	
Magnification	105,000×
Voltage (kV)	300
Electron exposure (e^–^/Å^2^)	50.09
Defocus range (μm)	−0.5 to −4.0
Pixel size (Å)	0.831
Symmetry imposed	C1
No. of initial particle images	5,838,634
No. of final particle images	872,611
Map resolution (Å)	3.1
FSC threshold	0.143
Map resolution range (Å)	2.6–10.2
**Refinement**	
Initial model used (PDB code)	AlphaFold2 model
Model resolution (Å)	3.1
FSC threshold	0.143
Map sharpening *B* factor (Å^2^)	165
Model composition	
Non-hydrogen atoms	6,164
Protein residues	751
Water	6
Ligands	12
*N*-acetyl glucosamine	2
Ions	2
Lipids	7
GABA	1
*B* factors (Å^2^)	
Protein	63.1
Water	45.4
Ligand	86.7
*N*-acetyl glucosamine	142.9
Ions	52.7
Lipids	80.9
GABA	52.9
Root mean squared deviations	
Bond lengths (Å)	0.004
Bond angles (°)	0.66
**Validation**	
MolProbity score	1.5
Clashscore	6.0
Poor rotamers (%)	0.47
Ramachandran plot	
Favored (%)	97.0
Allowed (%)	3.0
Disallowed (%)	0

TM10 has a single residue insertion, with either Ser456 or Gly457 depending on the alignment, unique to the GABA, taurine and creatine transporters at the center of the bilayer^25,26^. The substitution widens the helix to form a one-turn π-helix, in which residue alterations hamper the uptake kinetics of GABA^25^. The side chain hydroxyl of Ser456 in TM10 interacts with the Thr290 hydroxyl in TM6 (3.4 Å) to allow extracellular gate closure (Fig. 2d, inset).

**Extended Data Fig. 6:**
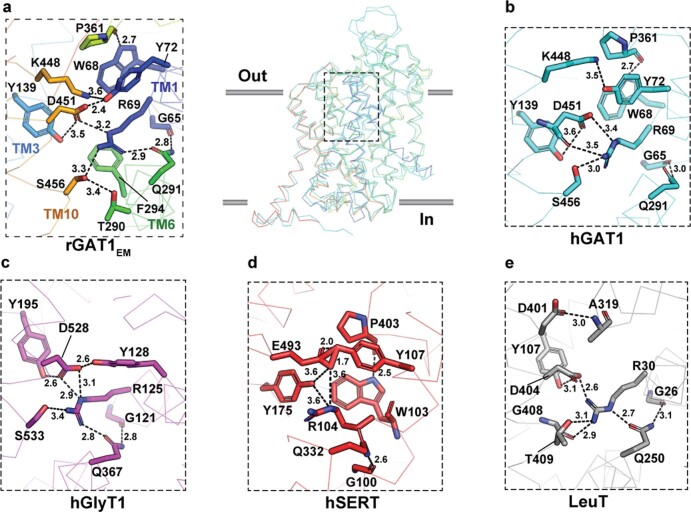
Extracellular gate H-bond network comparison. The figure displays the comparison of rGAT1 and hGAT1 with a Cα rmsd of 0.9 Å. The interactions of **a**, rGAT1 extracellular gate compared with **b**, hGAT1 (PDB id. 7SK2, Cα rmsd – 0.9 Å), **c**, hGlyT1 (PDB id. 6ZBV, Cα rmsd – 1.0 Å), **d**, hSERT (PDB id. 6DZZ, Cα rmsd – 1.3 Å) and **e**, LeuT (PDB id. 3TT3, Cα rmsd – 1.7 Å) extracellular gate H-bond network.

The interactions are similar to those of the recently determined hGAT1 structure^20^, although the side chain positions and hydrogen bond distances are modeled with greater precision in rGAT1 due to the improved resolution (Extended Data Fig. 6a,b). The hydrogen bond network in this region of GAT1 is more extensive than the inward-open structures of LeuT, hSERT and hGlyT1 (Refs. ^13,14,17^), which could facilitate extracellular gate closure in response to substrate interactions (Extended Data Fig. 6).

Residue substitutions that are vital for gating at the extracellular side, namely Arg69Lys, Gln291Asn and Asp451Glu, have previously been analyzed using the two-electrode voltage clamp methodology. Although these conservative replacements impair transport, they all exhibit sodium-dependent capacitive transient currents^27–29^. They reflect the transition between empty-inward and empty-outward conformations, preceding the binding of extracellular sodium, which stabilizes the outward-open conformation^29,30^. The voltage dependence of these transient currents indicates that the external gate mutants have an increased apparent affinity for extracellular sodium, probably as a result of a perturbed extracellular gate^27–29^. Therefore, these transporters are ‘stuck’ in an outward-open conformation and cannot cycle through inward-open conformations.

The probable movements of the extracellular gate from the outward-open state to the closed state can best be summarized by comparison of the inward-open structure with the rGAT1 AlphaFold2 model that we generated in the outward-open conformation. A structural overlap of the rGAT1 outward-open AlphaFold2 model with the cytosol-facing rGAT1 cryo-EM structure predicts the inward movement of TM1b and TM6a by 22° and 14°, which allows the formation of the close-knit hydrogen bond interactions observed in the extracellular gate of GAT1 (Fig. 2e). Beneath the compact extracellular gate, the primary substrate and inhibitor-binding site is covered by Phe294, which forms the ‘roof’ of the binding site facing the extracellular vestibule, alongside Tyr140 (Fig. 2e), in the occluded state. Phe294 undergoes a χ1 torsion angle shift of 52.4° to occlude solvent access to the binding pocket. The two residues, Phe294 and Phe293, are lowered by 1.8 Å and 1.5 Å, respectively during the angular helical movement of TM6a to close the extracellular gate. Substitutions at Phe294 are known to severely hamper the apparent affinity for GABA because of its participation in the ‘roof’ of the binding pocket, except for Phe294Tyr, which is an aromatic substitution^31^.

### Substrate and ionic interactions in the primary binding site

**Fig. 3:**
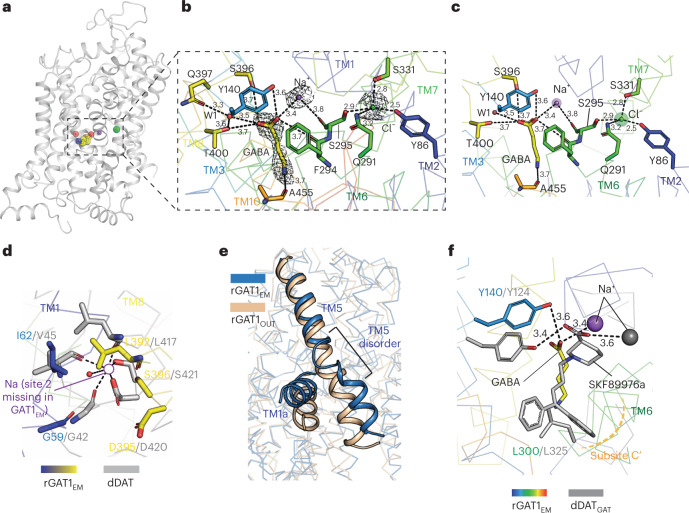
Substrate and ionic interactions in the GAT1 binding site. **a**, Lateral view of GABA and the ion-bound rGAT1 structure**. b**, Close-up of the area indicated in (**a**) showing the density for GABA, sodium ion and chloride ion bound in the primary binding site contoured at σ level of 5.0. GABA interacts with Na^+^ and with the hydroxyl side chain of Tyr140. It is in proximity to the carbonyl oxygen of Phe294 and the hydroxyl side chain of Ser396. The GABA amine group is proximal to the carbonyl oxygen of Ala455. An additional water molecule between TM3 and TM8 interacts with GABA and the side chains of Gln397 and Thr400. **c**, The sodium and chloride ions bound in the binding pocket display a weakened interaction of sodium at site 1 and a clear coordination for chloride. **d**, A comparison of sodium interactions at site 2 shows compromised interactions in rGAT1 (colored) at this site (empty circle) in comparison to dDAT (grey) (PDB 4XP1). The empty circle denotes the expected site of sodium interactions and the position of residues in rGAT1 that could interact with the ion. **e**, Sodium release at site 2 is associated with the disordered TM5 helix at the Gly-X9-Pro motif. **f**, Overlap and comparison of the GABA (yellow) binding site with the SKF89976a-bound structure of dDAT_GAT_ (PDB 7WLW; Cα r.m.s.d. – 2.7 Å). The substrate retains interactions at subsite C′ similar to competitive inhibitors.

**Extended Data Fig. 7:**
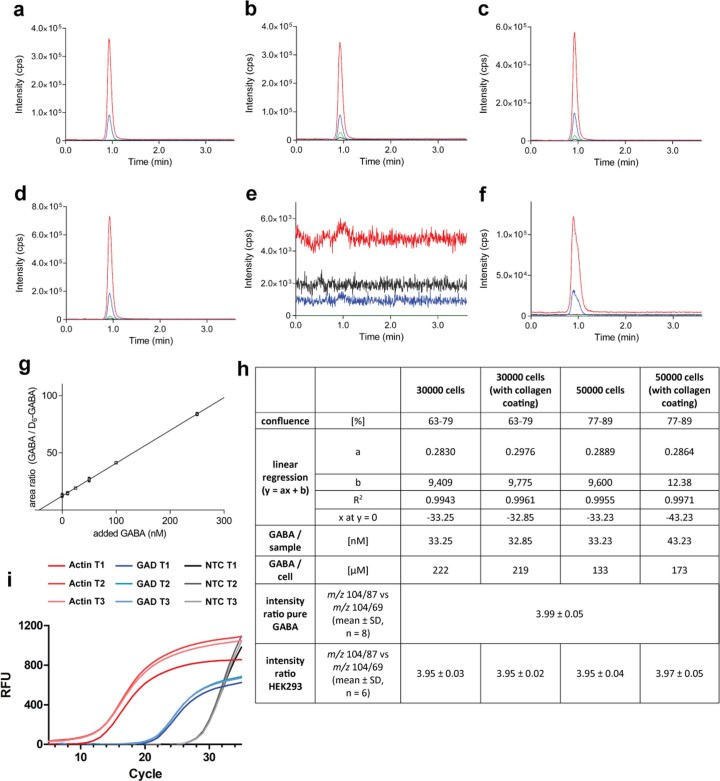
GABA detection in HEK293 cells. Chromatograms recording *m/z* 104/87 (GABA, red), 104/69 (GABA, blue), 110/93 (D_6_-GABA, grey) and 110/73 (D_6_-GABA, green) in the MRM (multiple reaction monitoring) mode. Quantification of GABA was based on the area ratios obtained for GABA and D_6_-GABA at the mass transitions *m/z* 104/87 and 110/73, respectively. **a**, Chromatogram of pure cell sample (HEK cells). **b**, Chromatogram of cell sample spiked with D_6_-GABA. **c**, Chromatogram of cell sample spiked with 25 nM GABA and D_6_-GABA. **d**, Chromatogram of cell sample spiked with 50 nM GABA and D_6_-GABA. **e**, Chromatogram of blank prepared in the absence of HEK293 cells. **f**, Chromatogram of 20 nM GABA standard prepared in the absence of HEK293 cells. **g**, Standard addition curve for GABA calculation. **h**, Table showing results of LC-MS/MS quantification to display the presence of GABA levels in HEK293 cells. **i**, rt-qPCR amplification of glutamate decarboxylase (GAD1) transcript from cDNA prepared from HEK293 cells observed around 24^th^ cycle in comparison to a control actin transcript that amplifies around 14^th^ cycle. The no transcript control (ntc) amplification is observed only after the 30^th^ cycle. Each transcript’s amplification was tested through three independent trials (T1-3). Source data

Upon structure refinement, we observed the presence of a linear density within the primary binding site. The observed density fits well for a molecule of GABA (Fig. 3a,b), although GABA was not added during the purification process. We suspected that GABA could be produced and released from the HEK293 cells that we used for expression of rGAT1. To show the presence of GABA in the HEK293 cells, we analyzed its presence directly in lysed HEK293 cells using LC-ESI–MS/MS recordings. GABA was recorded at the known mass transitions *m/z* 104/87 and 104/69 and quantified using D_6_-GABA as an internal standard according to the standard addition approach (Extended Data Fig. 7). In this way, single peaks recorded for the GABA mass transitions in the LC-ESI–MS/MS chromatograms at the retention time observed for pure GABA were detected, with an intensity ratio of the mass transitions *m/z* 104/87 versus 104/69 almost identical to the one observed for pure GABA (Extended Data Fig. 7). These peaks were enhanced by the addition of GABA to lysed cell samples without any signs of peak splitting or additional peaks. After GABA quantification in HEK293 cell samples, GABA concentrations per cell were estimated to be within the range ~130–220 µM for the different conditions investigated (Extended Data Fig. 7h). We further demonstrated the presence of the glutamate decarboxylase isoform 1 (GAD1) transcript in HEK293 cells (Extended Data Fig. 7i). This is consistent with the observation that significant levels of GABA are also observed in the kidney^32^. The native GABA released from HEK293 cells during extraction probably interacts with rGAT1_EM_.

**Extended Data Fig. 8:**
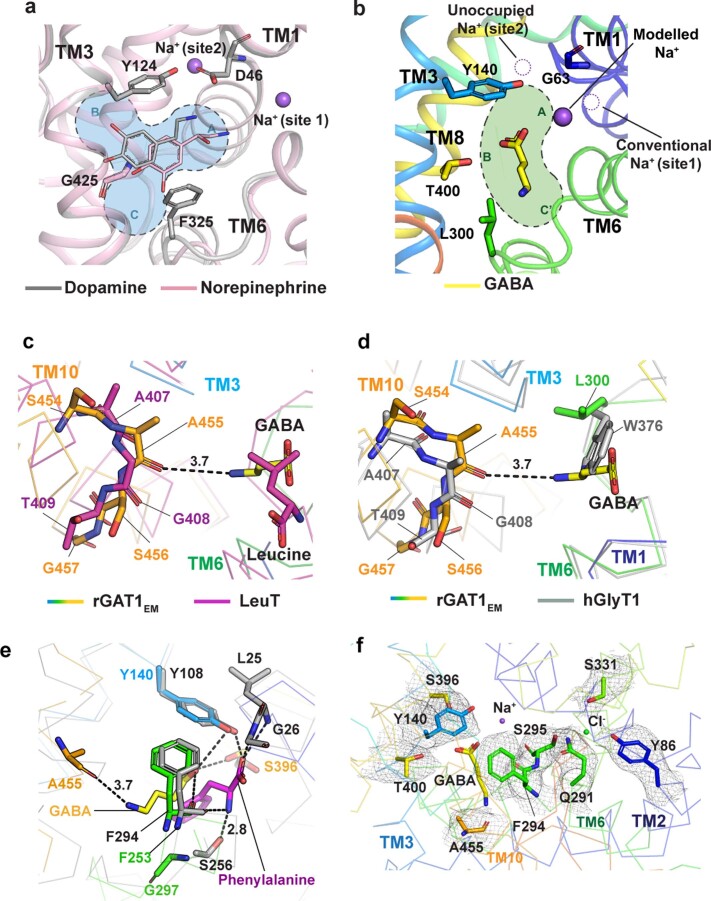
Subsite architecture of monoamine vs GABA transporters. **a**, Subsite architecture of monoamine transporters displaying a trilobed structure with dopamine fitting into subsites A and B (using PDB id 4XP1) whereas norepinephrine fitting into subsite A and subsite C (using PDB id 6M2R). **b**, GABA transporter displays a bean shaped architecture with GABA fitting into subsite A and subsite C’. **c**, The position of the modeled GABA in comparison to leucine bound LeuT (PDB id 3USK; Cα rmsd – 3.4 Å). A455 on the discontinuous region of TM10 in rGAT1_EM_ is involved in the interactions with the amine group of GABA. **d**, A comparison between hGlyT1 (PDB id 6ZBV; Cα rmsd – 1.0 Å) and rGAT1 displays the difference in the TM10 position due to insertion and presence of W376 in hGlyT1 that blocks and restricts the volume of hGlyT1 binding pocket. **e**, Comparison of rGAT1 with inward-occluded, phenylalanine bound LeuT (PDB id 6XWM; Cα rmsd – 2.5 Å) displaying the difference in interaction between α-amine group at S256 (2.8 Å) in LeuT and GABA γ-amino group with A455 carbonyl in rGAT1. **f**, Side chain densities contoured at 5.0 σ level of the residues surrounding GABA and ions in the binding pocket of rGAT1.

The primary binding site of biogenic amine transporters is represented as a group of subsites (A, B and C). The ions and substrate functional groups such as amine or carboxylate interact at subsite A, whereas functional groups that dictate affinity and specificity interact at subsites B and C (Extended Data Fig. 8a,b)^19^. In the GAT1 binding site environment, the trilobed binding site is altered to a bean-shaped binding site with subsites A and C′^21^. The substrate site is closed from the extracellular part of the vestibule but open to solvent access towards the cytosolic side. A sodium ion was modeled into the spherical density in proximity (3.4 Å) to the carboxylate of GABA (Fig. 3a–c), which is displaced from the Na^+^ coordinating site 1 observed in NSS structures. As this density appeared to be strong for a single Na^+^ ion, multiple small molecules were tested at this density for potential interactions (Supplementary Fig. 2a). Among the tested compounds, only the Na^+^ ion, present at 300 mM in the buffer, fit with the highest correlation coefficient (0.6) (Supplementary Fig. 2b). As observed in inhibitor-bound outward-open dDAT_GAT_^21^ and other SLC6 members, the Na^+^ coordination at site 1 in rGAT1 comprises the carboxyl oxygen of the substrate, the side chain oxygens of Asn66, Ser295 and Asn327 and the main chain carbonyl oxygens of Ala61 and Ser295. In the current structure, the Na^+^ density is displaced from Na site 1 by 4 Å but retains direct interaction with the carboxyl oxygen of GABA (3.4 Å) and is close to the carbonyl oxygen of Ser295 (3.8 Å) (Fig. 3c). GABA is oriented horizontally from subsite A to subsite C′ in the unwound part of TM6 (Extended Data Fig. 8b), akin to the inhibitor interactions observed in dDAT_GAT_^21^. An additional density was observed in the vicinity of the GABA carboxylate within the pocket lined by the TM3 and TM8 residues (Fig. 3b). Although a water molecule was modeled into this site that is in contact with GABA, Thr400 and Gln397, an additional cation could potentially interact at this site (Fig. 3b). The presence of this density raises the possibility of an additional Na^+^-binding site in the primary site that is suggested among GATs by one group^33^.

We observed a very clear density for chloride in the binding site coordinated by residues Gln291, Ser295 (TM6a), Tyr86 (TM2) and Ser331 (TM7) within a range of 2.5 to 3.2 Å, similar to the chloride coordination in dDAT^11^ and hSERT^12^, displaying a well-coordinated chloride ion despite the transporter shifting to an inward-open conformation (Fig. 3a–c). This is consistent with earlier observations that in contrast to GAT1_WT_, transport by the Ser331Glu mutant and Ser331Glu/Gln291X double mutants is chloride-independent^28,34^. Chloride has been suggested to aid in attracting Na^+^ to bind GAT1 and to be symported with GABA^34,35^, but subsequent studies have indicated that the interaction of chloride with its coordinating glutamine residue allows the formation of an ion pair between TM1 and TM10 that closes the extracellular pathway^28,36^. In the rGAT1 structure, the distance between chloride and modeled Na^+^ is larger (8 Å) than in the other NSS structures (5–6 Å), suggesting that the cation has started to move. In contrast to sodium site 1, there was no evidence of any density at sodium site 2, where the coordination for Na^+^ is disrupted due to conformational changes in TM1, TM5 and TM8 (Fig. 3d and Extended Data Fig. 8b). The coordinating residues in dDAT_GAT_ and throughout the NSS family correspond to Gly59 and Ile62 in TM1, and Leu392, Asp395 and Ser396 in TM8 of rGAT1 (Fig. 3d). Interestingly, Asp395 is implicated in interacting with Li^+^ ion at site 2 during cationic leak currents, and this cation can support transport if Na^+^ is present at low concentrations to fill the site 1 (Ref. ^37^). TM5 unwinds at the Gly-X_9_-Pro motif leading to increased solvent access to sodium site 2 (Fig. 3e). The solvation of sodium at site 2 and its release was proposed to be required for the transition of LeuT-fold transporters, such as MhsT, from outward- to inward-open conformation^38^. Consistent with this hypothesis, we observed rGAT1 to have a GABA coordinating sodium displaced from its conventional coordination site 1, a bound chloride and an empty sodium site 2 in the cytosol-facing rGAT1_EM_.

The single molecule of GABA in the binding pocket interacts with the carbonyl of Phe294 via one of its carboxyl oxygens, which forms the roof of the primary site, at a distance of 3.7 Å (Fig. 3b). The carboxylate is proximal to the hydroxyl groups of Tyr140 (3.6 Å) in TM3 and Ser396 (3.7 Å) in TM8 alongside Thr400 (TM8) at a distance of 3.7 Å and occupies the region referred to as subsite A next to Gly63 (TM1), similar to amino acid transporters such as LeuT^10^. Consistent with its importance in substrate recognition, Tyr140 (Ref. ^39^), the GABA carboxylate group, is engaged in direct interactions with the Tyr140 hydroxyl (3.6 Å) and the ion density. A distance of 4.2 Å separates the Tyr140 hydroxyl and the sodium ion, and the GABA carboxylate is involved in bridging Tyr140 with the modeled Na^+^ ion (3.4 Å) (Fig. 3c). The amine group faces subsite C′ at the unwound part of TM6 and is close to the carbonyl group of Ala455 in TM10 (3.7 Å) (Fig. 3b and Extended Data Fig. 8c,d). Interestingly, Ala455 is proximal to the Ser456/Gly457 insertion that forms a π-helix in TM10 found in GATs. A comparison with the hGlyT1 structure shows the presence of Trp376 (equivalent to Leu300 in GAT1 in the TM6 unwound region) occupying the substrate-binding pocket of GABA; it is required to minimize the volume of the binding site to accommodate a small amino acid such as glycine (Extended Data Fig. 8d)^13,21^.

**Extended Data Fig. 9:**
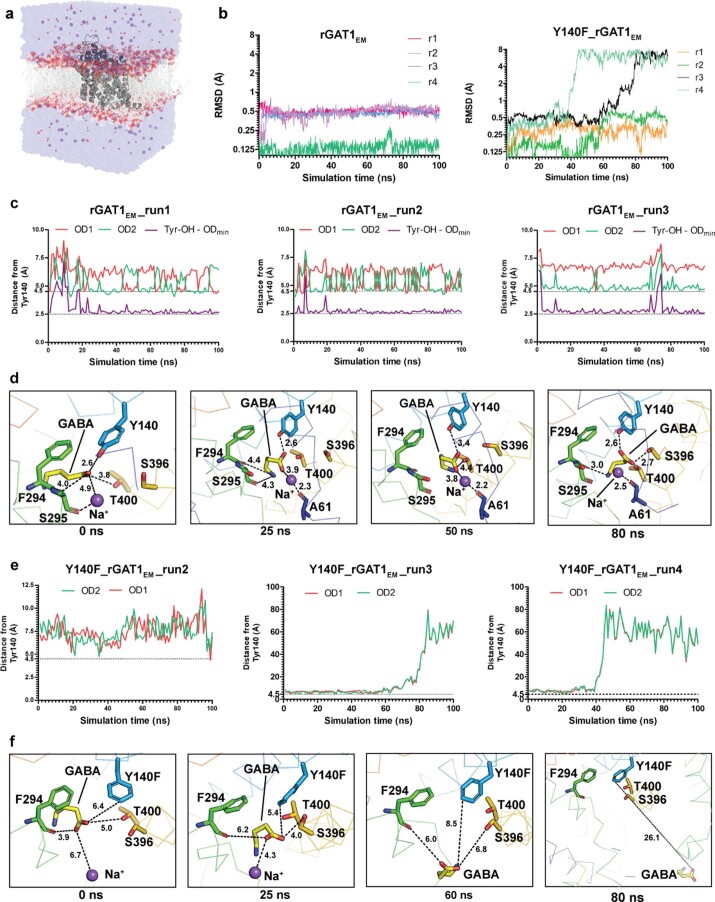
Simulation of GABA in the binding pocket of rGAT1. **a**, A snapshot of the simulation box assembled for rGAT1_EM_. White sticks represent the lipid bilayer with phosphate headgroups shown as spheres. Suspended in water (transparent surface) are sodium and chloride ions, shown as spheres. rGAT1_EM_ is represented as a cartoon. **b**, RMSD traces of GABA against its modeled position in 4 runs of rGAT_EM_ and its Y140F mutant each, respectively. RMSD axis is shown in logarithmic scale for clarity. **c**, Distance traces between the centroid of Y140 and the two carboxylic oxygens (OD1 as dark red and OD2 as bright red) of GABA shown in three representative MD runs in rGAT1_EM_, with the distance between Y140 hydroxyl group and one of the carboxylate oxygens of GABA shown in black traces. **d**, Snapshots of GABA binding pocket at different timepoints during the course of simulation in one of the runs. Dotted lines demarcate the distances between the pair of atoms. GABA is shown in sticks and transparent spheres. **e**, Distance traces between the centroid of Y140 and the two carboxylic oxygens (OD1 as dark red and OD2 as bright red) of GABA shown in three representative MD runs in the Y140F mutant, and snapshots of one such run are shown in **f**, where distances are measured from the *Cz* atom of F140 to the closest carboxylic oxygen of GABA instead, along with other residues in the binding pocket. Source data

We performed molecular dynamics simulations of zwitterionic GABA-bound rGAT1 to evaluate its stability in the binding pocket. During the simulation, we observed the GABA carboxylate shifting closer to subsite A to form hydrogen bond interactions with the hydroxyl of Tyr140. It should be noted that the simulations were performed in a lipid bilayer, where these transporters are expected to be dynamic. Despite this, GABA remains relatively stable in the binding pocket. In the simulations, the enhanced proximity of the GABA carboxylate towards Tyr140 weakens interactions of the GABA amine group with Ala455 carbonyl and interacts with surrounding residues, particularly with the side chain of Thr400 and Ser396. Consequently, Tyr140Phe substitution led to a weakened GABA binding network, with GABA being extremely flexible or moving away from the binding pocket in the simulation time scales (Extended Data Fig. 9). Interactions of this carboxylate moiety with the equivalent of Tyr140 (-OH) were observed with the inhibitor–dDAT_GAT_ complexes (Fig. 3f) and the outward-open and outward-occluded structures of other NSS amino acid transporters^21^.

Comparison of the present structure with those of other members of the NSS family (and the outward-open AlphaFold2 rGAT1 model) suggests that the sodium ion, together with GABA, has started to move towards the cytoplasmic pathway, whereas the chloride ion still occupies its original position.

### Cytosolic gate opening allows solvent access to substrate

**Fig. 4:**
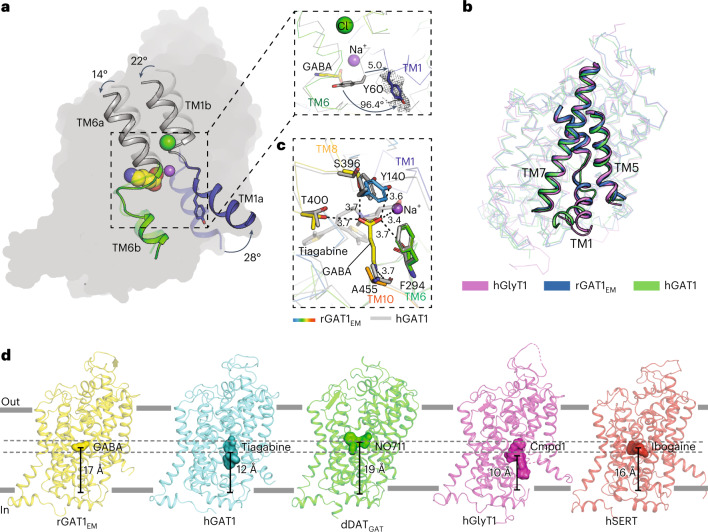
Cytosolic gate opening allows solvent access to substrate. **a**, The solvent access in rGAT1 is facilitated by opening of the TM1a like a trapdoor by 28° and a corresponding shift of Tyr60 by 96.4° in comparison to the outward-open AlphaFold2 model of rGAT1 (inset). **b**, The movement of TM1a is similar to that by hGlyT1 (PDB 6ZBV; Cα r.m.s.d. – 1.0 Å) and is not biased towards TM7 as observed with hGAT1 (PDB 7SK2; Cα r.m.s.d. – 0.9 Å). **c**, GABA (yellow) and tiagabine (grey) have primary interactions in subsite A. Tiagabine wedges in the solvent accessing the cytosolic vestibule to cause a non-competitive block of uptake. **d**, Variation in binding-site position in SLC6 transporter structures bound to substrate or inhibitors. The center of mass of the ligands GABA, tiagabine, NO711, Cmpd1 and ibogaine were calculated in PyMOL and were used to measure the distance of the ligands from the surface of a predicted membrane bilayer. The rGAT1_EM_ structure was inserted into a membrane bilayer containing cholesterol, POPC and POPE lipids. The head groups of lipids in the lower leaflet provided the *z*-axis coordinates to calculate the distance of the center of mass of ligands from the bottom of the membrane.

Comparison of the inward-open structure with our AlphaFold2 model leads us to suggest that solvent access from the cytoplasm to the substrate-binding site is facilitated by the shifts in the unwound regions of TM1 and TM6 followed by the opening of the cytosolic gate through the angular movement of TM1a (Fig. 4a). Owing to the transition from outward- to inward-open states, the primary binding site position is also lowered towards the cytosolic half, reflected in a 3–4 Å downward displacement in the positions of TM1 and TM6 in addition to the angular movement observed in overlaps of the outward-open model of rGAT1 with the cytosol-facing structure of rGAT1_EM_ (Fig. 4a). TM1a is the primary gating helix and shows behavior consistent with that of other members of the SLC6 family. Although the density of TM1a is weaker compared to other regions of the molecule, we were able to model it into the visible density from residues 50 to 60. In rGAT1, TM1a moves out by an angle of 28° to solvate the intracellular vestibule and substrate-binding site (Fig. 4a). Unlike hGAT1, for which TM1a is closer to TM7, we observed a centered position of TM1a similar to hGlyT1 and hSERT (Fig. 4b). This is possibly a consequence of deleting a stretch of N terminus in our molecule, whereas the hGAT1 construct has a complete N terminus. TM1a could also be dynamic in its position and sample multiple local shifts in its position through the transport cycle. The corresponding residue of Tyr60 forms the floor of the binding site in the outward-open conformations of NSS members such as dDAT (Phe43) and hSERT (Tyr95). The substitutions at Tyr60 result in compromised transport activity and increased cationic leak currents, emphasizing its role in gating GABA and Na^+^ movement^27^. In rGAT1, the TM1 unwound region becomes disordered and flips the Tyr60 side chain downwards by ~96° in a trapdoor-like fashion to allow solvent access (Fig. 4a, inset). The movement of TM1a opens the cytosolic gate centered between TM5 and TM7 (Fig. 4b and Supplementary Fig. 3). The presence of an open cytosolic gate could be facilitated by substitutions of hydrophobic residues observed in rGAT1 in comparison to other biogenic amine transporters such as hSERT. For instance, Phe347 (hSERT) is substituted to a leucine (Leu306, TM6b) in rGAT1. Similarly, cysteine residues are observed in rGAT1 (Cys57 (TM1a), Cys102 (TM2)) instead of the hydrophobic residues in hSERT (Val92, Leu137) (Supplementary Fig. 3b,c). These substitutions could enhance the propensity of rGAT1 to attain a cytosolic-facing conformation in micelles.

**Extended Data Fig. 10:**
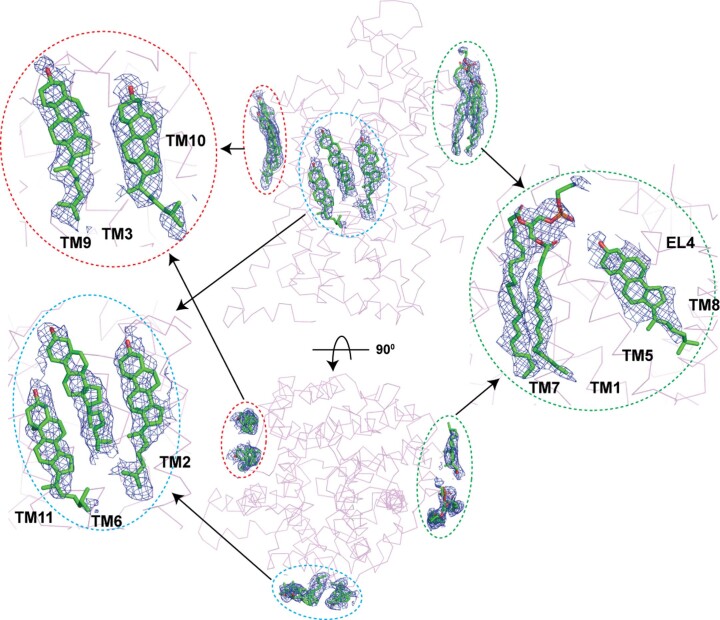
Annulus of lipids observed around rGAT1 structure. Lipid densities (contoured at 6.0 σ) surrounding rGAT1_EM_, fitted with cholesterol molecules present both in the extracellular as well as cytoplasmic half of the membrane and one POPE molecule in the extracellular half. Cholesterol was however not observed near the TM1a likely due to its inward-open conformation. TMs in proximity to lipid densities are labeled.

As described earlier, TM5 displays enhanced flexibility near the Gly-X_9_-Pro region to facilitate Na^+^ release from site 2 as observed in MhsT^38^. The GABA position in rGAT1 is lower than the inhibitor-bound, outward-open dDAT_GAT_ structures as a consequence of its transition to the inward-open state. The extra bulk of tiagabine bound to hGAT1, that of the bitopertin analogue, cmpd1 bound to hGlyT1 and ibogaine-bound hSERT extends to positions that are proximal to the cytoplasm (Fig. 4c,d). In reconstituted systems, GABA transport activity was found to be highly dependent on the addition of cholesterol to the phospholipid mixture^40^. Consistent with this, we observed an annulus of lipid densities in the GAT1_EM_ structure, into which we modeled multiple cholesterol molecules and a phospholipid (Extended Data Fig. 10). Although cholesterol associates with TM1a in dDAT^11^, we did not observe a cholesterol molecule bound atop TM1a in rGAT1, possibly due to its displacement during TM1a opening.

## Discussion

The high-resolution structure of rGAT1 obtained in this study was facilitated by the interactions of the Fab fragment with rGAT1 after epitope transfer from dDAT. This approach has not been used in NSS members before this study, raising the possibility of using this strategy to reconstruct homologous transporter structures using cryo-EM.

The present study of rGAT1 and an earlier study on hGAT1 revealed a cytosol-facing state of the transporter^20^, although structures of the related transporters dDAT, hSERT and LeuT were all in outward-open or outward-occluded states^10–12^. In both dDAT and hSERT X-ray structures, extensive thermostabilization of the transporters was performed to stabilize the inhibitor-bound, outward-open conformation^11,12^. There was no such conformation stabilization used in the cytosol-facing cryo-EM structures of hSERT^17,41^ or rGAT1. In LeuT, it was possible to obtain an inward-occluded structure by perturbing the intracellular gate and using a bulky substrate to form a complex^42^. Mutations at the intracellular gate together with perturbation of the Na2 site yielded an inward-open, substrate-free structure of LeuT^14^. From cysteine modification studies, we can infer that GABA interactions in rGAT1 close the extracellular gate^36,43^. The presence of a cytosol-facing conformation could be facilitated by the formation of a π-helical segment in TM10. The motif accentuates the hydrogen bond network in GAT1, making it stronger than other members of the SLC6 family, and participates in substrate interactions with bound GABA (Extended Data Fig. 8c,d).

In the structure, GABA directly interacts with a sodium ion, a hydroxyl of Tyr140 and a carbonyl of Phe294 that gates solvent access to the substrate-binding site (Fig. 3b). Similar localization of serotonin with interactions at the subsite A aspartate (Asp98) are observed in the cytosol-facing hSERT structure^41^. Simulations of zwitterionic GABA within the binding pocket display the formation of hydrogen bond interactions of carboxylate with Tyr140 (Extended Data Fig. 9d), consistent with its role in substrate recognition^39^. The γ-amino group is located within subsite C′ in the TM6 linker, facilitated by the substitution of phenylalanine (Phe325, dDAT) in monoamine transporters to leucine (Leu300) in GAT1 (Extended Data Fig. 8a,b). In LeuT, which transports α-amino acids, the substrates interact with the hydroxyl of Ser256 (TM6), which is altered to glycine in GAT1 (Gly297), thereby compromising this interaction (Extended Data Fig. 8e,f). We speculate that the location of the γ-amino group of GABA close to TM10 accommodates the distance constraint on GABA in the binding pocket. The bound chloride ion in rGAT1 has a stable tetrahedral coordination similar to that in other SLC6 transporters. This is consistent with earlier electrophysiology studies wherein Cl^˗^ was suggested to dissociate into the cytosol after GABA and sodium ions^44^. The density for Na^+^ is missing at site 2 due to solvent access at this site resulting from the unwinding of TM5 at the Gly-X_9_-Pro region and opening of TM1a. Solvation and release of sodium from site 2 is proposed to initiate the release of the substrate to the cytosol^38,42^, and the rGAT1_EM_ structure in this study is consistent with this idea. This step is followed by an outward movement of TM1a, resembling a trapdoor, to allow solvent access to the binding site from the cytosol (Fig. 4a, inset).

**Fig. 5:**
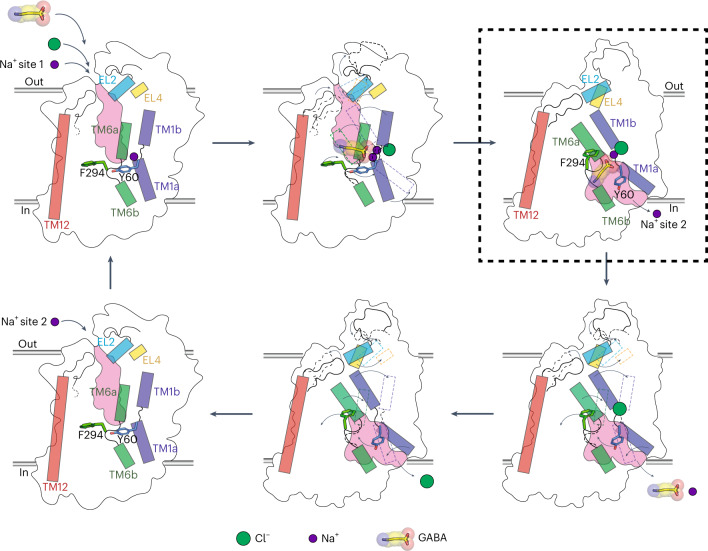
Schematic of the transport cycle of rGAT1. The cycle is shown in the forward mode, GABA uptake. The outward-open empty transporter (bottom panel, left) binds a sodium ion (to the Na2 site), resulting in the stabilization of the outward-open conformation (top panel, left) followed by the second sodium ion (to the Na1 site), a chloride ion and GABA to yield the fully loaded outward-open transporter (top panel, middle). This is followed by occlusion of GABA and the co-transported ions (not shown) and, subsequently, the loaded transporter becomes inward facing and releases the sodium from the Na2 site to the cytoplasm (top panel, right). This state represents the structure reported in this study, highlighted with a black box with dashed lines. Subsequently, GABA and the sodium ion from the Na1 site follow (bottom panel, right) before the release of the chloride ion (bottom panel, middle). Upon isomerization to the outward-open conformation (bottom panel, left), a new translocation cycle can begin.

Based on multiple biochemical observations and the structural comparisons between an rGAT1 model in an outward-open conformation and the cryo-EM structure in the cytosol-facing state, we propose a refined GABA reuptake cycle (Fig. 5). The first step is a major conformational change, whereby the empty inward-open transporter (Fig. 5, bottom panel, middle) rearranges to the outward-open conformation (Fig. 5 bottom panel, left) that is stabilized by Na^+^ binding to site 2 (Fig. 5, top panel, left), connecting TM1 of the bundle with TM8 of the scaffold^45^. Subsequently, another sodium at the Na1 site, chloride and GABA bind to yield the loaded outward-open transporter (Fig. 5, top panel, middle). This is followed by another major conformational transition to the inward-open state. This step is also the result of connecting the bundle with the scaffold; however, here it is not Na2 but the amino acid substrate that bridges between the two domains at a different location within the transporter structure. The carboxyl group of the amino acid substrate connects a Na^+^ ion bound at the Na1 site, located on the bundle, with the hydroxyl group of Tyr108 (in LeuT, 140 in GAT1) from TM3, which is part of the scaffold^46^. The rGAT1_EM_ structure described in this work provides a glimpse into the substrate translocation pathway by capturing an intermediate inward-open state from which the sodium ion from the Na2 site has been released to the cytoplasm (Fig. 5, top panel, right). In this conformation, GABA is beginning, alongside a sodium ion, to move towards the cytoplasmic pathway, to be released in a subsequent step (Fig. 5, bottom panel, right). Finally, the chloride dissociates to yield the empty inward-open transporter (Fig. 5, bottom panel, middle), which isomerizes to the empty outward-open conformation (Fig. 5, bottom panel, left) and a new reuptake cycle can begin. In the bacterial NSS members LeuT and MhsT, substrate-bound structures have been found in which an access path for Na^+^ to the cytosol has started to open up^38,42^. The structure described here goes one step further in the cycle because the Na^+^ from site 2 has already been released.

The high-resolution structure of rGAT1 presented in this study can serve as a template for accurate docking and inhibitor-interaction studies to identify GAT1-specific competitive and non-competitive inhibitors in the future. Structural studies of other conformations of GAT1 are expected to deepen our insights into the mechanistic details of GABA transport and the inhibition of GAT1.

## Methods

### Construct design

The rGAT1 was cloned into pEG BacMam to generate all constructs in this study, including rGAT1_WT_, the rGAT1_Δ38_ construct containing N-terminal deletion of 37 amino acids (Δ2–38). All constructs have a carboxy-terminal thrombin site, followed by GFP and 8×-His tag. The epitope library was generated on a wild-type background by introducing 11 dDAT-like mutations into the IL5 helix, IL3 loop, IL1 loop and C-terminal loop in various combinations. The final construct of rGAT1_EM_ used for cryo-EM contains Δ2–38 N-terminal deletions along with 9 epitope transfer mutations: Tyr481Ser, Asp482Glu, Asn483Asp, Gln485Arg, Glu486Asp, Val488Ile, Ser490Phe, Arg491Pro and Phe312Tyr. Site-directed mutagenesis was performed through whole-plasmid amplification using mutant primers to introduce substitutions into the rGAT1 construct, followed by DpnI digestion at 37 °C and bacterial transformation (Supplementary Table 2). All constructs were cloned into the pEG BacMam vector between the EcoRI and NotI restriction sites for biochemical studies and cryo-EM.

### Fluorescence-detection size-exclusion chromatography

Fluorescence-detection size-exclusion chromatography (FSEC) was carried out using an HPLC system attached to a multi-wavelength fluorescence detector (RF20A, Shimadzu) and a refrigerated autosampler (SIL-20ACHT, Shimadzu). We monitored tryptophan fluorescence (excitation, 294 nm; emission, 334 nm) in the case of purified protein and GFP fluorescence (excitation, 488 nm; emission, 510 nm) in whole-cell solubilized samples in which constructs were GFP-tagged. To check for 9D5 binding among epitope mutants, 1 × 10^6^ HEK293S cells transfected with 2 µg of plasmid DNA for each mutant were solubilized in detergent (20 mM *n*-dodecyl-β-d-maltopyranoside (DDM) + 2 mM cholesteryl hemisuccinate (CHS) (Anatrace)) and were run on FSEC post incubation with 50 ng of 9D5. The leftward shift in the elution volume was monitored for each epitope mutant. Peak shift upon 9D5 addition in dDAT was used as a positive control, and 100 µM LMNG in 50 mM Tris pH 8.0 and 150 mM NaCl was used as a buffer for FSEC runs on a Superose 6 Increase 10/300 GL column.

### Heterologous expression and purification of GAT1

The recombinant expression of rGAT1_EM_ in the pEG BacMam vector was carried out in HEK293S GnTI^-^ cells using the baculovirus-mediated mammalian cell expression system^47^. The protein was extracted from membranes using 20 mM DDM, 2 mM CHS, 50 mM Tris pH 8.0 and 150 mM NaCl. The solubilized material was separated by ultracentrifugation at 111,000×*g* for 90 min in an SW32 Ti rotor (Beckman Coulter). The supernatant was incubated with cobalt-charged affinity resin (Takara Bio), and the transporter was eluted in 50 mM Tris pH 8.0, 300 mM NaCl, 100 mM imidazole containing 1 mM DDM and 0.1 mM CHS. The purified protein was treated with thrombin (Haematologic Technologies) to remove the GFP-8×-His tag from the C-terminal end. The thrombin-cleaved protein was subjected to size-exclusion chromatography purification by injecting into a Superose 6 Increase 10/300 GL column (GE Life Sciences) pre-equilibrated with buffer containing 50 mM Tris pH 8.0, 300 mM NaCl, 2% glycerol, 1 mM DDM and 0.1 mM CHS.

### Purification of Fab

Fab 9D5 was heterologously expressed in Sf9 cells (Gibco, 11496015) using the baculovirus-mediated insect cell expression system^19^. The heavy and light chains were synthesized (Genscript) and cloned into pFastBac Dual vector with N-terminal GP64 signal peptide to export the protein into the media. Due to the absence of GFP in the construct, the volume of virus used for infection of SF9 cells was optimized based on small-scale infections at different volume ratios of virus to SF9 culture volume, and protein yield was observed as shifts in the FSEC peak of the target transporter tagged to GFP induced by 9D5 in the supernatant of the small-scale cultures. Based on these estimates, 10–15 ml of P3 recombinant baculovirus was added to 2 Erlenmeyer tissue culture flasks, each with 800 ml of SF9 cells at a cell density of 2.2 million per ml. The cells were collected 96 h post infection. The supernatant was dialyzed against 25 mM Tris pH 8.0 and 50 mM NaCl, and the Fab was purified from the dialysate using Ni-NTA affinity chromatography. The protein was eluted in 25 mM Tris pH 8.0 and 50 mM NaCl containing 250 mM imidazole and further purified using size-exclusion chromatography in a Superdex 75 10/300 GL column (GE Life Sciences) pre-equilibrated in 25 mM Tris pH 8.0 and 50 mM NaCl^19^.

### Cryo-EM sample preparation and data collection

Purified rGAT1_EM_ was complexed with purified 9D5 Fab at a molar ratio of 1:1.3, and the complex was concentrated to 3 mg ml^–1^ using a 100 kDa concentrator. A 3 µl aliquot of the sample was added to glow-discharged Quantifoil holey carbon grids (gold 0.6/1.0, 300 mesh). After a wait time of 10 s, the grids were blotted for 5.5–6.0 s (100% humidity, 16 °C temperature) using an FEI Vitrobot Mark IV. Grids were sent for data collection to the eBIC Diamond Light Source facility, Oxford, UK. Images were acquired using the EPU (AFIS compatible3) acquisition software (version 2.14) on a Titan Krios at 300 keV equipped with the latest generation Ametek-Gatan BioQuantum K3 detector and energy filter operating at 20 eV slit width. Movies were recorded at a magnification of 105 kx in super-resolution mode with 2× binning, resulting in a pixel size of 0.831 Å per pixel with an exposure of 2.7 s dose-fractionated into 50 frames, resulting in a total dose of 50.09 e^-^/Å^2^. The defocus values ranged from −0.5 to −4.0 µm.

### Cryo-EM data processing

Single-particle analysis of the cryo-EM data was performed using the software package cryoSPARC^48^. A total of 18,152 micrographs were motion-corrected using patch motion correction (maximum alignment resolution set to 3 Å) and CTF parameters were determined using patch CTF estimation (maximum resolution set to 3 Å). Exposures were curated to remove poor-quality micrographs to finally obtain 14,410 micrographs. Particles were picked using reference-free blob picker (minimum particle diameter, 120 Å; maximum particle diameter, 180 Å) followed by particle extraction (particle box size, 360 pixels) and 2D classification of 5,838,634 particles obtained from 14,410 micrographs. After multiple rounds of 2D classification, we selected 1,158,303 particles for ab-initio reconstruction. A set of 1,010,553 particles belonging to two similar 3D classes displaying transmembrane helices were merged and further classified into five classes using ab-initio reconstruction followed by heterogenous refinement. Particles from three classes (872,611 particles in total) were combined and subjected to non-uniform refinement, yielding a resolution of 3.1 Å (Ref. ^49^).

### Model building and refinement

The AlphaFold2 model of rGAT1_WT_ in the outward-open conformation was used to model into the cryo-EM density map of rGAT1_EM_. The model was fitted into the cryo-EM map in UCSF Chimera (version 1.15). The Cα main chain was manually adjusted in Coot (version 0.9.6) to fit the density map^50^, resulting in a model displaying an inward-open conformation. The density for the variable heavy–variable light (VH–VL) region of Fab was complete, whereas partial density was obtained for the constant regions of the Fab. The model for the VH–VL region of 9D5 Fab was obtained from the dDAT crystal structure in complex with Fab 9D5 (PDB 4XP1) and fitted into the cryo-EM density in UCSF Chimera. The residues of rGAT1_EM_ and the Fab VH–VL region were adjusted in Coot, and the model was iteratively refined in phenix.real_space_refine (version 1.20.1)^51^.

### GABA uptake and inhibition assays

GABA uptake assays were performed using HEK293S GnTI^-^ cells expressing rGAT1_WT_, rGAT1_EM_ and rGAT1_Epi4_ constructs, and the assay was carried out in a 96-well plate format^52^. Cells were infected with the baculovirus of the respective construct, and 50,000 cells per well were plated 36 h post infection. The medium was aspirated after 4 h, and 25 µl of uptake assay buffer containing 25 mM HEPES-Tris pH 7.1, 130 mM NaCl, 5 mM KCl, 1 mM CaCl_2_ and 5 mM d-glucose was added. GABA uptake was started by incubating with 25 µl of varying concentrations of GABA (0.5, 1, 2, 4, 8, 10, 20 and 40 µM final concentration) in 1:500 molar ratios of ^3^[H]-GABA (Perkin Elmer) and unlabeled GABA. After 15 min, the uptake was arrested by adding 200 µl of ice-cold assay buffer and washing the cells twice with the same buffer before solubilization. The cells were solubilized in 100 µl of 20 mM DDM and 2 mM CHS for 1 h. Then, 50 µl of scintillation fluid (Ultima Gold, Perkin Elmer) was added to the solubilized material and the radioactivity was measured by scintillation counting using a MicroBeta liquid scintillation counter. The activity measured from uninfected cells was considered as the background. The background-subtracted initial uptake rates were plotted against different concentrations of GABA, and *K*_m_ and *V*_max_ values were determined using the Michaelis–Menten equation. GABA uptake assay was performed in two independent experiments, each with three technical replicates. Data were plotted and analyzed using GraphPad Prism (version 5.0.1).

For determination of the *K*_*i*_ value, the infected cells were incubated with 25 µl of uptake assay buffer containing varying concentrations of tiagabine and NO711 (0.001, 0.01, 0.03, 0.1, 0.5, 1, 3, 10, 50 and 100 µM) for 30 min, followed by the addition of 25 µl of ^3^[H]-GABA and unlabeled GABA in 1:250 molar ratios. After 15 min, the uptake was arrested with 100 µl of ice-cold assay buffer and washed two times with the same buffer. The cells were solubilized for 1 h in 100 µl of 20 mM DDM and 2 mM CHS, and 50 µl of scintillant was added to the solubilized material. Counts were measured using a MicroBeta scintillation counter. Uninfected cells were used as a control. The background-subtracted dose–response curves were plotted as log [inhibitor] versus response using GraphPad Prism (version 5.0.1) and IC_50_ values were determined. The *K*_*i*_ values were calculated using Cheng-Prusoff’s equation. GABA uptake inhibition assays were performed in two independent experiments with three technical replicates each. The data were analyzed using GraphPad Prism (version 5.0.1).

### Membrane preparation for mass spectrometry binding assay

HEK293S GnTI^-^ cells (ATCC, CRL3022) were grown in 800 ml of FreeStyle 293 expression medium (Gibco) and infected with recombinant baculovirus of GAT1_WT_ and GAT1_EM_ constructs at a cell density of 2.5–3.0 million cells per ml. Cells were collected after 60 h of infection and resuspended in 40 ml of 1× TBS buffer containing 50 mM Tris pH 8.0 and 150 mM NaCl. The cell suspension was sonicated, the lysate was centrifuged at 21,000×*g* to separate the cell debris and 1 ml of the supernatant was aliquoted into each of forty 1.5 ml microfuge tubes. The membrane suspension was spun at 49,000×*g* for 90 min in a TLA100.1 rotor (Beckman) followed by flash-freezing and storage at −80 °C.

### Mass spectrometry binding assay

Binding experiments were performed as described unless stated otherwise^24^. Aliquots of rGAT1 or hGAT1^53^ membrane preparations were defrosted, diluted in 20 ml 50 mM Tris-citrate buffer containing 1 M NaCl, pH 7.1 (assay buffer) and centrifuged (50,000*×g*, 4 °C, 20 min). After resuspension of the pellet in assay buffer, saturation experiments with NO711 were performed in triplicate samples (about 10 µg protein) in a total assay volume of 250 µl. Total binding was determined at ten concentration levels (2.5 nM, 5 nM, 10 nM, 20 nM, 40 nM, 80 nM, 160 nM, 320 nM, 640 nM and 1280 nM) and non-specific binding (in the presence of 100 mM GABA) was determined at six concentration levels (40 nM, 80 nM, 160 nM, 320 nM, 640 nM, 1280 nM; based on the results for these concentrations, non-specific binding for the lower concentration levels were calculated by extrapolation after linear regression). Incubation was performed for 40 min at 37 °C; subsequently, 200 µl aliquots of the binding samples were transferred onto 96-well filter plates and filtrated under vacuum, followed by rapid washing of the filters with ice-cold 0.9% NaCl (3 × 150 µl). The filter plates were then dried (60 min, 50 °C) and eluted with 3 × 100 µl methanol (containing 1.43 nM D_10_-NO711). After addition of 130 µl 10 mM ammonium formate pH 7.0 (AF-buffer) to the eluates, NO711 (bound to GAT1) was quantified by LC-ESI–MS/MS (NO711, *m/z* 351/180; D_10_-NO711, *m/z* 361/190) using a QTRAP5500 triple quadrupole mass spectrometer (Sciex) coupled to an Agilent 1260 HPLC system (Agilent) and a SIL-20A/HT autosampler (Shimadzu) controlled by Analyst software (version 1.6.3) (Sciex) with a mobile phase consisting of AF-buffer and acetonitrile (50/50, v/v) at a flow rate of 350 µl min^–1^ and an injection volume of 10 µl. Analysis of the binding data was performed with GraphPad Prism (version 6.0.7). The results are based on at least three independent saturation experiments for each GAT1 species.

### GABA quantification in HEK293 cells

HEK293 cells (Leibniz Institute DSMZ–German Collection of Microorganisms and Cell Cultures) were cultured in DMEM supplemented with 10% fetal calf serum, 100 U ml^–1^ penicillin and 100 µg ml^–1^ streptomycin in a humidified atmosphere (95% air, 5% CO_2_) at 37 °C (DMEM and all other cell culture additives were from Sigma). Cells were seeded in NuncIon Delta Surface 96-well cell culture plates (Thermo Fisher Scientific) with or without collagen surface coating (100 µl of 10 µl ml^–1^ Collagen G per well, 30 min) in 250 µl medium and cultivated overnight. The next day, the medium was carefully aspirated, 154 mM ammonium acetate buffer was added (200 µl per well), confluence was controlled with a Spark Cyto cell imager (Tecan), the ammonium acetate buffer was again carefully aspirated, and subsequently, cells were lysed by addition of acetonitrile (200 µl per well). Replicates consisting of six wells were supplemented with 50 µl of 5 mM ammonium bicarbonate or 50 µl of 125 nM D_6_-GABA ([^2^H_6_]GABA, Sigma Aldrich) or 50 µl of 125 nM D_6_-GABA together with defined GABA concentrations (50 nM, 125 nM, 250 nM, 500 nM or 1250 nM), to yield pure cell samples, zero samples (containing only internal standard) and cell samples with defined added concentrations of GABA and D_6_-GABA, respectively. HEK293 cell-free blanks, zero samples and GABA standards were prepared in the same way in the absence of HEK293 cells. GABA and D_6_-GABA were analyzed by LC-ESI–MS/MS, recording the mass transitions *m/z* 104.0/87.0, 104.0/69.0, 110.0/93.0 and 110.0/73.0 using the same instrumentation as described for mass spectrometry binding assays. Liquid chromatography was performed with a YMC Pack PVA Sil (5 µm, 50 mm × 2.1 mm) column (YMC-Europe) equipped with two frits (0.5 µm and 0.2 µm, Idex) as a stationary phase in combination with a mobile phase consisting of 5 mM ammonium bicarbonate and acetonitrile (30/70, v/v) at a flow rate of 600 µl min^–1^ and an injection volume of 10 µl (taken directly from the 96-well cell culture plates) according to a recently described method^54^. Quantification of GABA was based on the area ratios obtained for GABA and D_6_-GABA at the mass transitions *m/z* 104/87 and 110/73, respectively. GABA concentrations in HEK293 cell samples were calculated by linear regression according to the standard addition approach using GraphPad Prism (version 6.0.7). GABA concentrations per cell were estimated, assuming a volume of 1.25 pl per HEK293 cell as described earlier^55^.

### Analysis of GAD1 transcription in HEK cells

To identify the source of GABA, we checked for the presence of glutamate decarboxylase in HEK cells through rt–qPCR. Total RNA was extracted from 5 million HEK cells, using TRIzol reagent (Life Technologies) in the standard RNA isolation protocol (Invitrogen). A 1 µg sample of RNA was treated with DNAse followed by cDNA preparation using RNA sample, RevertAid (Thermo Fisher) reverse transcriptase, dNTPs and random hexamer oligonucleotides. Isolated cDNA was used for rt–qPCR using GAD1 primers (Supplementary Table 2) and actin primers as a control. For the no-template control reaction, GAD1 primers were used without cDNA in the reaction mixture. The rt–qPCR was performed in a CFX Opus 96 Real-Time PCR (Biorad) for 35 cycles at 95 °C for 15 s, 57 °C for 30 s and 30 s at 72 °C, followed by one cycle at 72 °C for 4 min in the presence of TB Green (Takara). Each reaction was performed in triplicate and the result was plotted.

### Molecular simulations of GABA bound to GAT1_EM_ and Tyr140Phe GAT1_EM_

All simulation boxes were prepared using CHARMM-GUI server^56^. For both rGAT1_EM_ and its Tyr140Phe mutant, the proteins were aligned using the PPM 2.0 server. A ratio of 5:2:3 of POPC:POPE:cholesterol was used to construct the square lipid bilayer with an edge of ~107 Å. The electrically neutral simulation boxes contained ~127,000 atoms including a 35–36 Å thick TIP3 water model with 150 mM NaCl. The simulations were run on charmm36m force field on a GROMACS v2020 engine. For Try140Phe mutant simulation, tyrosine to phenylalanine mutation was performed in silico and equilibrated likewise^57^.

Energy minimization was performed using the steepest descent algorithm with a maximum force tolerance of 1,000 kJ mol^−1^ nm^−1^. Temperature was set at 310 K using a V-rescale thermostat, and semi-isotropic pressure coupling was set at 1 bar, using a Berendsen thermostat during pressure equilibration; fast smooth particle mesh electrostatics were used throughout. To ensure that the system was properly energy-minimized and equilibrated before starting unrestrained molecular dynamics simulations, separate positional restraints were applied on the sodium ion and the GABA molecule modeled in the cryo-EM structure. Stepwise reductions in positional restraints for the protein and lipid bilayer were applied during two temperature and four pressure equilibration steps, while a constant positional restraint of 4,000 kJ mol^−1^ nm^−1^ was applied to GABA and modeled sodium during the first four equilibration steps. We carried out stepwise removal of the positional restraints from GABA and the sodium ion in five successive equilibration steps of 250 ps each. Molecular dynamics simulations were run with at least four different random seeds for each of the two systems for 100 ns each with a Parrinello-Rahman barostat.

For root mean squared deviation (r.m.s.d.) and distance calculations, rGAT in the trajectories were aligned to provide a pseudo-constant frame of reference for the position of GABA in the binding site.

### Model generation

The AlphaFold2 model of rGAT1_WT_ in the outward-open state was obtained in-house using ColabFold^58^ (version 1.5.2), as the EBI AlphaFold database entry for hGAT1 (AF-P30531) provided only an outward-occluded state. The MMSeq2 method was used to generate the multiple sequence alignment, and Amber relaxation was not used on the generated model.

### Reporting summary

Further information on research design is available in the Nature Portfolio Reporting Summary linked to this article.

## Online content

Any methods, additional references, Nature Portfolio reporting summaries, source data, extended data, supplementary information, acknowledgements, peer review information, details of author contributions and competing interests, statements of data and code availability are available at 10.1038/s41594-023-01011-w.

## Supplementary information

Supplementary Information Supplementary Tables 1 and 2, and Supplementary Figs. 1–8.

Reporting Summary

Peer Review File

Supplementary Data 1 Source Data Extended Data Fig. 4 contains a representative cryo-EM micrograph image, FSC curve image, rGAT1_EM_ coordinates file and the rGAT1_WT_ AlphaFold model file.

## Data Availability

The refined coordinates and maps of the rGAT1 structure in this study have been deposited in the PDB (PDB 8GNK) and EMDB (EMD 34167). The raw data for the experiments have been deposited along with the manuscript as a source data file and raw chromatograms for LC-ESI–MS/MS data are provided in Supplementary files. Molecular dynamics trajectories have been deposited at https://osf.io/f9nr6/?view_only=a859d5ff464c404196eedb935373b0e3. Source data are provided with this paper.

## Source data

Source Data Fig. 1 Fig1_data has four Excel sheets. Fig1c_data contains chromatogram data dDAT, rGAT1_WT_ and rGAT1_EM_. Fig1e_data contains raw data values for GABA uptake in rGAT1_WT_ and rGAT1_EM_ constructs. Fig1f_data contains raw data values for uptake inhibition by tiagabine and NO711 for rGAT1_EM_. Fig1g_data contains MS binding data of NO711 with rGAT1_EM_.
Source Data Extended Data Fig. 1 Fig_ED1_data contains FSEC chromatogram data for dDAT, rGAT1_WT_, rGAT1_epi1_, rGAT1_epi2_, rGAT1_epi3_, rGAT1_epi4_, rGAT1_epi5_, rGAT1_epi6_, rGAT1_epi7_, rGAT1_epi8_ and rGAT1_epi9_.
Source Data Extended Data Fig. 2 Fig_ED2 has four Excel sheets. Fig_ED2a_data contains FSEC chromatogram data for rGAT1_WT_, rGAT1_epi4_ and rGAT1_EM_. Fig_ED2b_data contains raw data values for uptake inhibition by tiagabine and NO711 for rGAT1_WT_. Fig_ED2c_data contains MS binding data of NO711 with rGAT1_WT_. Fig_ED2d_data contains raw data values for GABA uptake in rGAT1_WT_ and rGAT1_EM_ constructs.
Source Data Extended Data Fig. 3 Fig_ED3 has two Excel sheets. Fig_ED3a_data contains SEC chromatogram data of purified rGAT1_EM_. Fig_ED3b_data contains SEC chromatogram data of purified 9D5.
Source Data Extended Data Fig. 3 Fig_ED3_gel_image has two images. Fig_ED3a contains a gel image of purified rGAT1_EM_. Fig_ED3b_data contains a gel image of purified 9D5.
Source Data Extended Data Fig. 7 Fig_ED7 has two Excel sheets. Fig ED_7g_data contains MS/MS data for GABA quantification. Fig ED_7i_qRT_PCR contains GAD1 qRT PCR data including amplification curves and melt curves.
Source Data Extended Data Fig. 9 Fig ED9_data contains rGAT1_EM_ and Tyr140Phe mutant simulation data including r.m.s.d. values and distance values from Tyr140/Phe140.
