# Supplementary material for: Cryo-EM structure of GABA transporter 1 reveals substrate recognition and transport mechanism
Source: Nat Struct Mol Biol. 2023 Jul 3;30(7):1023–32. doi: 10.1038/s41594-023-01011-w (PMC10352132; doi:10.1038/s41594-023-01011-w)
Supplement: Supplementary file 1 — Supplementary Tables 1 and 2, and Supplementary Figs. 1–8. [file 41594_2023_1011_MOESM1_ESM.pdf]

nature structural & molecular biology

Article

https://doi.org/10.1038/s41594-023-01011-w

Cryo-EM structure of GABA transporter 1 reveals substrate recognition and transport mechanism

In the format provided by the authors and unedited

Table of contents – Supplementary figures and tables:

Supplementary Tables

Table S1 - Transport kinetics and binding affinity of rGAT1 constructs…………….......….2

Table S2 - List of primers used in the study…………………………………………………...3

Supplementary Figures

Fig. S1 - Alignment of SLC6 members related to $rGAT1_{WT}$ and $rGAT1_{EM}$………….….….…4

Fig. S2 - Na+, L-alanine, β-alanine, GABA, Tris, Acetate, Betaine positioned into the density next to the bound GABA molecule………………………………………......5

Fig. S3 - Structural comparison of rGAT1 with SLC6 members……………………….…....6

Fig. S4 - ^{3}[H]-GABA uptake and Inhibitor binding assay replicates for $rGAT1_{WT}$…………7

Fig. S5 - ^{3}[H]-GABA uptake and Inhibitor binding assay replicates for $rGAT1_{EM}$……......8

Fig. S6 – Raw chromatograms of MS based binding assay of NO711 with $rGAT1_{EM}$……9

Fig. S7 – Raw chromatograms of MS based binding assay of NO711 with $rGAT1_{WT}$…..17

Fig. S8 – Raw chromatograms of GABA detection in HEK293 cells……………………..25

Table S1:

Transport kinetics and binding potency of rGAT1 constructs

a. GABA Uptake by $rGAT1_{WT}$, $rGAT1_{Epi4}$ and $rGAT1_{EM}$

	K_M (µM) (mean ± SEM)	V_{MAX} (f.moles/well/min) (mean ± SEM)
**$rGAT1_{WT}$**	10.95 ± 1.47	2023 ± 113.1
**$rGAT1_{EM}$**	4.16 ± 0.5	855.7 ± 33.8
**$rGAT1_{Epi4}$**	3.22 ± 0.49	632 ± 27.3

b. IC_{50} values of GAT1 inhibitors

	$rGAT1_{WT}$ (mean ± SEM)		$rGAT1_{EM}$ (mean ± SEM)	
	IC_{50} (nM)	K_i (nM)	IC_{50} (nM)	K_i (nM)
**NO711**	563 ± 61	325 ± 35	154.9 ± 11 nM	53 ± 4
**Tiagabine**	918 ± 83	530 ± 48	704 ± 95 nM	241 ± 32

c. Binding affinity for NO711 measured from MS binding assays

	K_D (mean ± SEM) (nM)	B_{MAX} (mean ± SEM) (pmol /mg protein)
**$rGAT1_{WT}$**	50.6 ± 6.3	166.4 ± 44.6
**$rGAT1_{EM}$**	80.7 ± 10.0	212.4 ± 30.3

Table S2:

List of Primers

Sl. No.	Primer name	Sequence (5' ⟶ 3')	Purpose
1	rGAT1_EcoRI_FP	GCGCGGAATTCCCACCATGGCGACTGACAACAGCAAGGTGG	GAT1 cloning
2	rGAT1_notI_RP	GCGGCCGCAGAGCCGCGCGGCACCAGTCCTGCGGTACCGCATGCC	GAT1 cloning
3	del38_rGAT1_FP	GCGCGCGGAATTCCCACCATGGGAGACCTCCCTGACCGGGAC	N-terminal 37 aa deletion primer
4	rGAT1_epi1_FP	AACCGGTTCTCTGAGGACATCCGGGACATGGTTGGC	epi1 epitope mutant primer
5	rGAT1_epi2_FP	ACATCCGGGACATGATTGGCTTCCCACCCTGCATCTGGTGG	epi2 epitope mutant primer
6	F312Y_FP	GCTACAACTCTTACCACAACAATGTGTACAG	epi4 epitope mutant primer
7	E575R_RP	CAGGGCGCACGATATCACGACTGGGCTGAAT	epi3 epitope mutant primer
8	Y107H_FP	CTCCCTAGGCCAGCACACCTCCATTGGG	epi5 epitope mutant primer
9	GAD1_FP	TGTCCAGGAAGCACCGCCATAA	GAD1_qRT_PCR
10	GAD1_RP	TCCTTGACGAGAATGGCAGAGC	GAD1_qRT_PCR

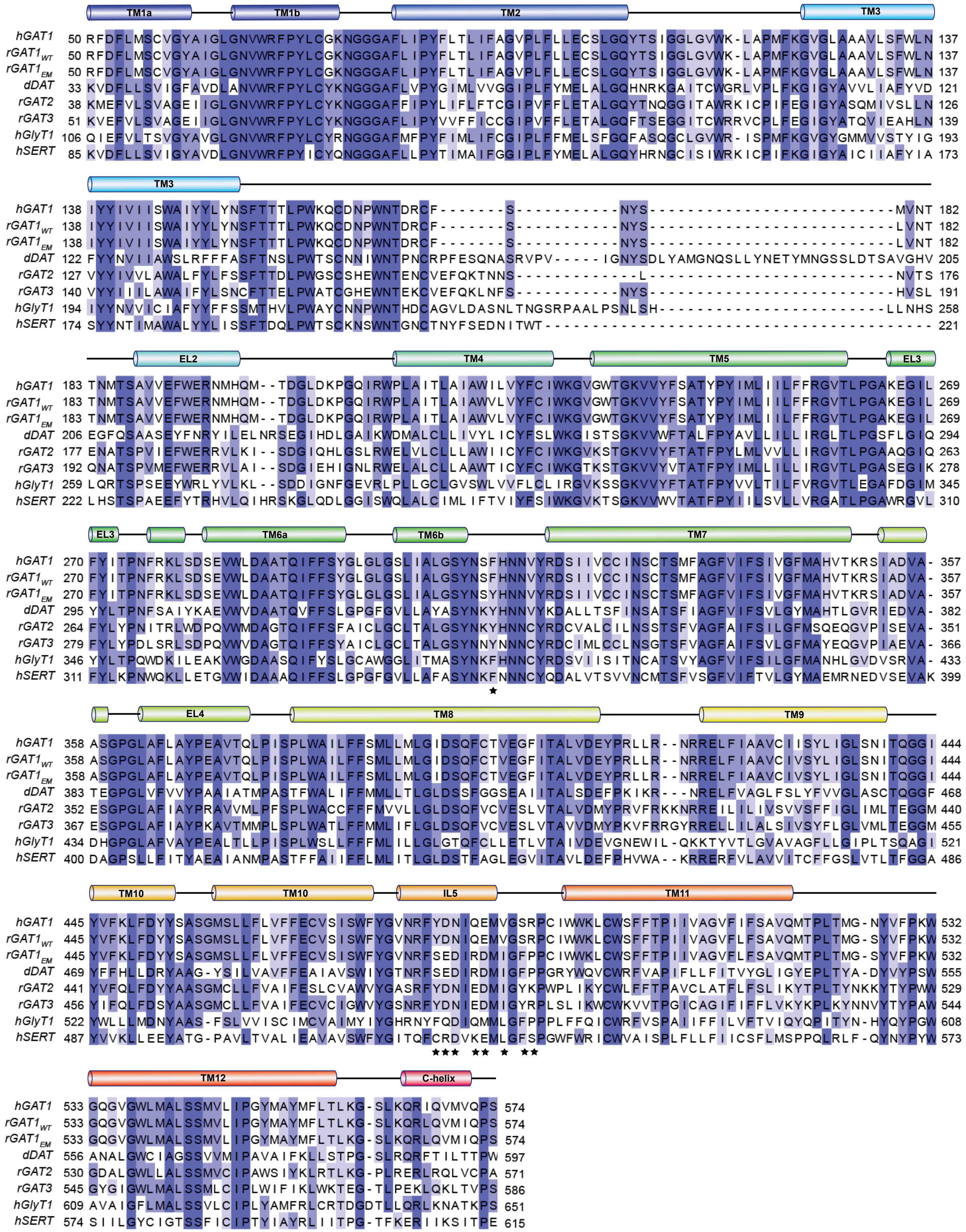

Fig. S1 – Alignment of SLC6 members related to rGAT1WT and rGAT1EM

Multiple sequence alignment of NSS transporters including rGAT1WT, hGAT1, dDAT, rGAT2, rGAT3, hGlyT1 and hSERT with rGAT1EM, displaying the position of TM helices and loops. The epitope mutations corresponding to in rGAT1EM are labeled with asterisks.

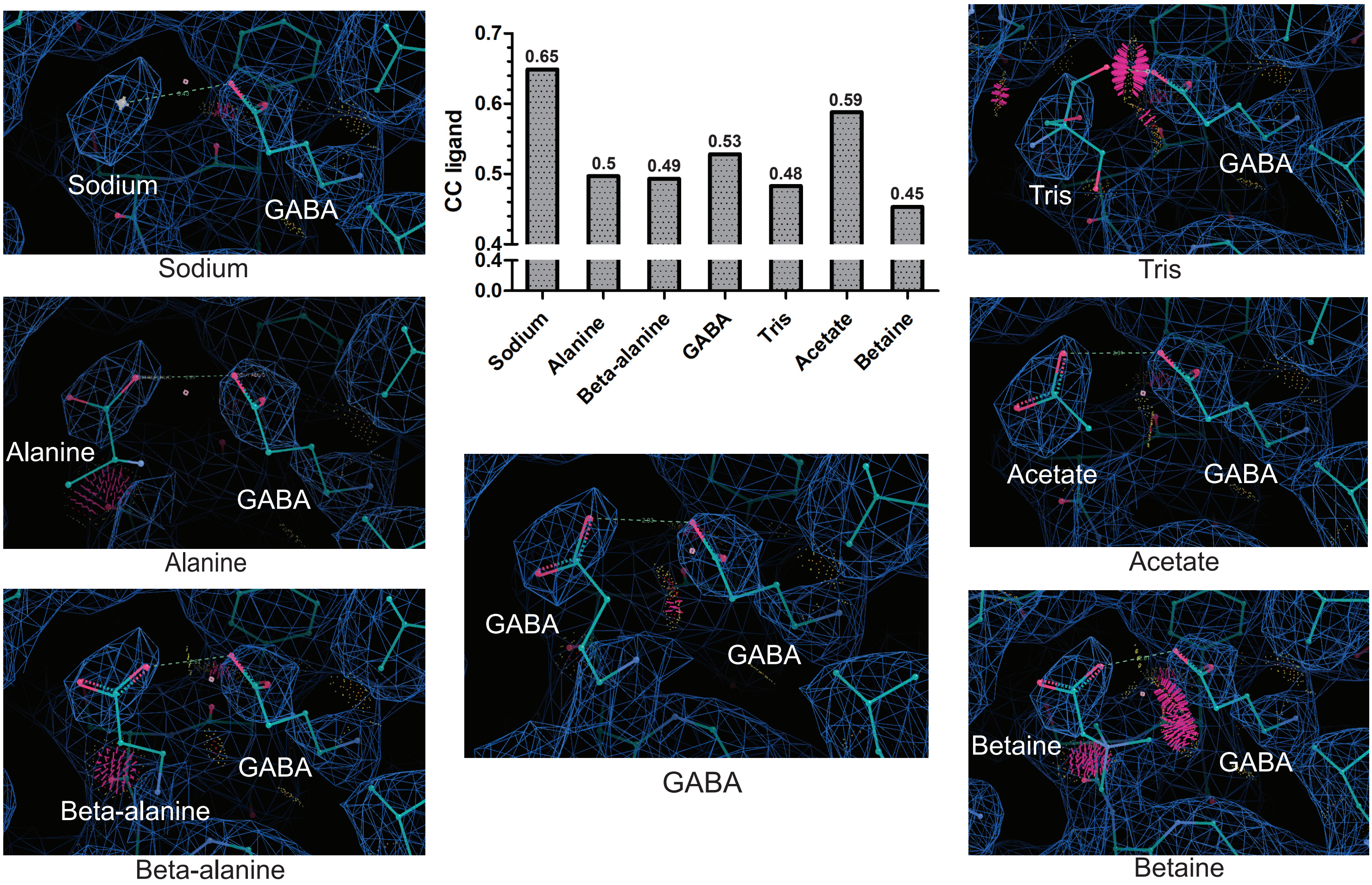

Fig. S2 – Ligand fitting to the density near bound

Sodium ion (Na^+), L-alanine, β-alanine, GABA, Tris base, acetate and betaine positioned into the density next to the bound GABA molecule in coot and fitted through real space refinement. The coot images display sodium buried inside the density while other ligands have protrusions outside the density. The fitted coordinates along with the density map were validated in PHENIX comprehensive cryo-EM validation tool. The histogram displays the comparison of CC values for all ligands displaying highest CC score for sodium ion (cc=0.65) compared to other ligands (range- 0.45 to 0.59).

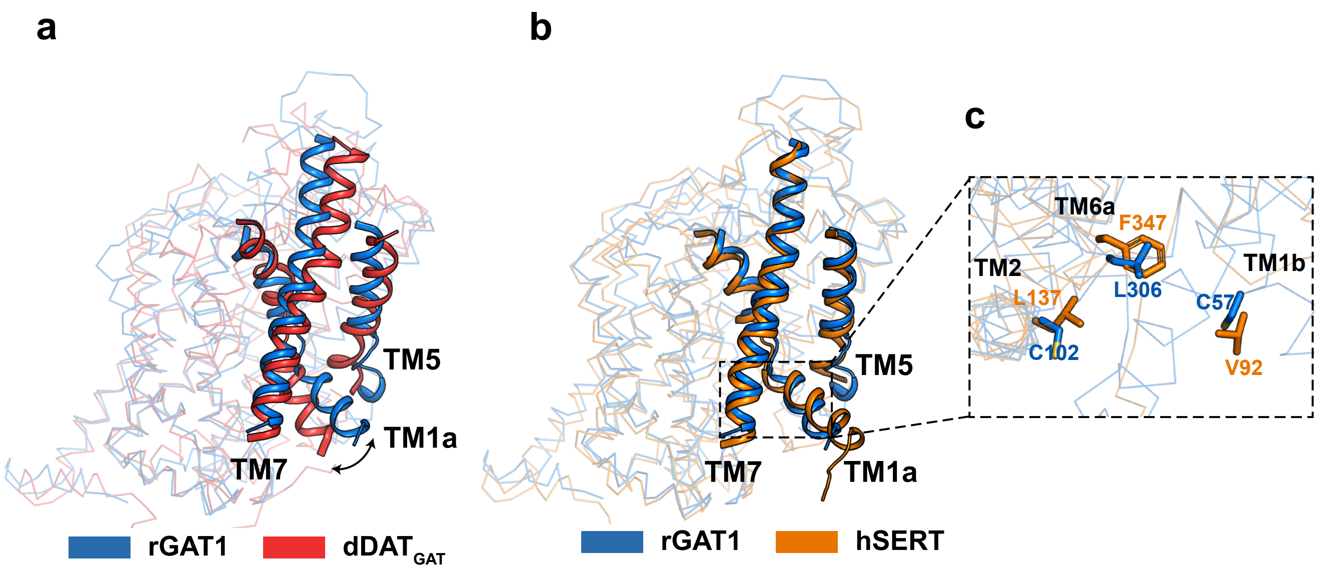

Fig. S3 – Structural comparison of rGAT1 with SLC6 members. Structural alignment of TM1a, TM5 and TM7 of rGAT1$_{EM}$ with **a**, outward-open structure of dDAT$_{GAT}$ (PDB id 7WGT; Cα rmsd – 2.7 Å) and **b**, inward-open structure of hSERT (PDB id 6DZZ; Cα rmsd–1.3 Å**). c,** Panel represents differences in residues lining the cytosolic pathway among hSERT and rGAT1 primarily in hydrophobic residues.

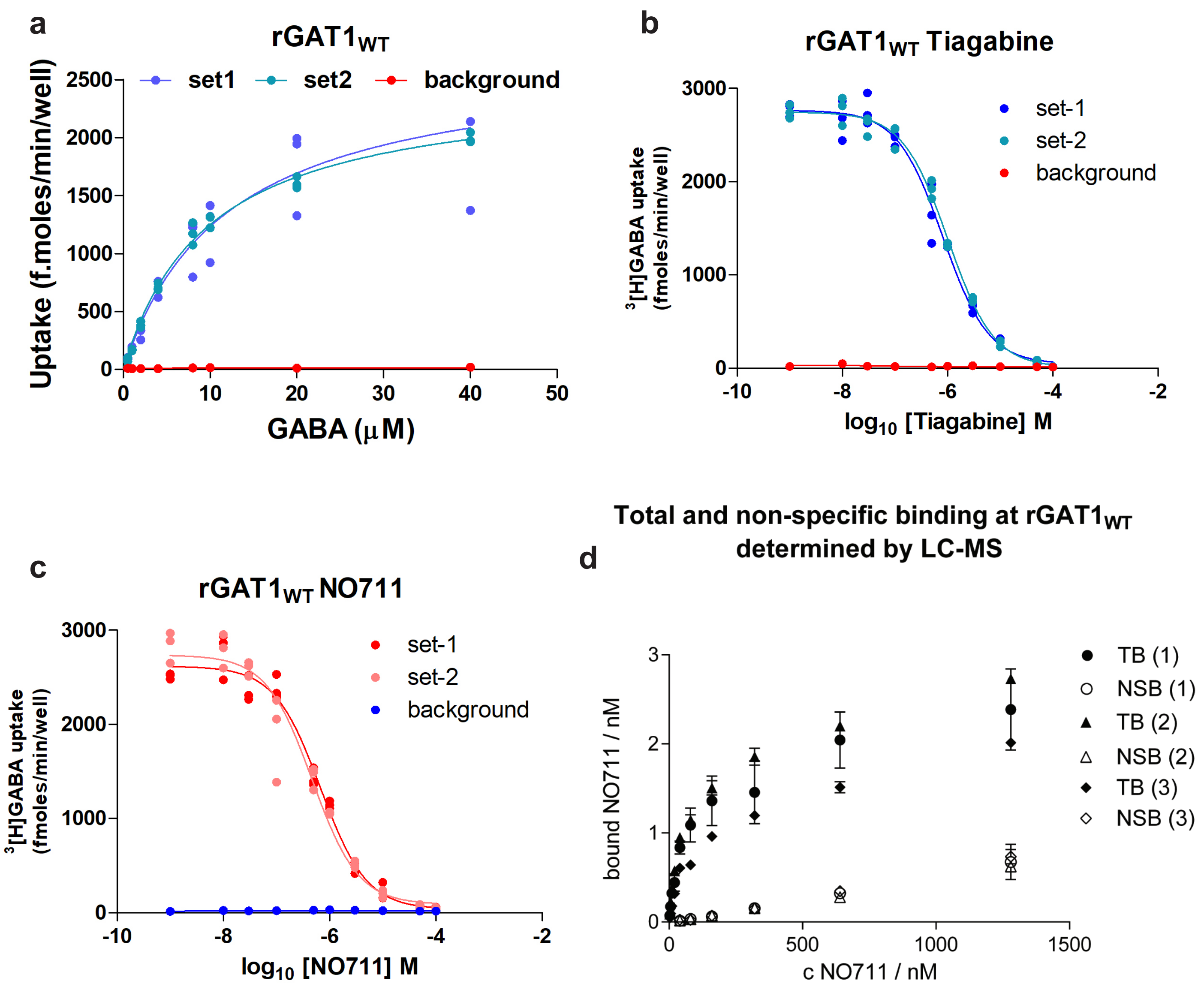

Fig. S4 – ^{3}[H]-GABA uptake and Inhibitor binding assay replicates for rGAT1$_{WT}$

a, ^{3}[H]-GABA uptake of rGAT1$_{WT}$ displaying two independent experiments as separate lines (blue and teal) and individual data points for total counts as dots. Background counts are displayed in red. **b,** Inhibition of ^{3}[H]-GABA uptake by rGAT1$_{WT}$ by tiagabine displaying independent trials as separate lines (blue and teal) and individual data points as dots. Background counts are displayed in red. **c,** Inhibition of ^{3}[H]-GABA uptake by rGAT1$_{WT}$ by NO711 displaying independent replicates of total counts as separate lines (red and salmon) and individual data points as dots. Background counts are displayed in blue. **d,** MS-based binding measurement of the inhibitor NO711 to rGAT1$_{WT}$ expressing membranes. Total (TB) and non-specific binding (NSB) of NO711 to rGAT1$_{WT}$ expressing membranes in three independent experiments shown as means (of triplicates), with SD measured by LC-MS.

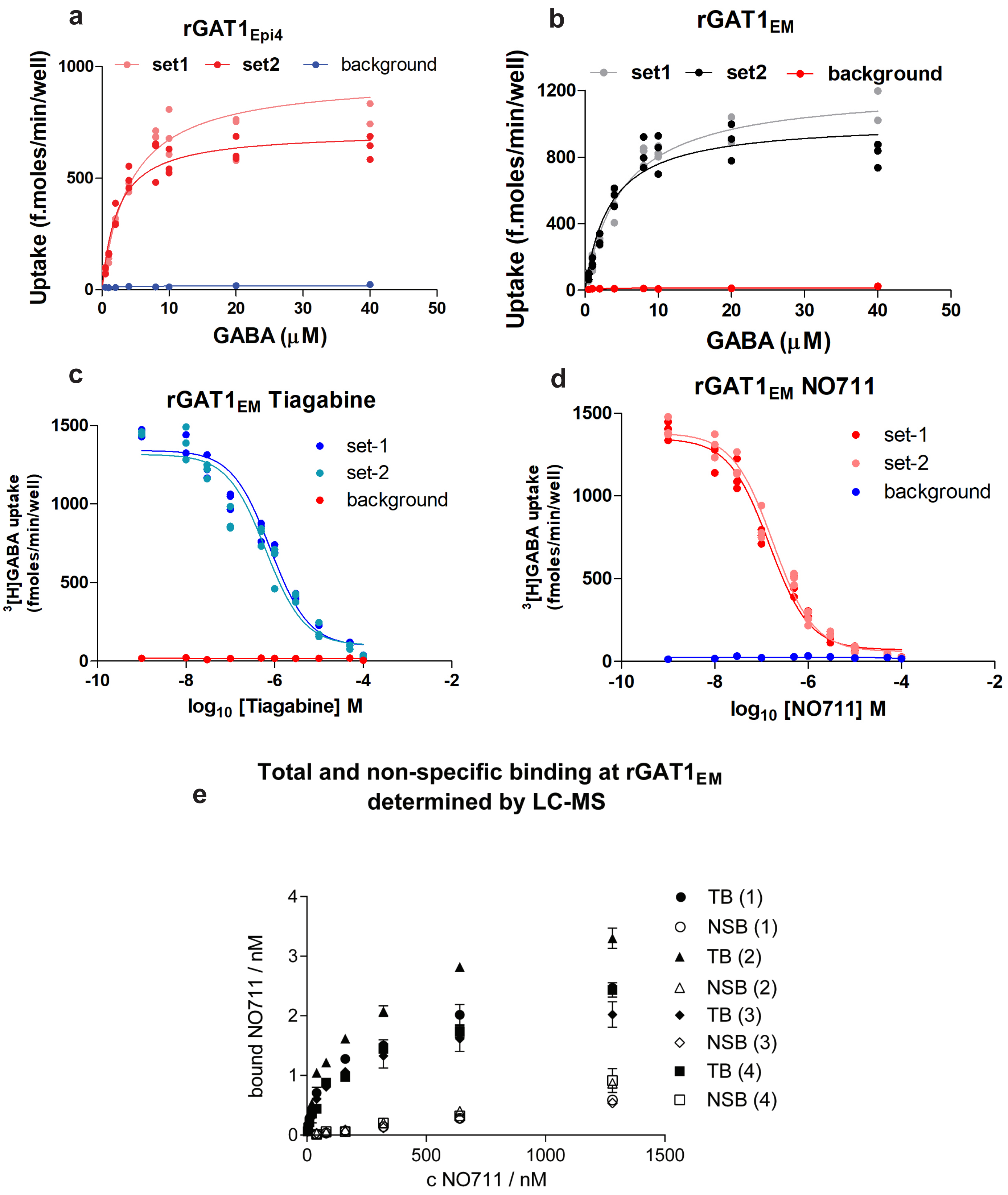

Fig. S5 – ^{3}[H]-GABA uptake and Inhibitor binding assay replicates for rGAT1$_{EM}$

a, ^{3}[H]-GABA uptake by rGAT1$_{Epi4}$ displaying independent replicates as separate lines (red and salmon) and individual data points for total counts as dots. Background counts are displayed in blue. **b,** ^{3}[H]-GABA uptake by rGAT1$_{EM}$ displaying independent replicates as separate lines (black and grey) and individual data points as dots. Background counts are displayed in red. **c,** Inhibition of ^{3}[H]-GABA uptake through rGAT1$_{EM}$ by tiagabine displaying independent replicates of total counts as separate lines (blue and teal) and individual data points as dots. Background counts are displayed in red. **d,** Inhibition of ^{3}[H]-GABA uptake through rGAT1$_{EM}$ by NO711 displaying independent replicates as separate lines (red and salmon) and individual data points as dots. Background counts are displayed in blue. **e,** MS-based binding measurement of inhibitor NO711 to rGAT1$_{EM}$ expressing membranes. Total (TB) and non-specific binding (NSB) of NO711 to rGAT1$_{EM}$ expressing membranes in four independent experiments shown as means (of triplicates), with SD measured by LC-MS.

Fig. S6 - Raw chromatograms of MS based binding assays for $rGAT1_{EM}$ construct.

The mass transitions m/z 351/180 (blue) and 361/190 (red), respectively for NO711 and D10-NO711 were recorded. Chromatograms for the following samples (i.e. concentration levels) are depicted: total binding **a**, 2.5 nM, **b**, 5 nM, **c**, 10 nM, **d**, 20 nM, **e**, 40 nM, **g**, 80 nM, **i**, 160 nM, **k**, 320 nM, **m,** 640 nM, **o**,1280 nM, non-specific binding **f**, 40 nM, **h**, 80 nM, **j**, 160 nM, **l**, 320 nM, **n**, 640 nM, **p**, 1280 nM.

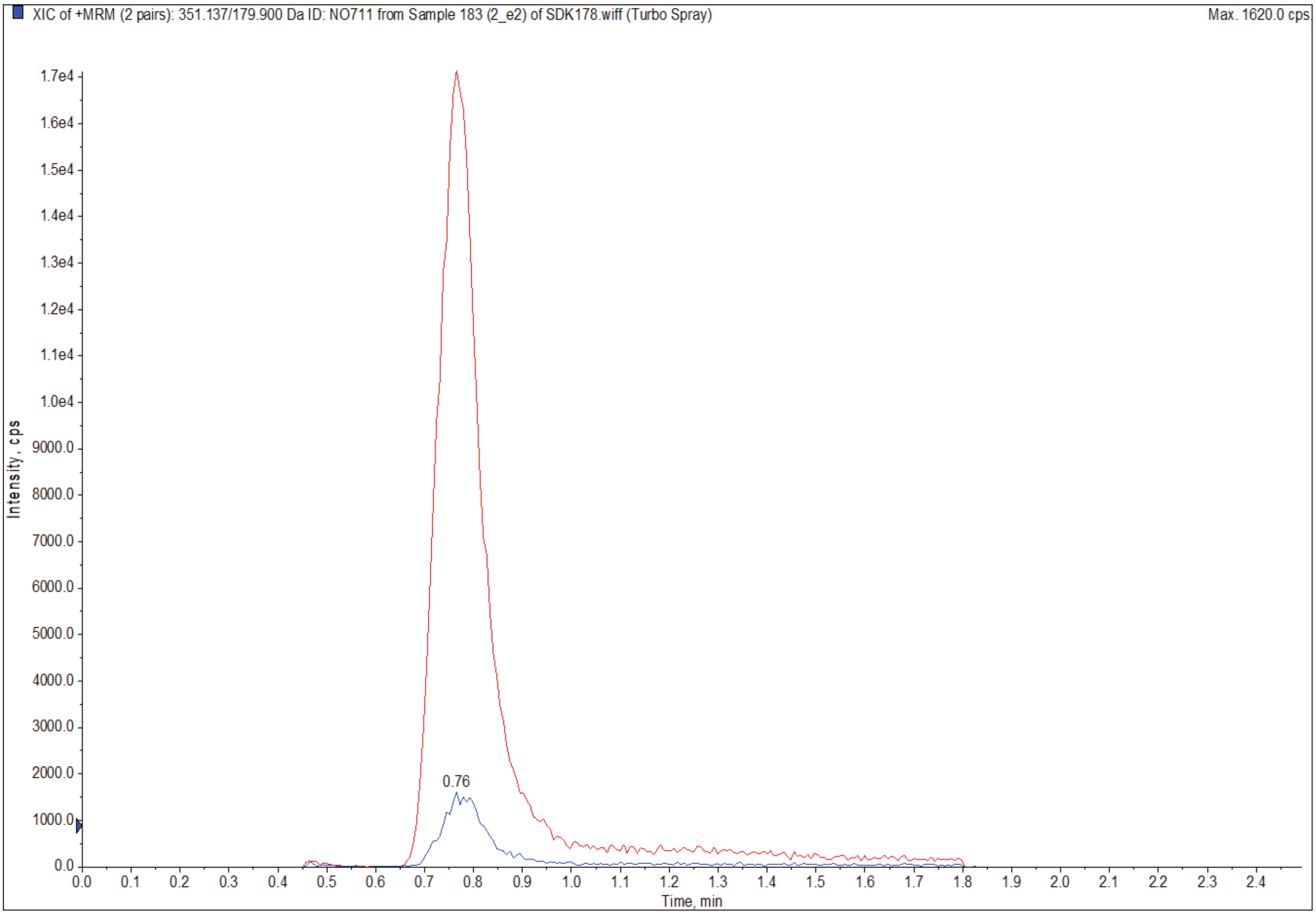

Fig S6a rGAT1$_{EM}$ total binding at 2.5 nM NO711

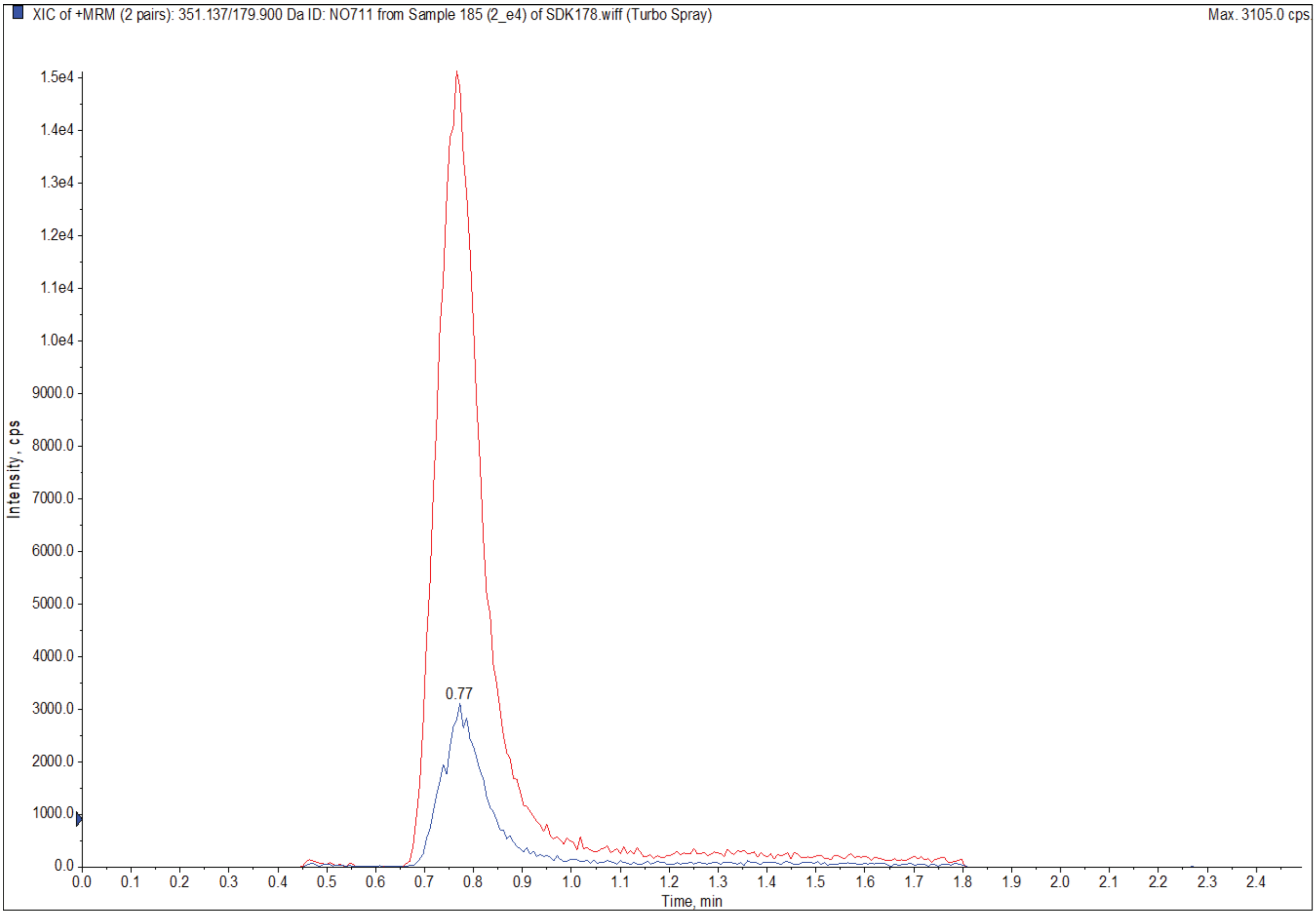

Fig S6b rGAT1$_{EM}$ total binding at 5 nM NO711

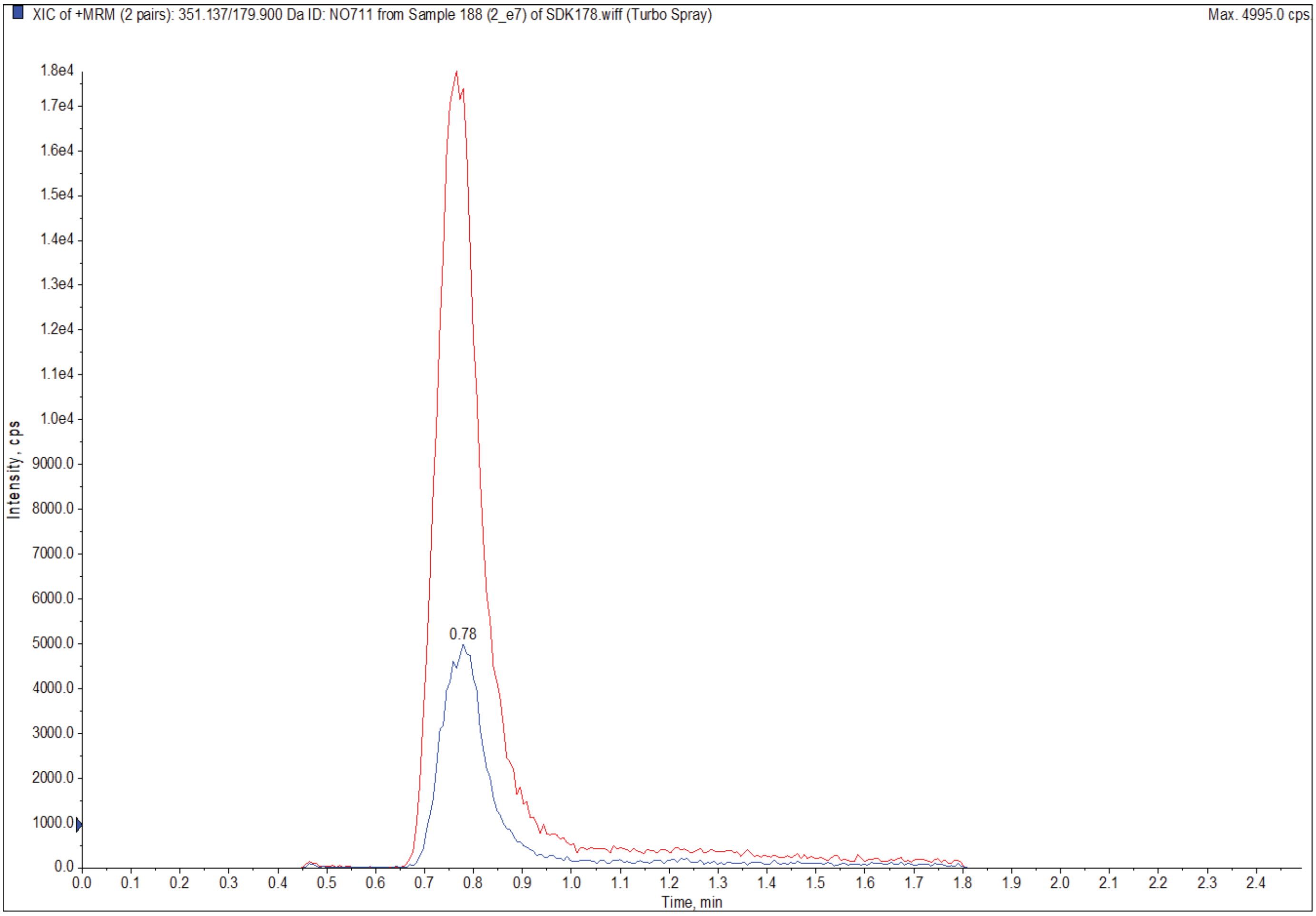

Fig S6c rGAT1$_{EM}$ total binding at 10 nM NO711

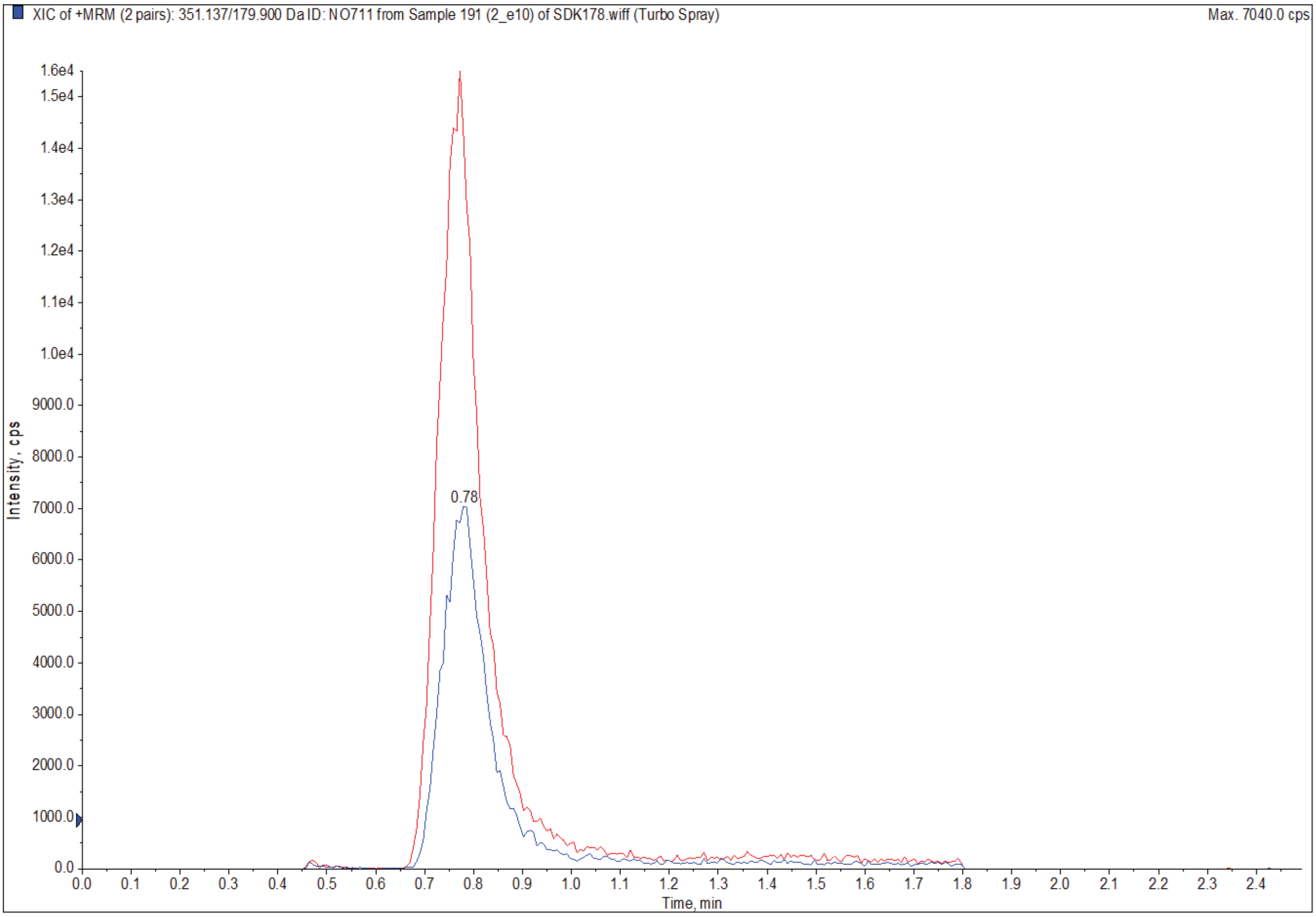

Fig S6d rGAT1$_{EM}$ total binding at 20 nM NO711

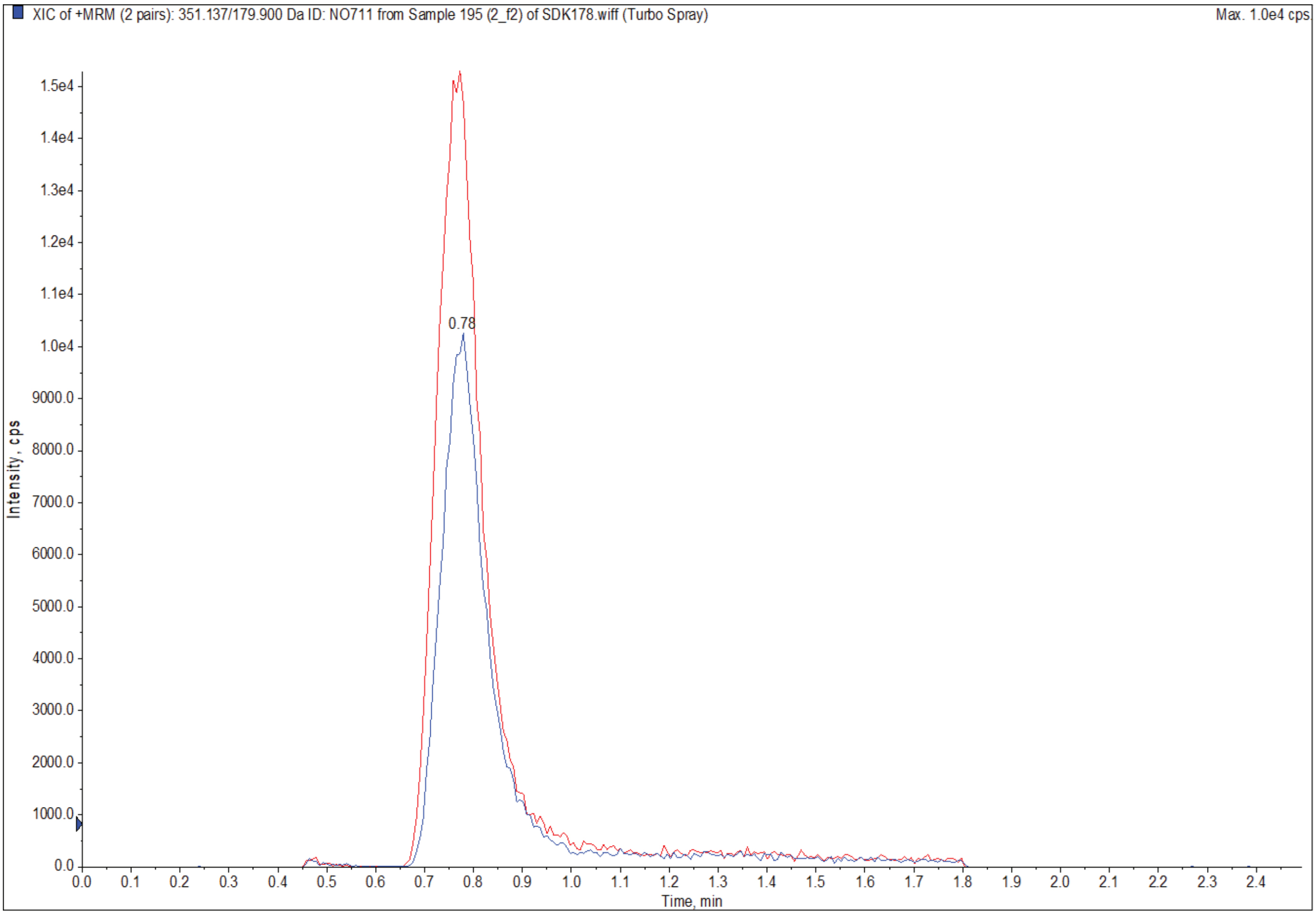

Fig S6e rGAT1$_{EM}$ total binding at 40 nM NO711

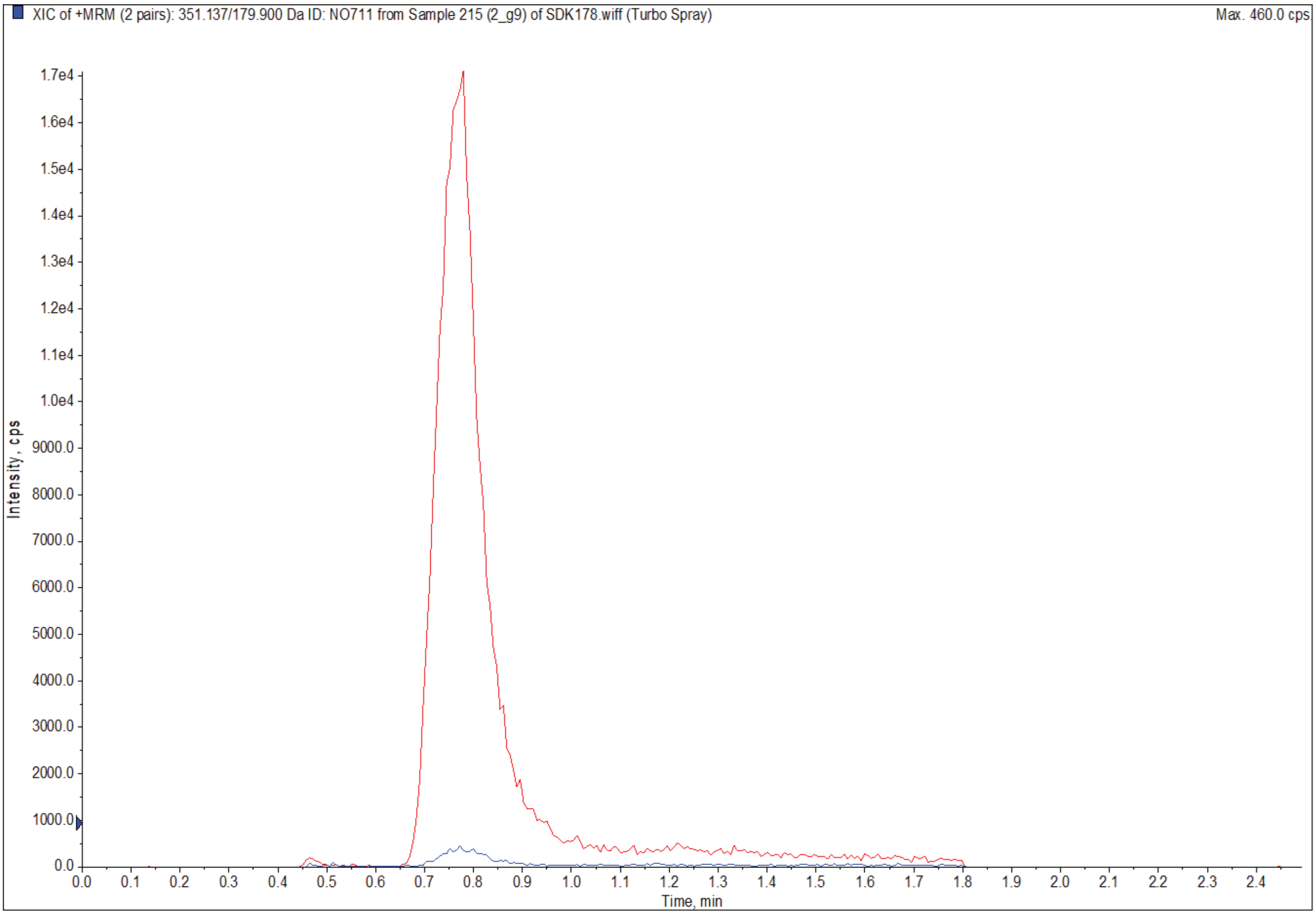

Fig S6f rGAT1$_{EM}$ non-specific binding at 40 nM NO711

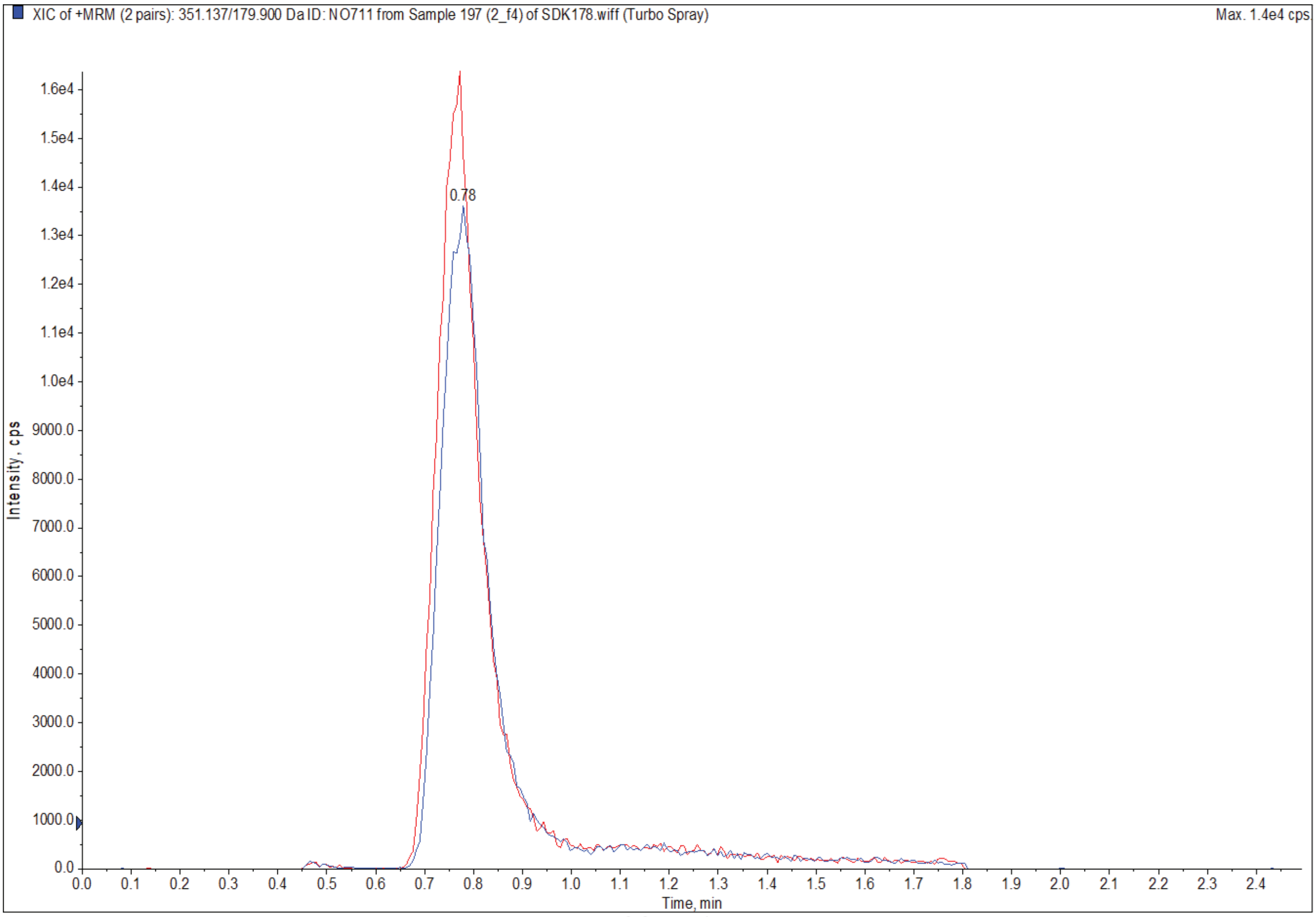

Fig S6g rGAT1$_{EM}$ total binding at 80 nM NO711

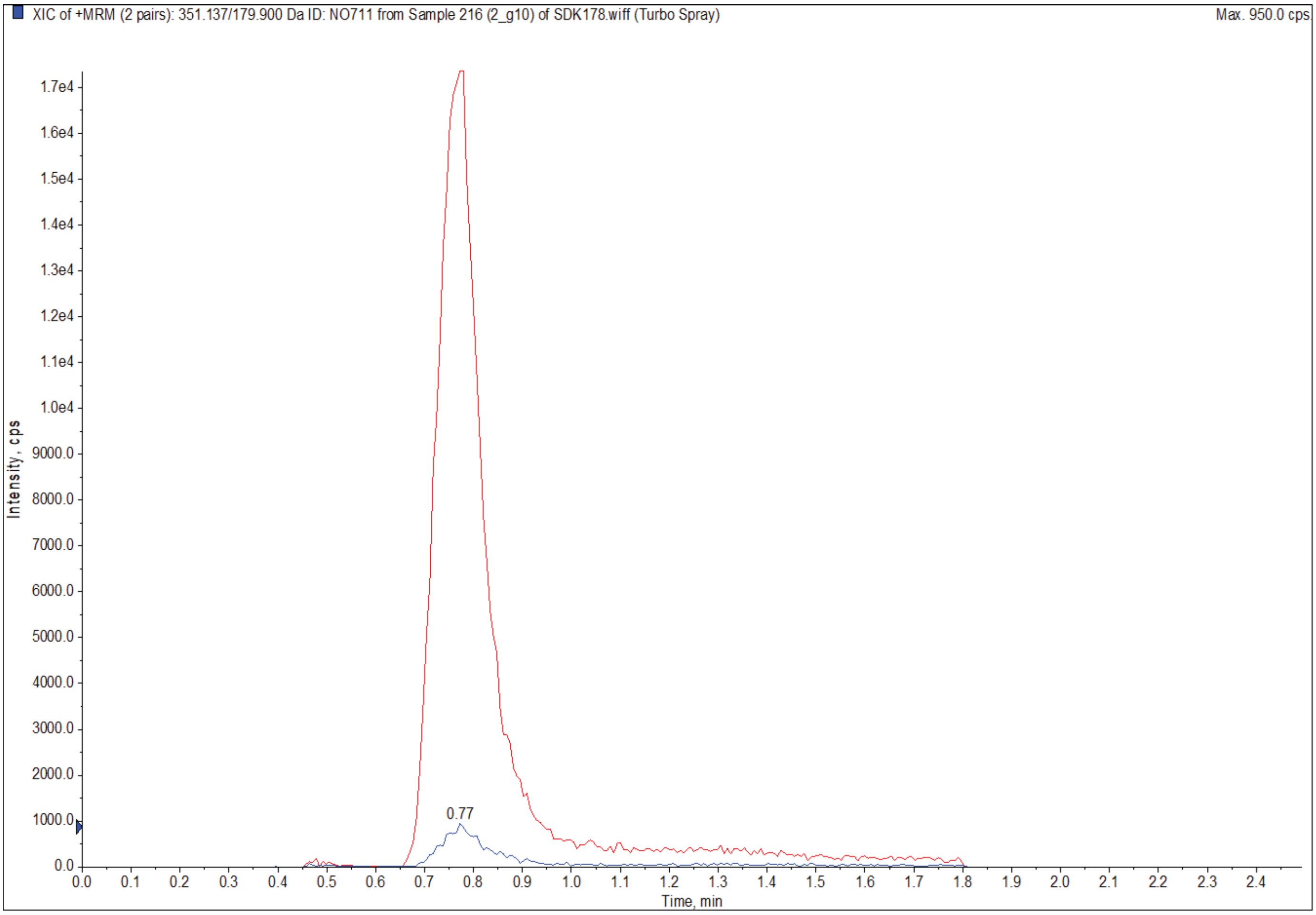

Fig S6h rGAT1$_{EM}$ non-specific binding at 80 nM NO711

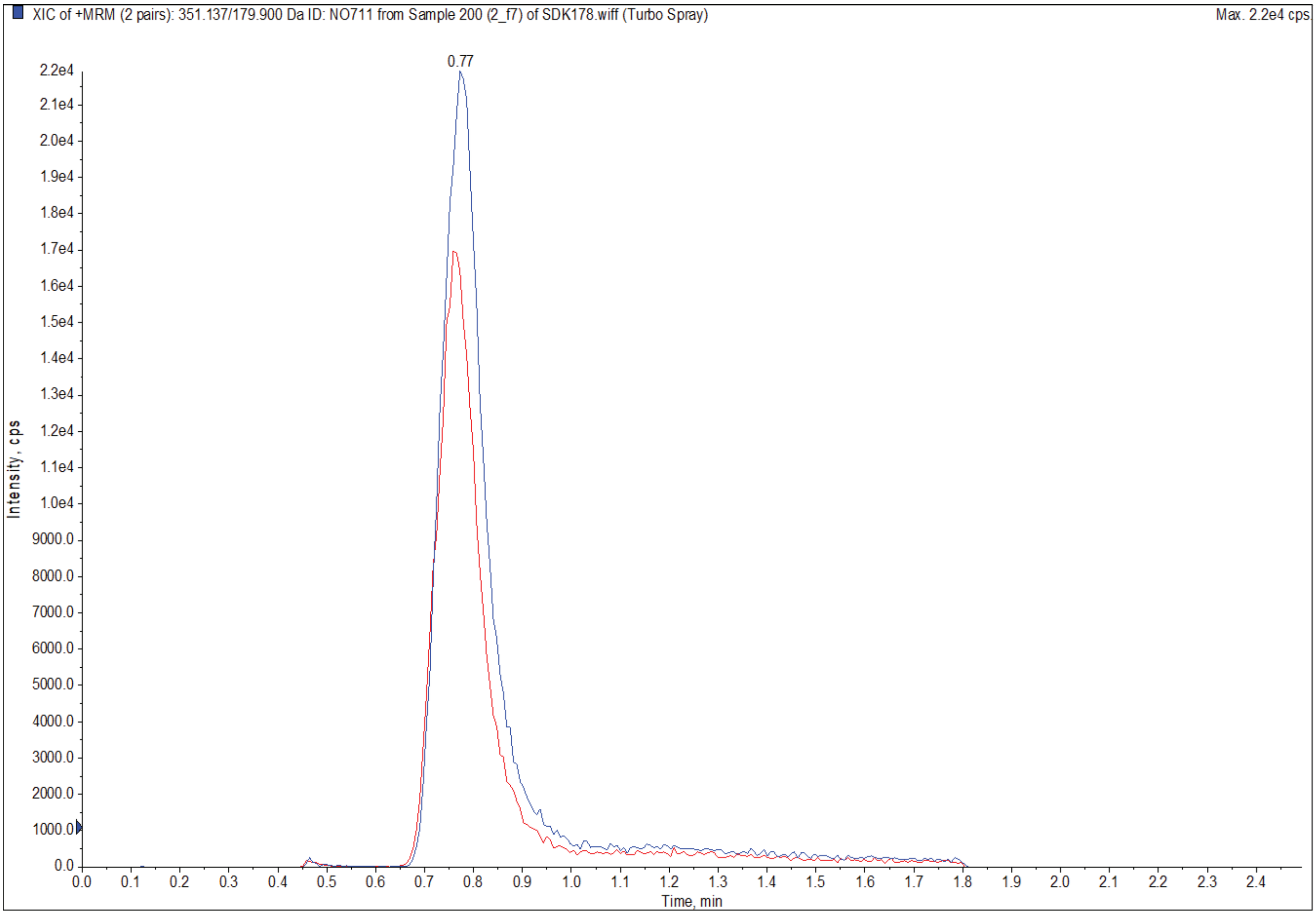

Fig S6i rGAT1$_{EM}$ total binding at 160 nM NO711

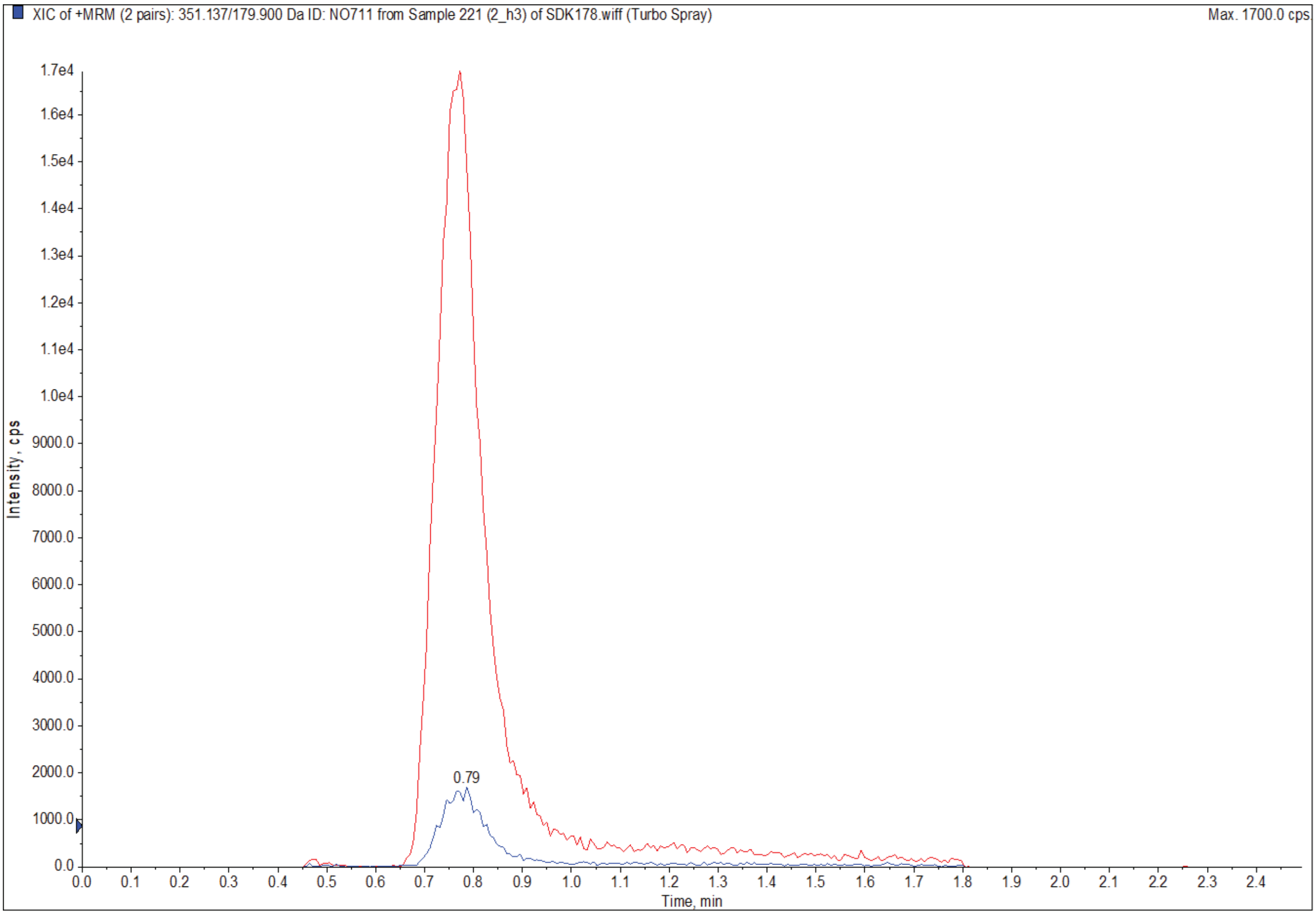

Fig S6j rGAT1$_{EM}$ non-specific binding at 160 nM NO711

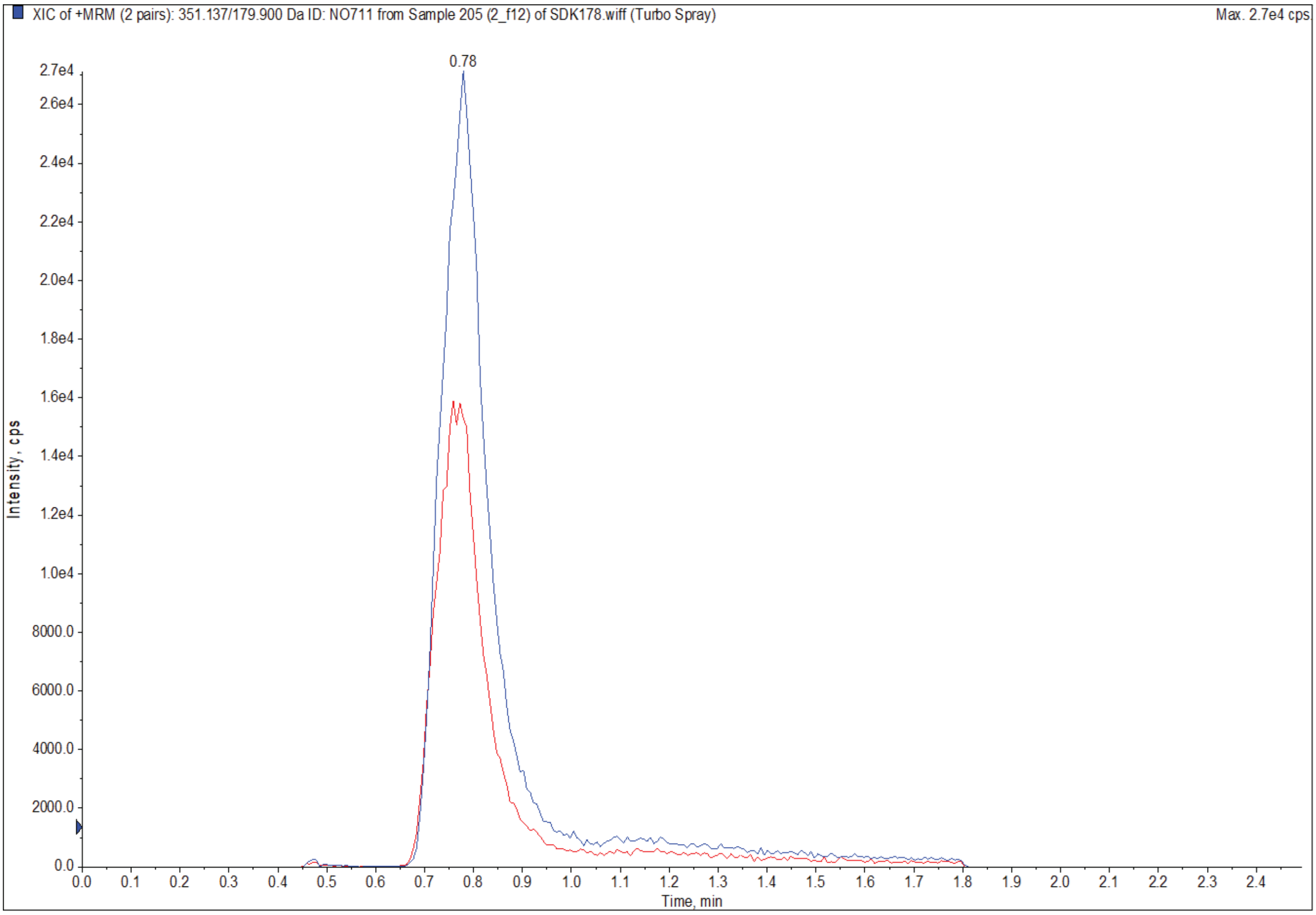

Fig S6k rGAT1$_{EM}$ total binding at 320 nM NO711

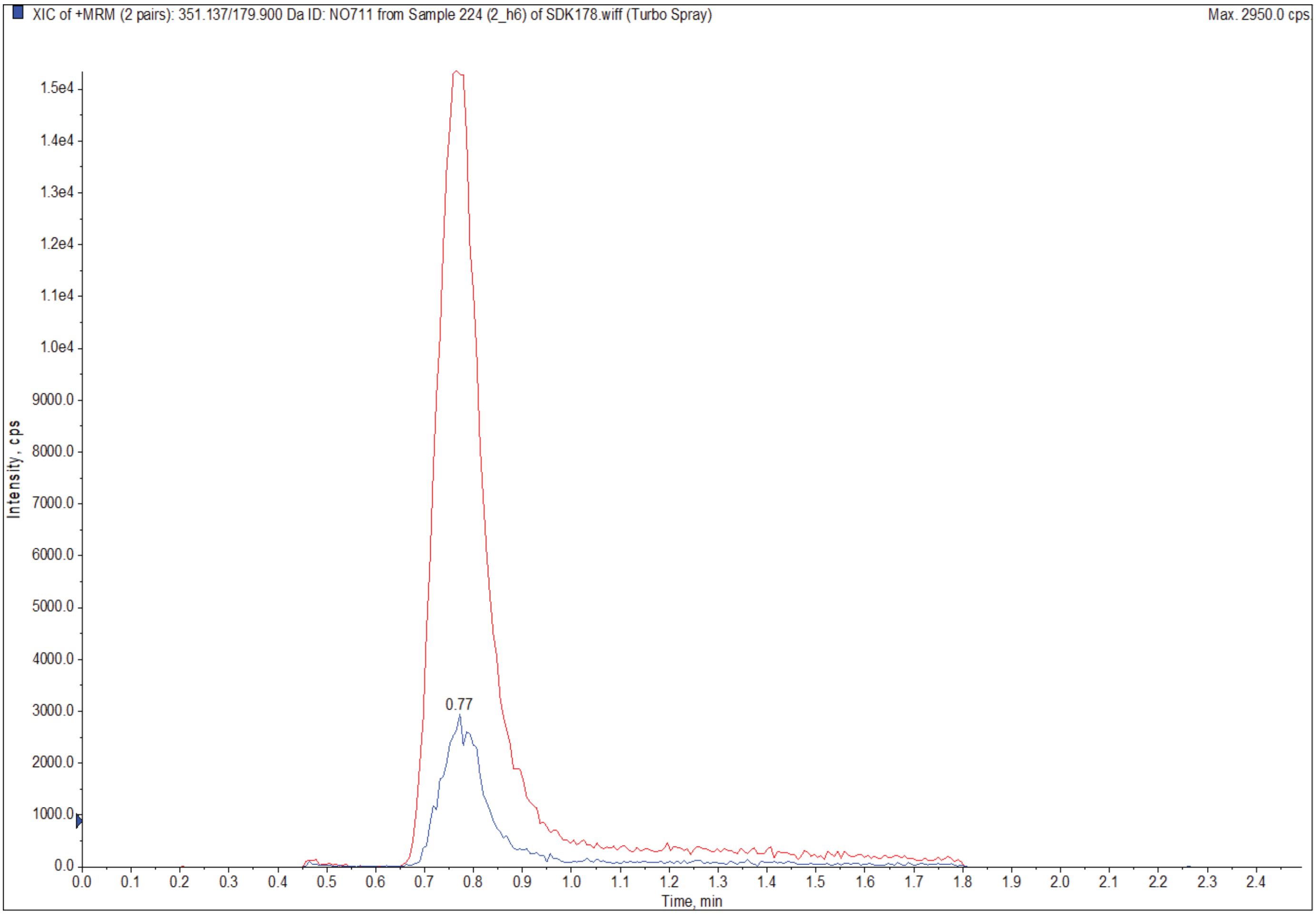

Fig S6l rGAT1$_{EM}$ non-specific binding at 320 nM NO711

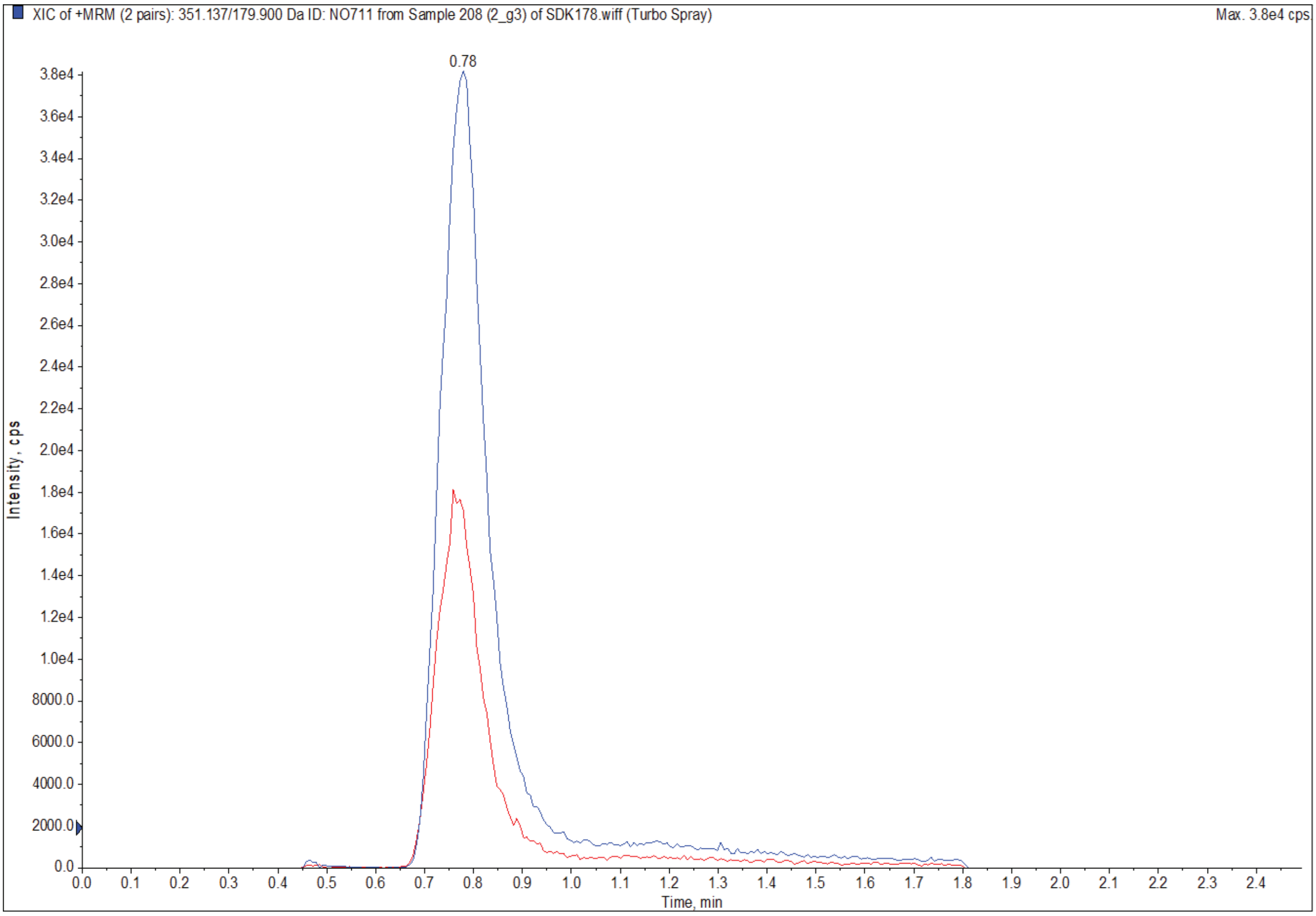

Fig S6m rGAT1$_{EM}$ total binding at 640 nM NO711

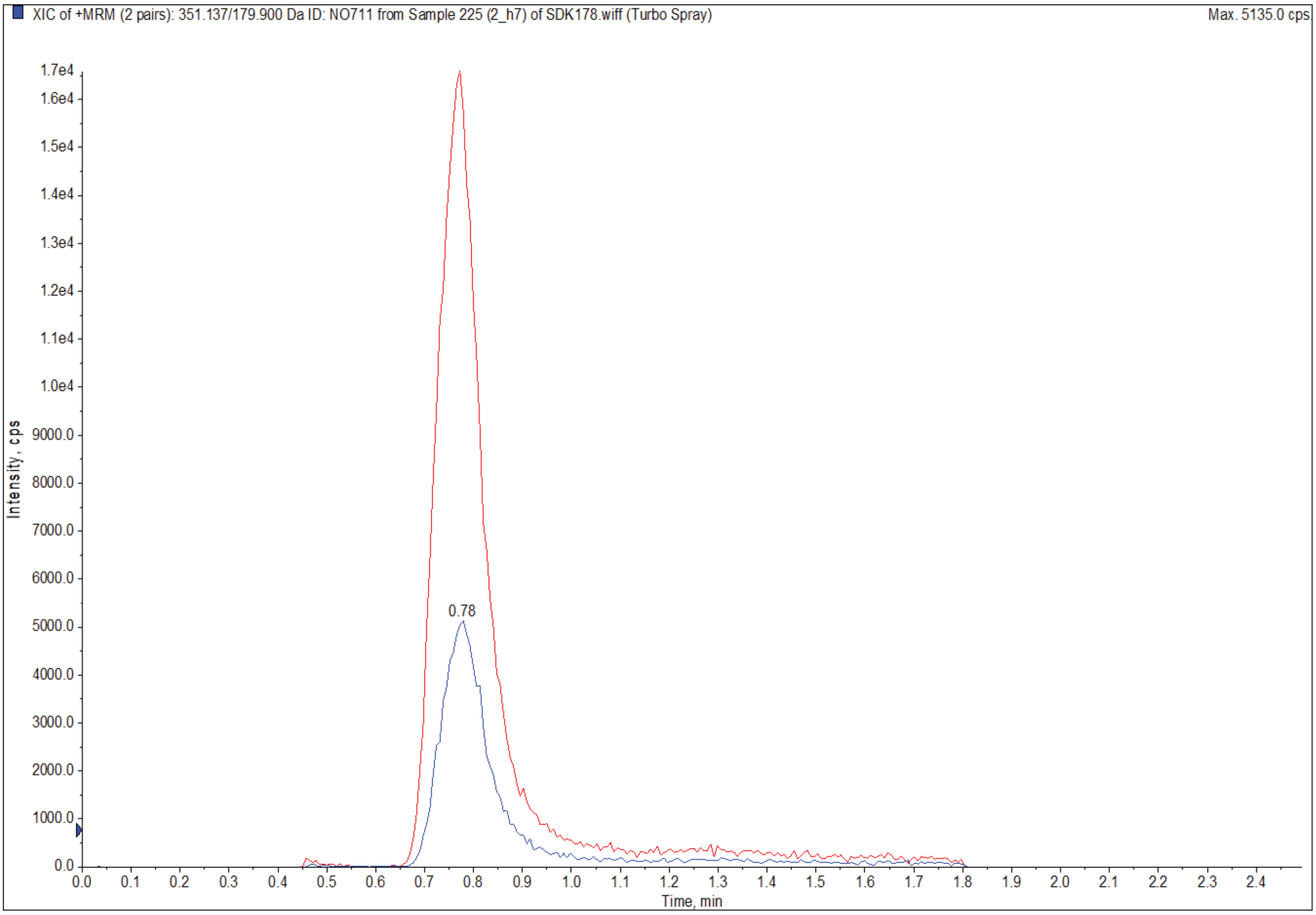

Fig S6n rGAT1$_{EM}$ non-specific binding at 640 nM NO711

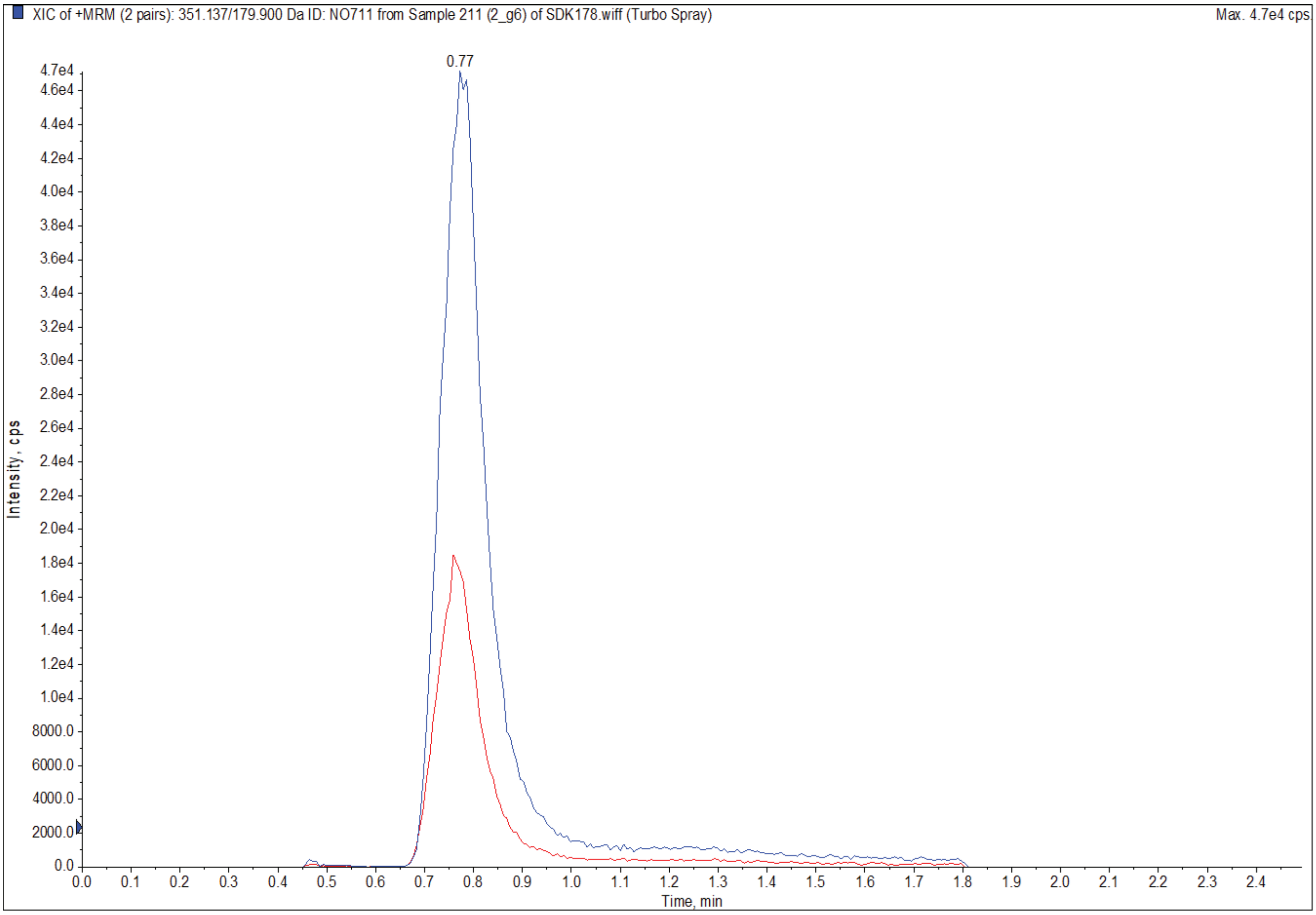

Fig S6o rGAT1$_{EM}$ total binding at 1280 nM NO711

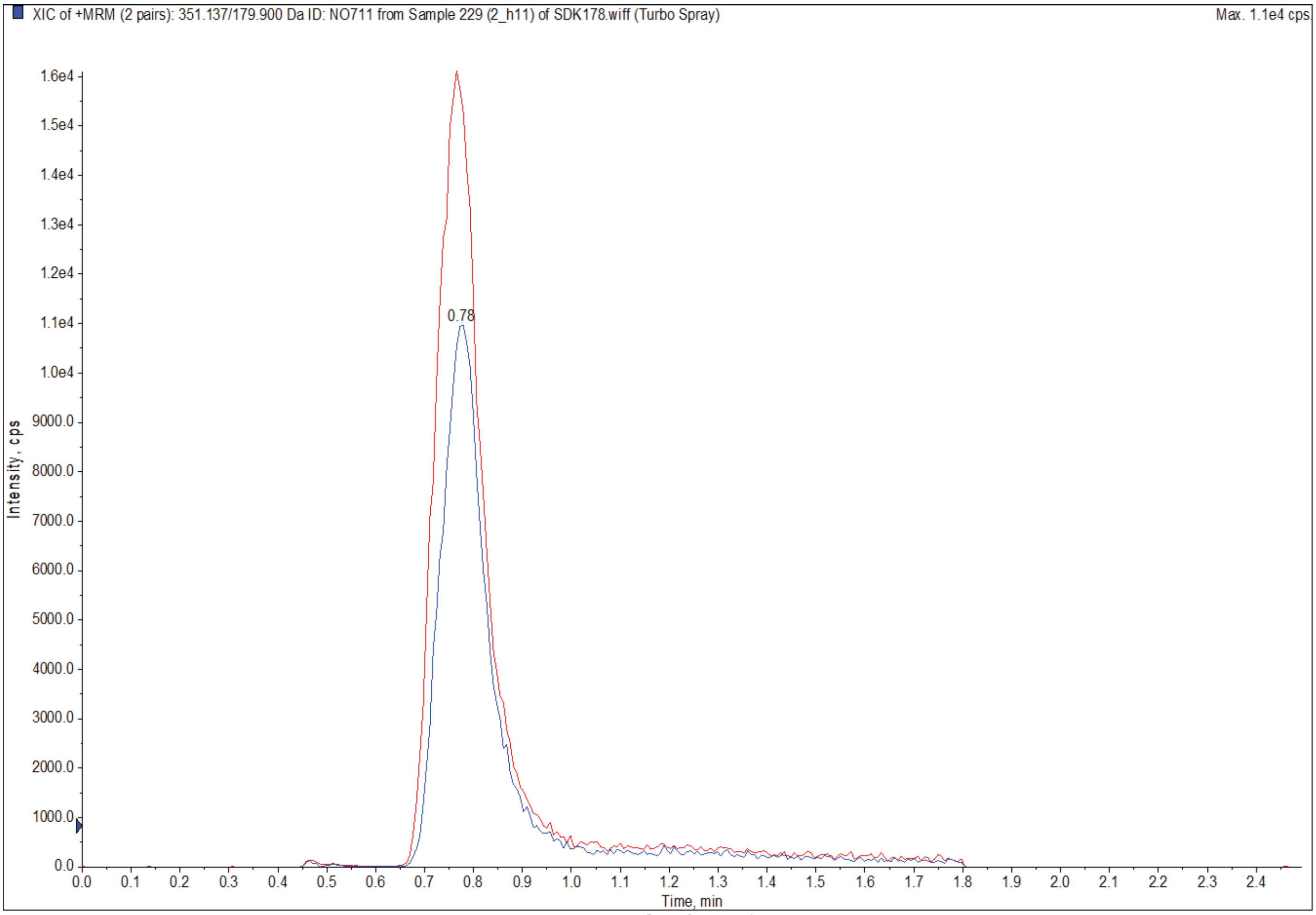

Fig S6p rGAT1$_{EM}$ non-specific binding at 1280 nM NO711

Fig. S7 - Raw chromatograms of MS based binding assays for rGAT1$_{WT}$ construct.

The mass transitions m/z 351/180 (blue) and 361/190 (red), respectively for NO711 and D10-NO711 were recorded. Chromatograms for the following samples (i.e. concentration levels) are depicted: total binding **a**, 2.5 nM, **b**, 5 nM, **c**, 10 nM, **d**, 20 nM, **e**, 40 nM, **g**, 80 nM, **i**, 160 nM, **k**, 320 nM, **m,** 640 nM, **o**,1280 nM, non-specific binding **f**, 40 nM, **h**, 80 nM, **j**, 160 nM, **l**, 320 nM, **n**, 640 nM, **p**, 1280 nM.

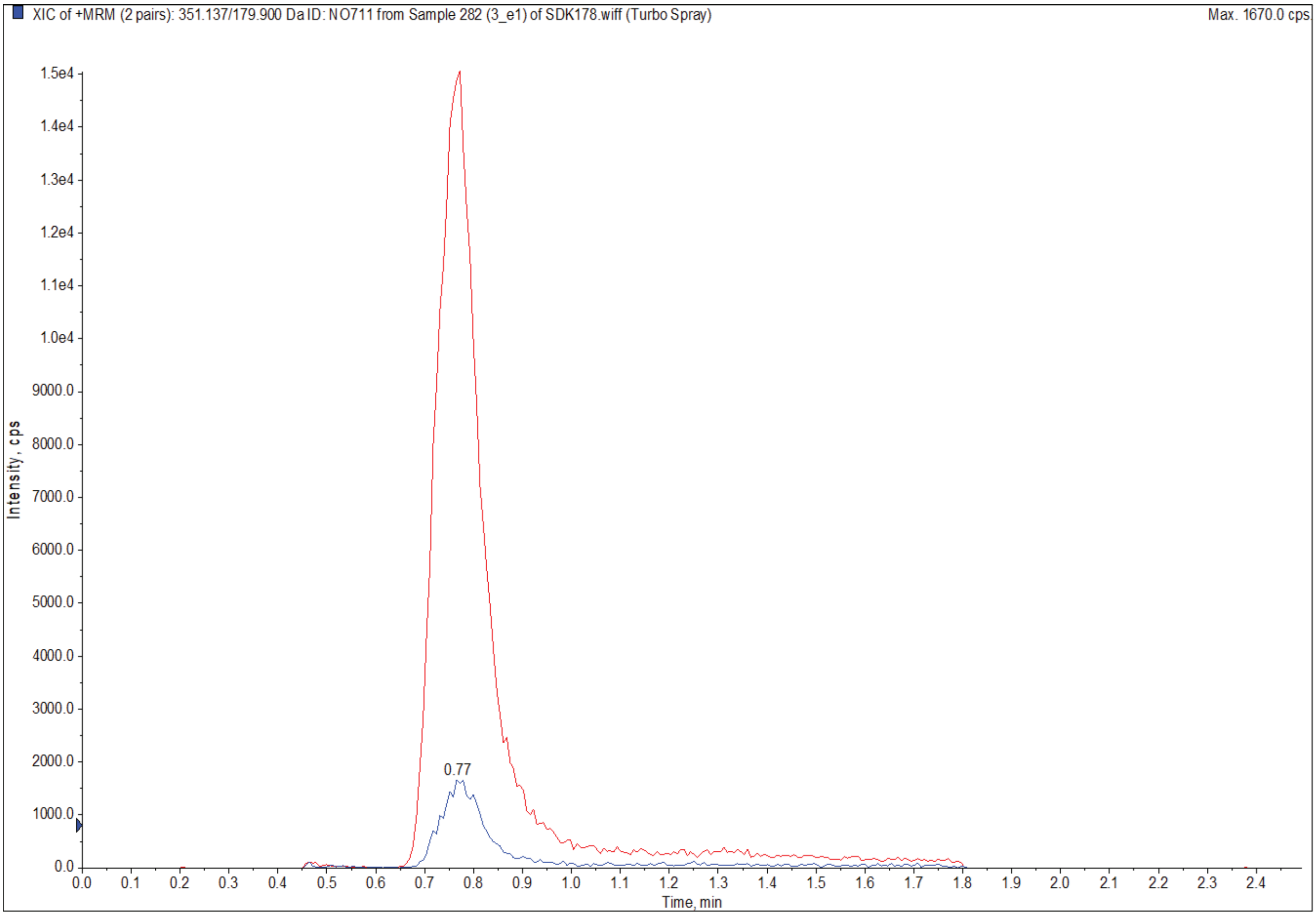

Fig S7a rGAT1$_{WT}$ total binding at 2.5 nM NO711

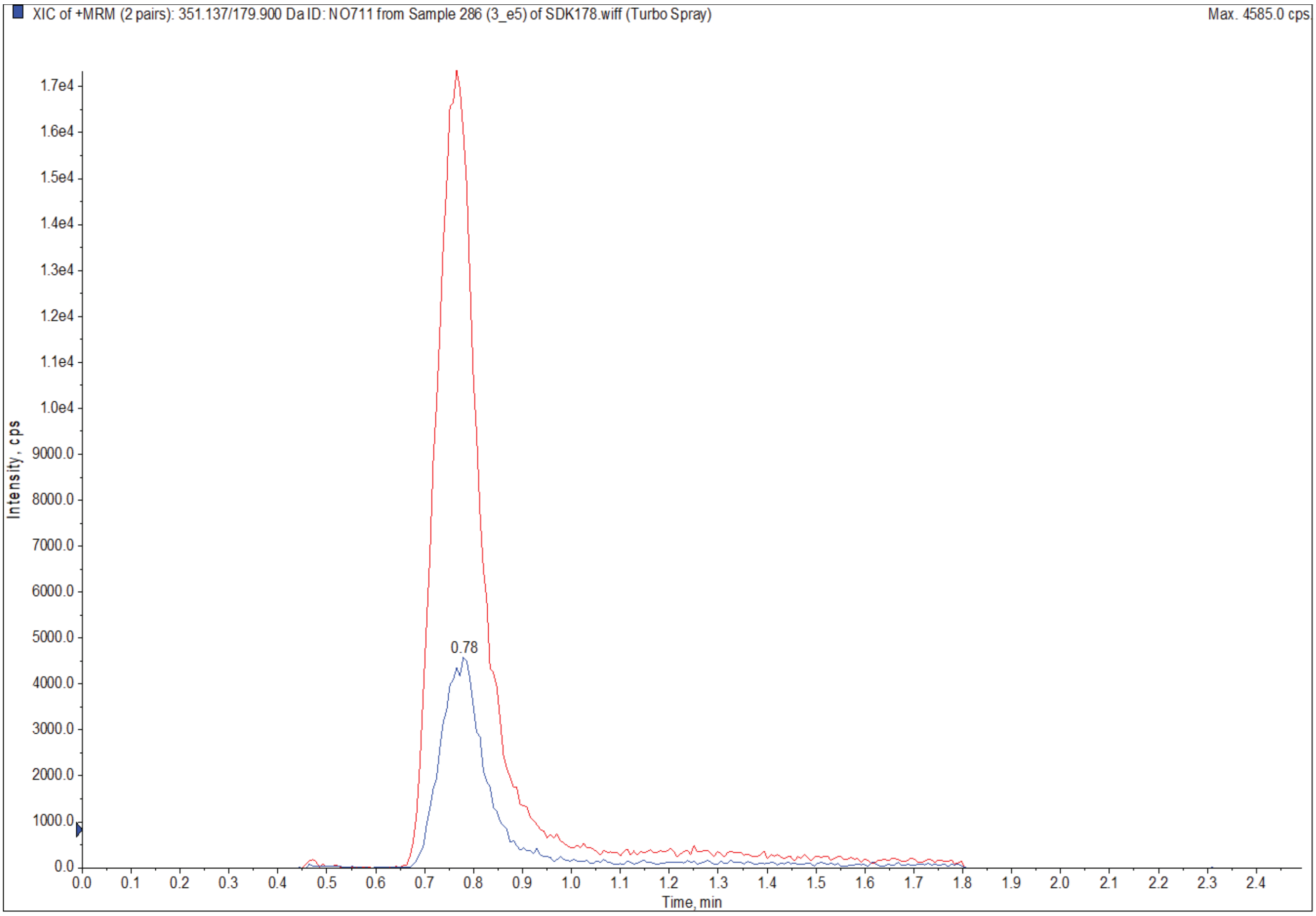

Fig S7b rGAT1$_{WT}$ total binding at 5 nM NO711

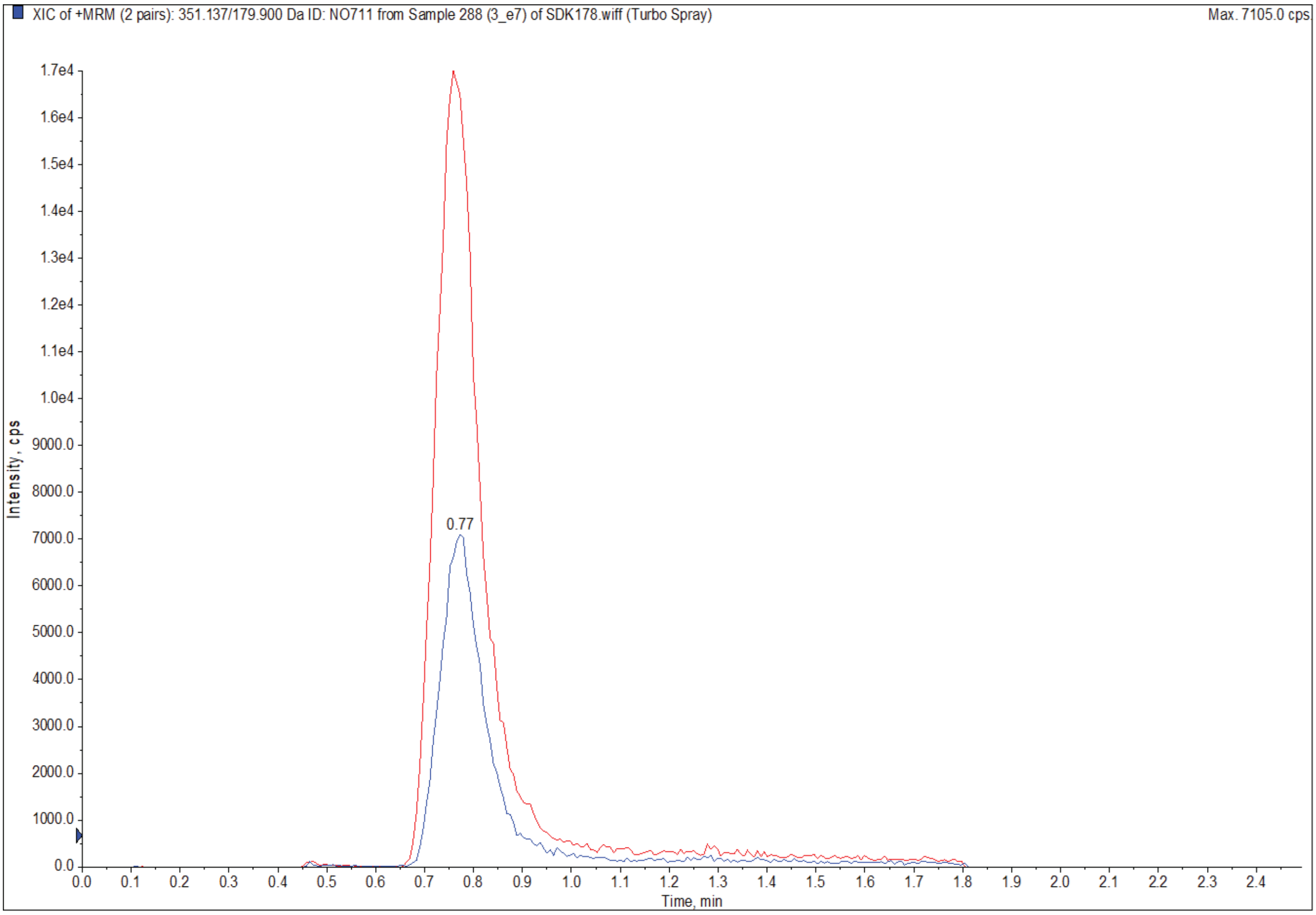

Fig S7c rGAT1$_{WT}$ total binding at 10 nM NO711

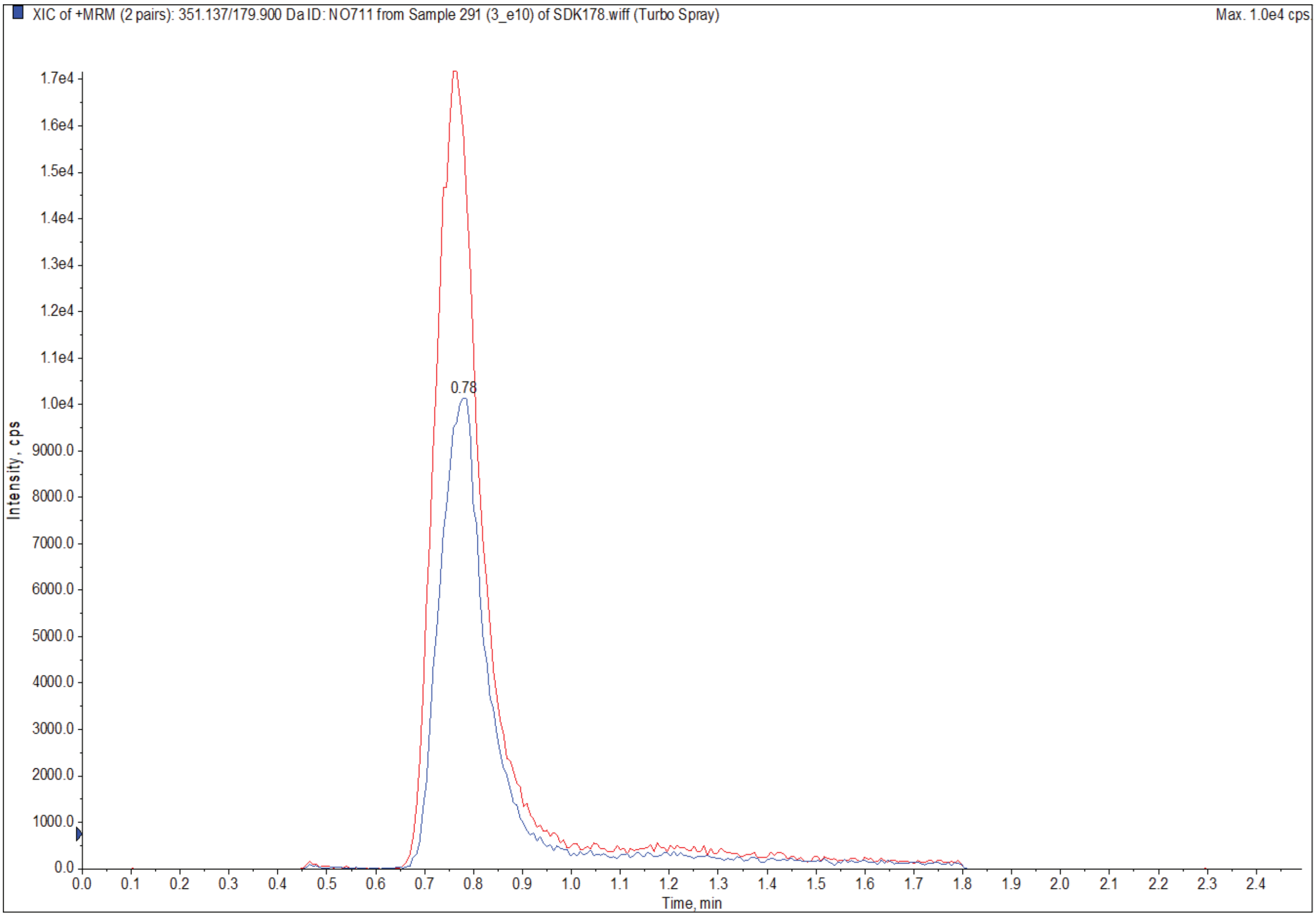

Fig S7d rGAT1$_{WT}$ total binding at 20 nM NO711

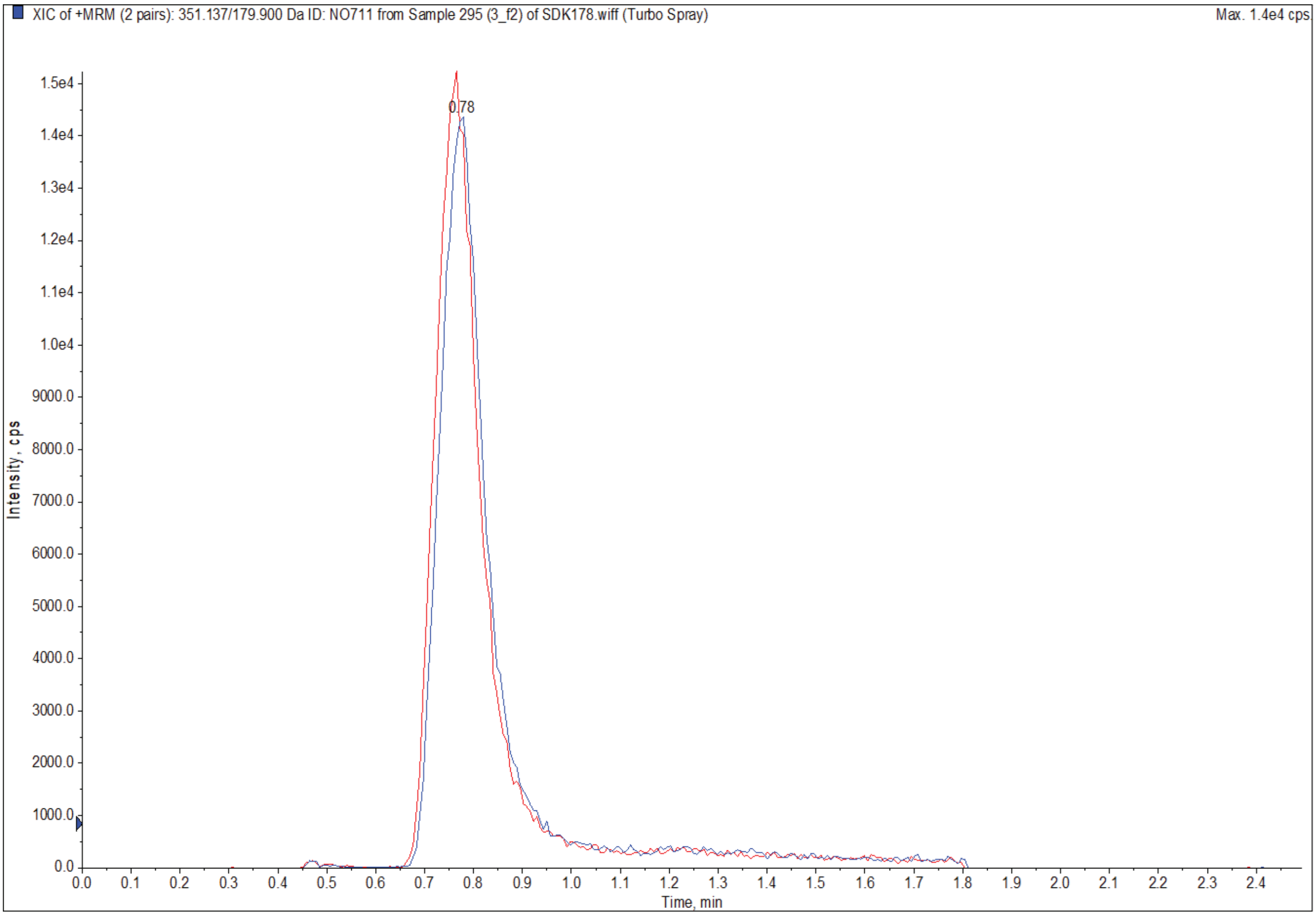

Fig S7e rGAT1$_{WT}$ total binding at 40 nM NO711

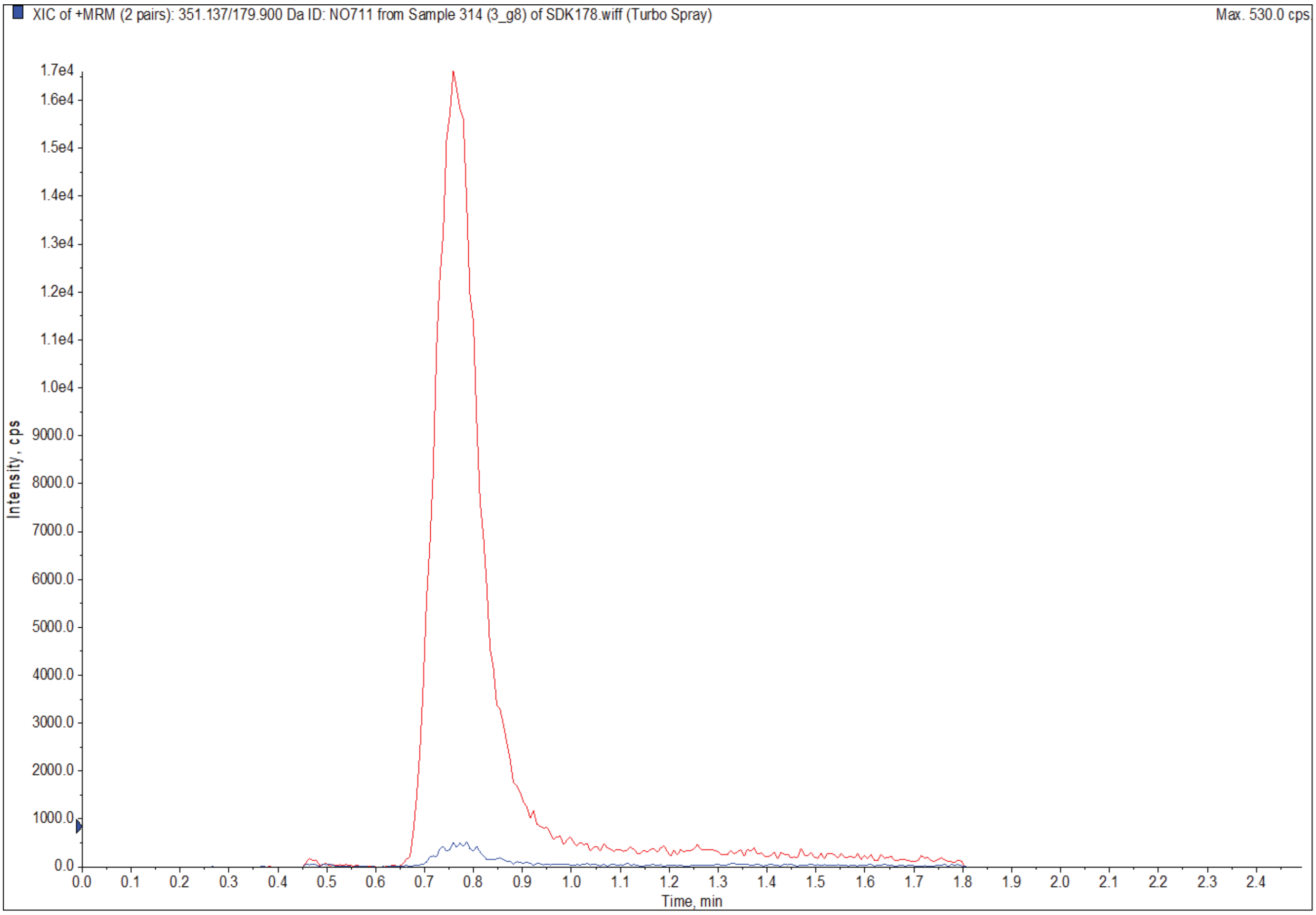

Fig S7f rGAT1$_{WT}$ non-specific binding at 40 nM NO711

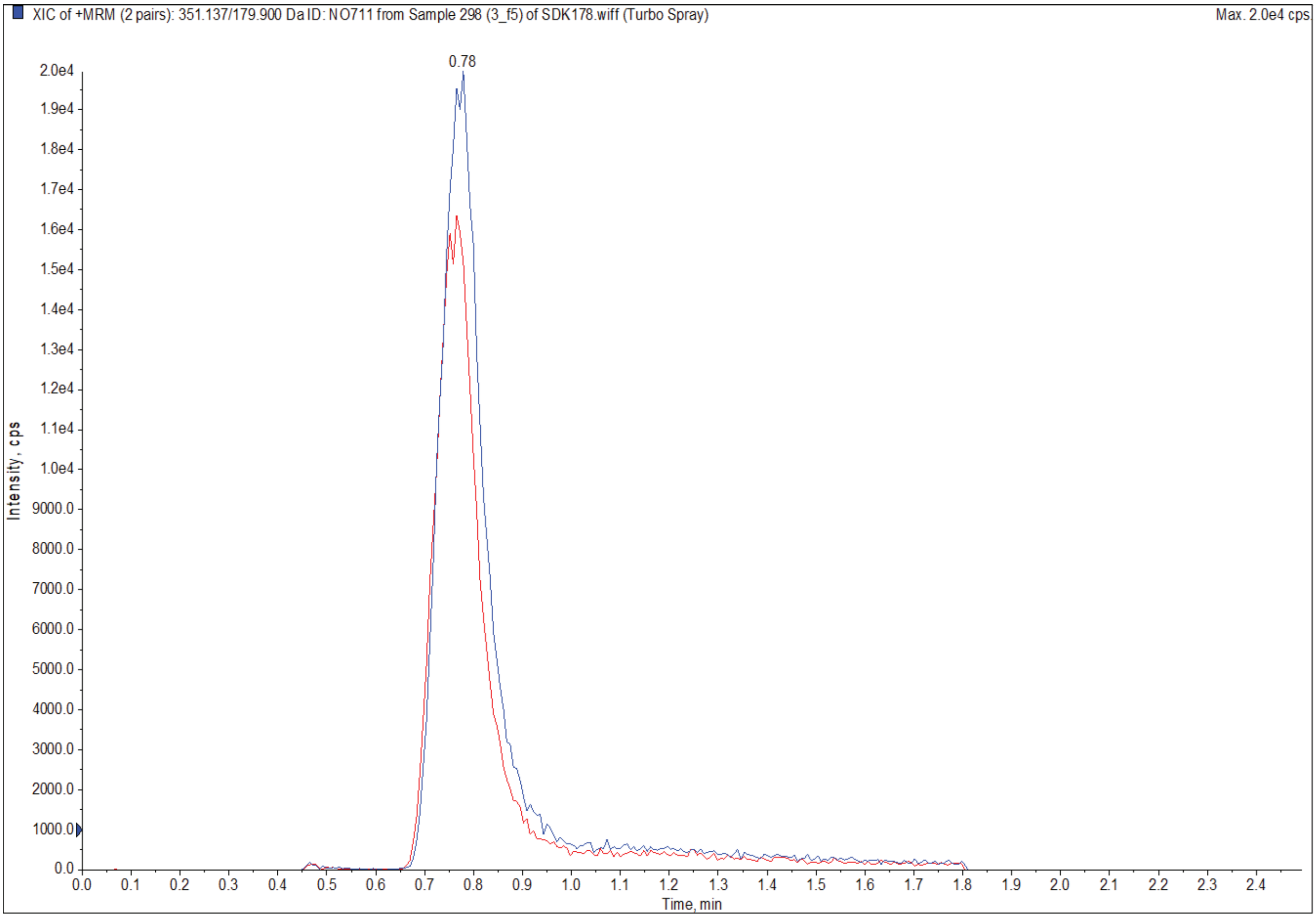

Fig S7g rGAT1$_{WT}$ total binding at 80 nM NO711

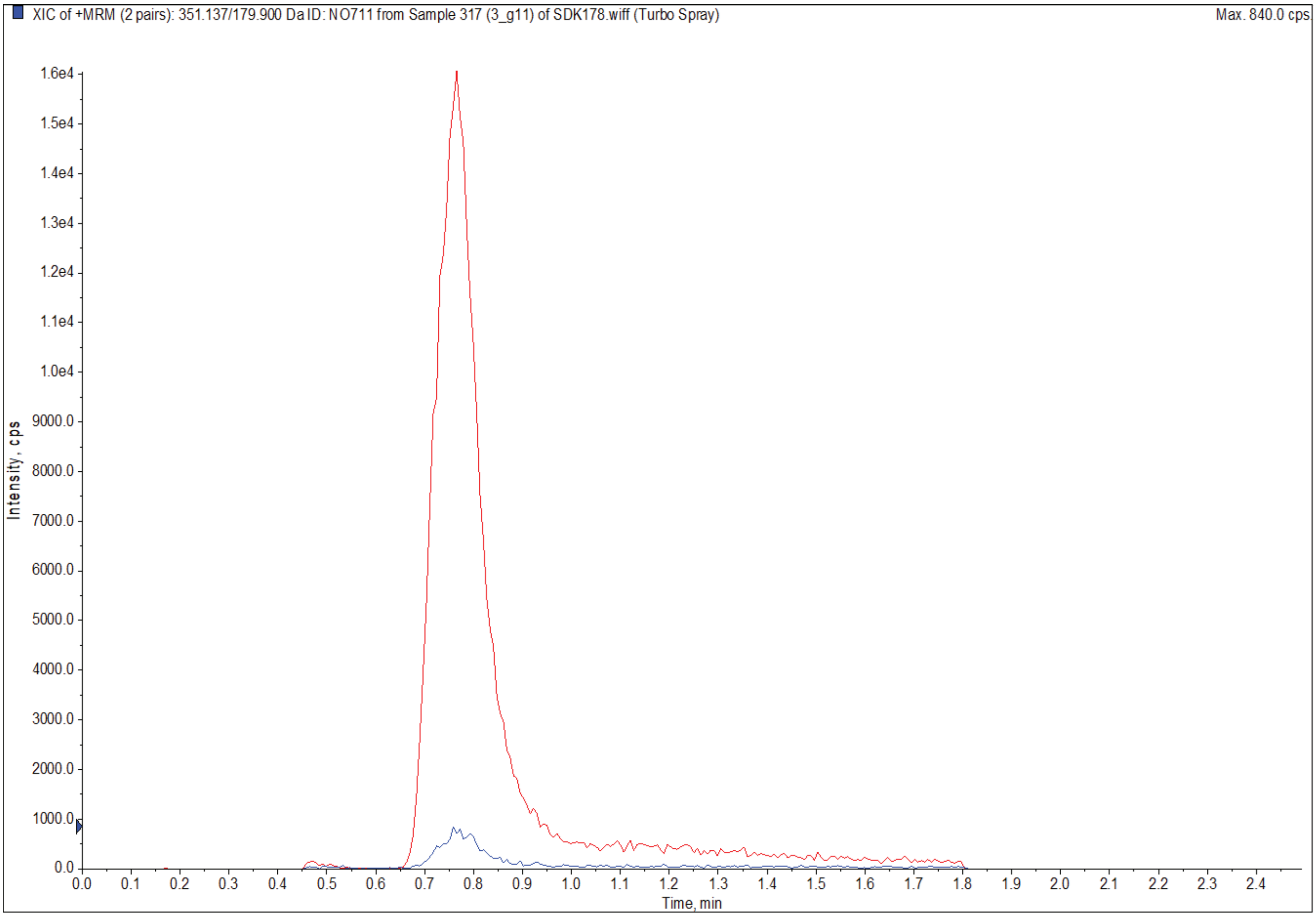

Fig S7h rGAT1$_{WT}$ non-specific binding at 80 nM NO711

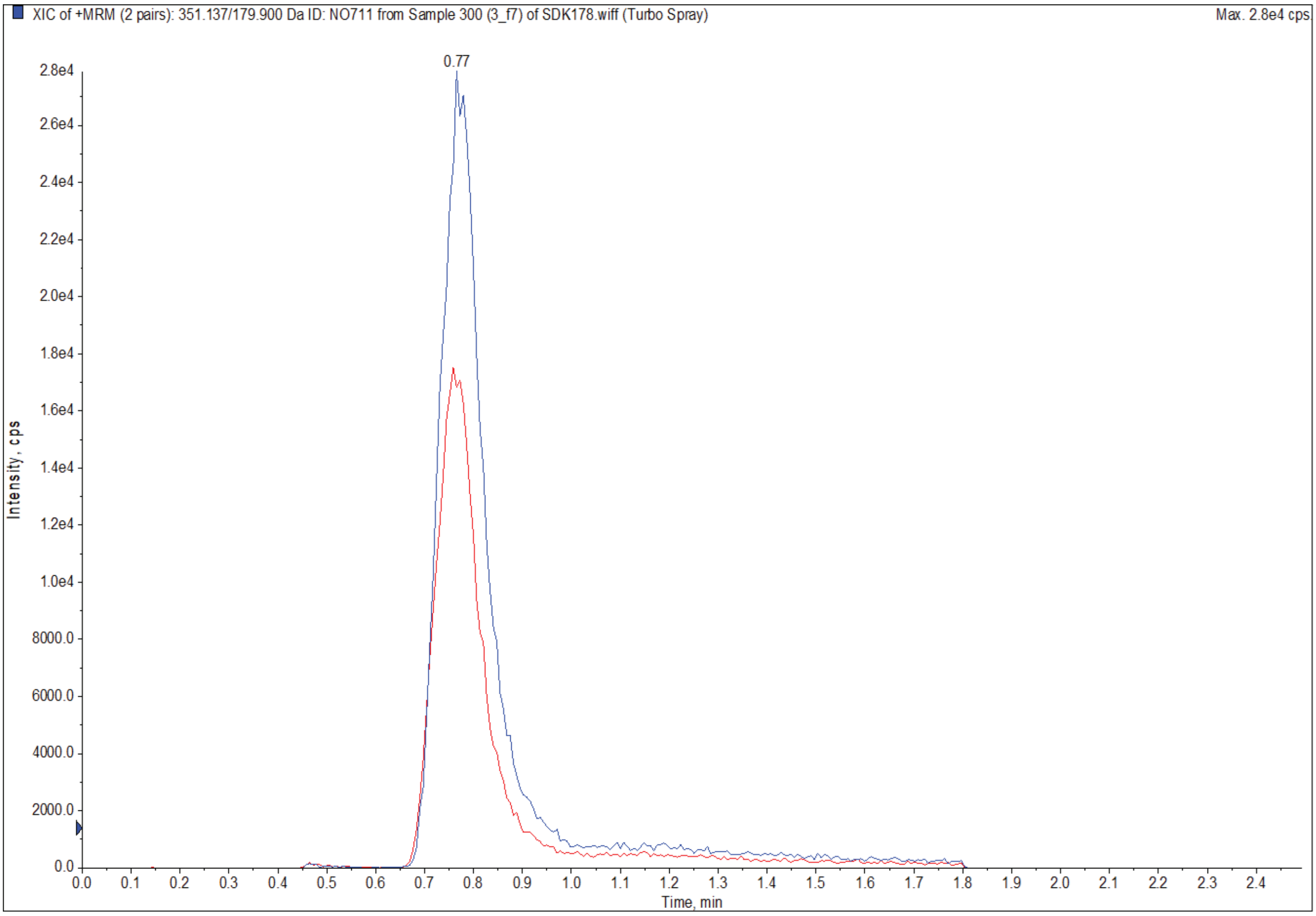

Fig S7i rGAT1$_{WT}$ total binding at 160 nM NO711

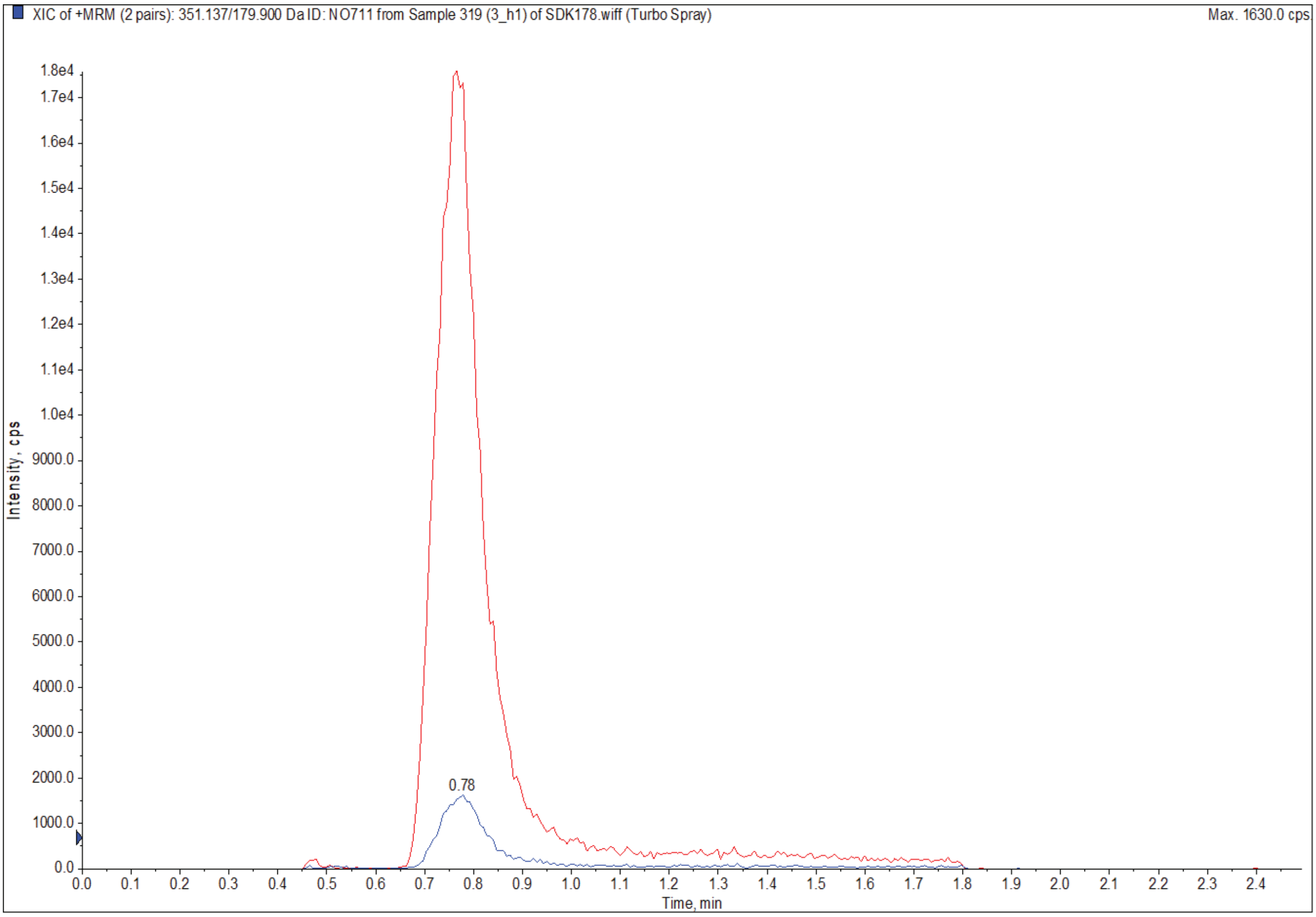

Fig S7j rGAT1$_{WT}$ non-specific binding at 160 nM NO711

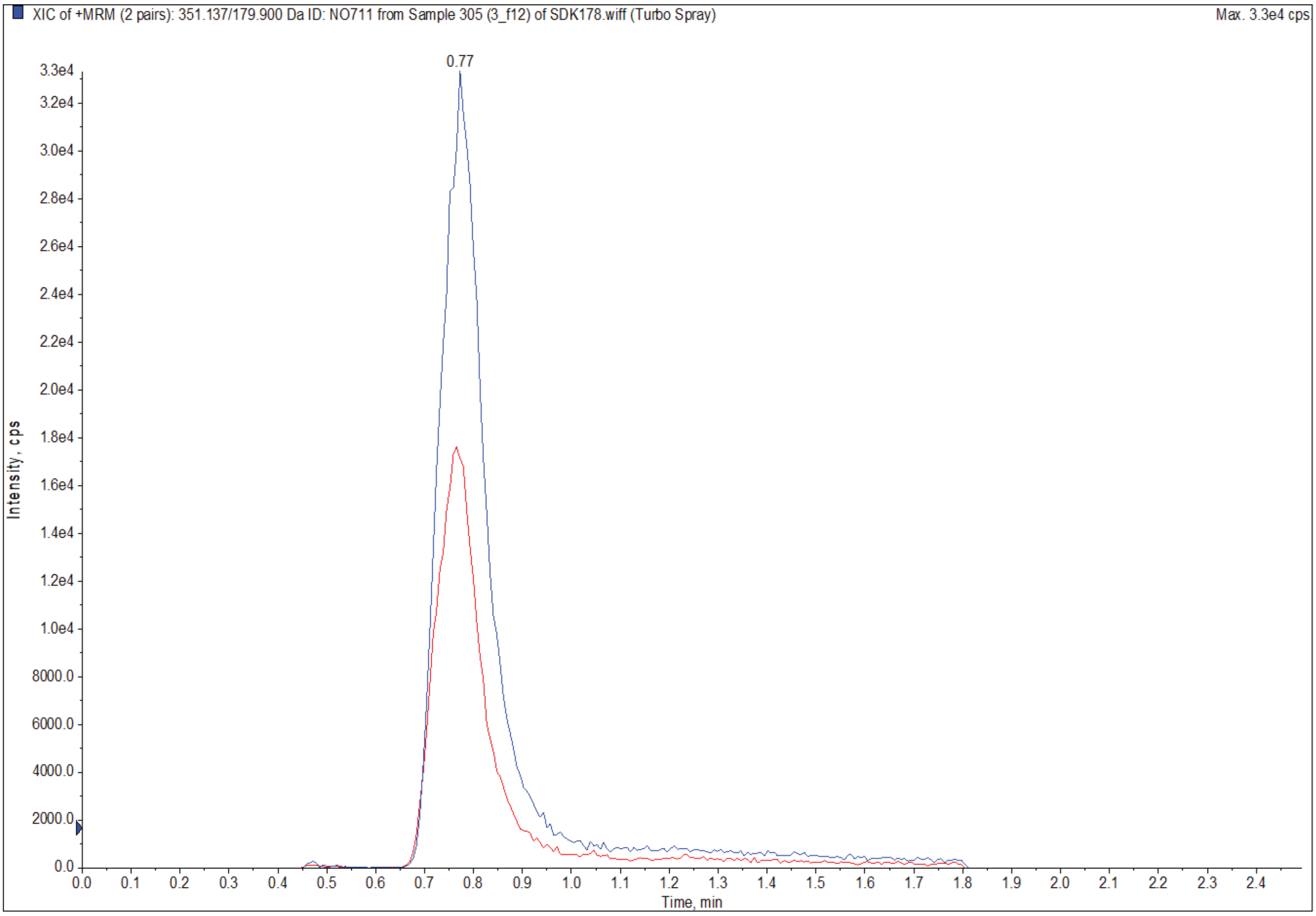

Fig S7k rGAT1$_{WT}$ total binding at 320 nM NO711

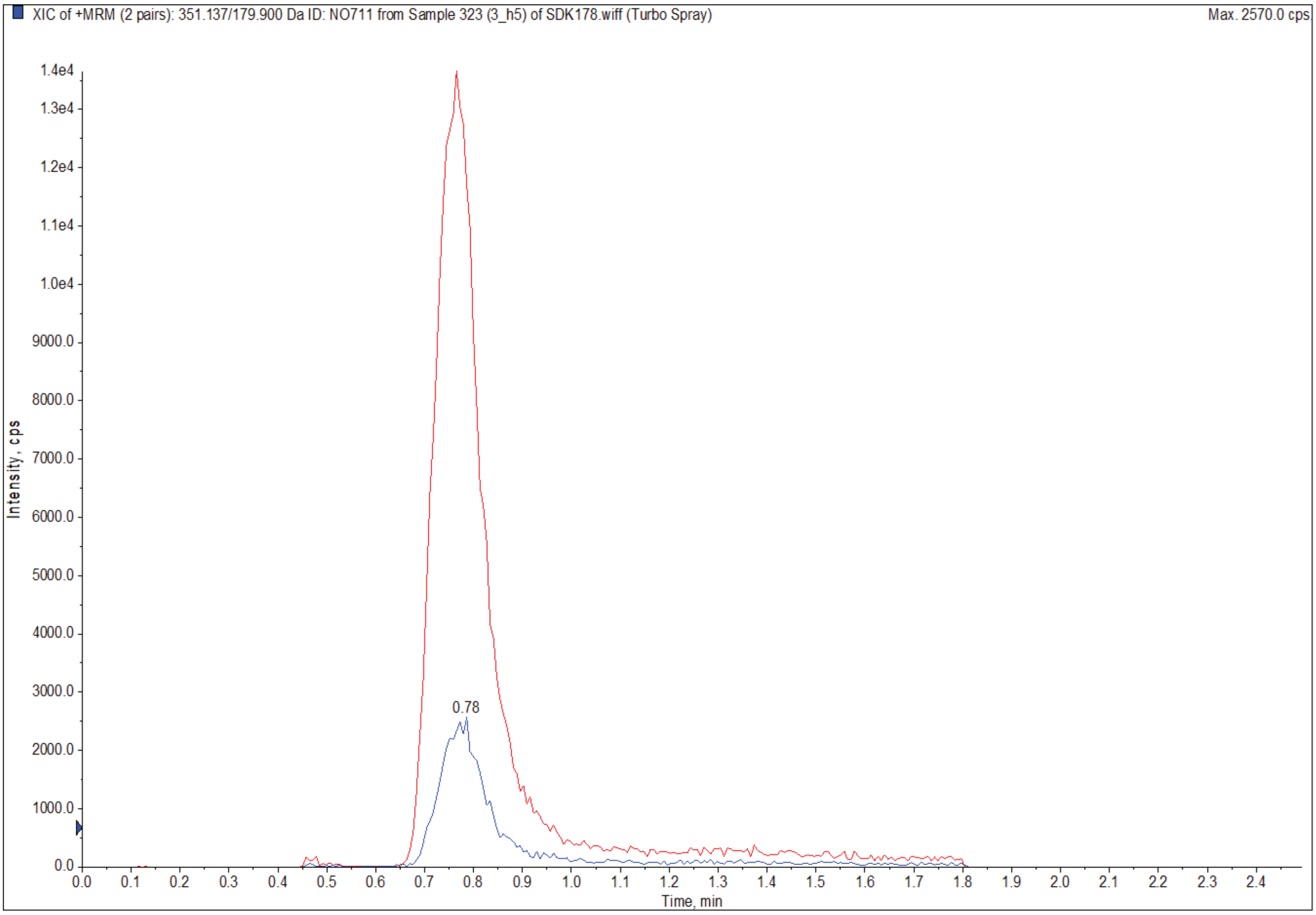

Fig S7l rGAT1$_{WT}$ non-specific binding at 320 nM NO711

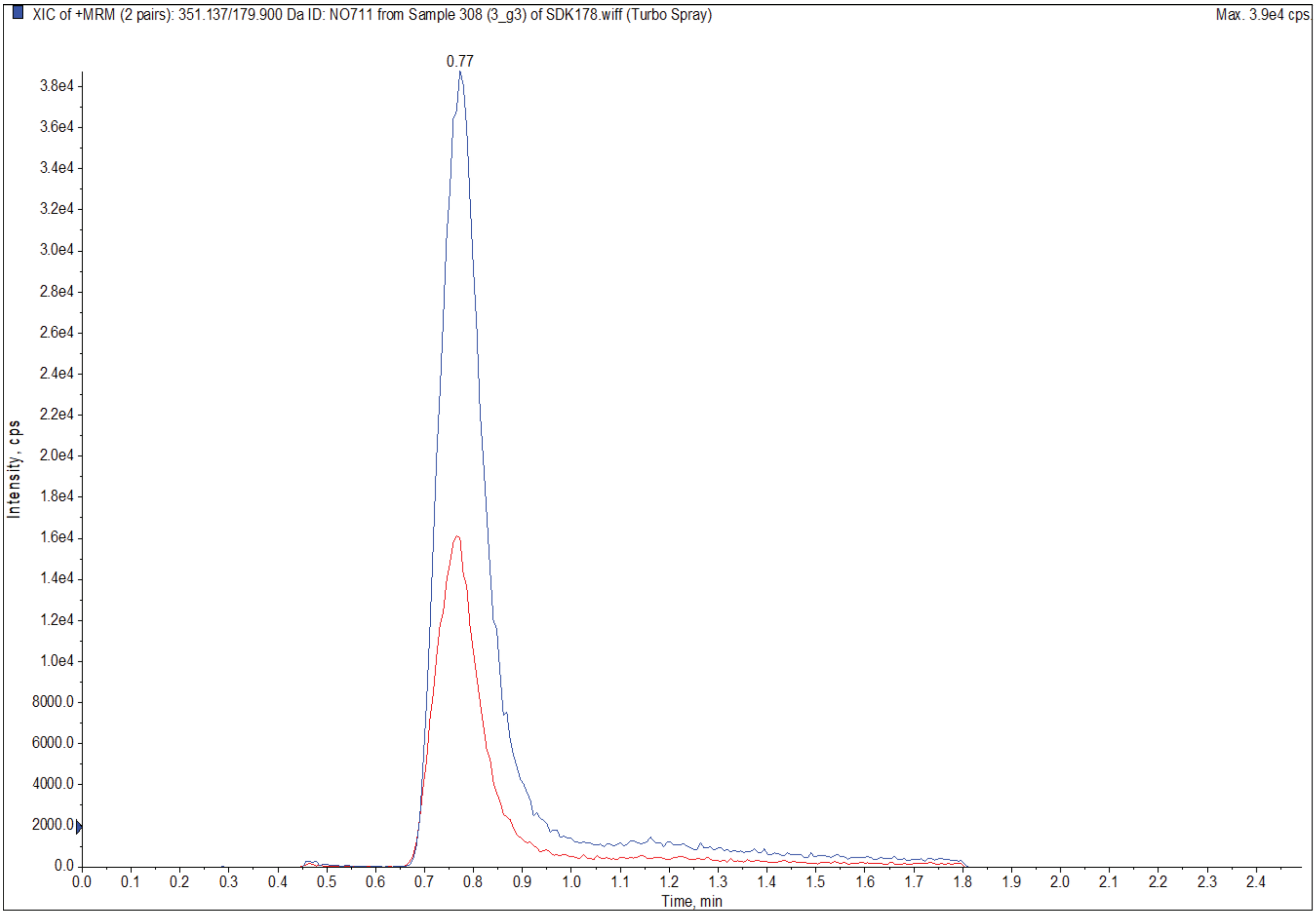

Fig S7m rGAT1$_{WT}$ total binding at 640 nM NO711

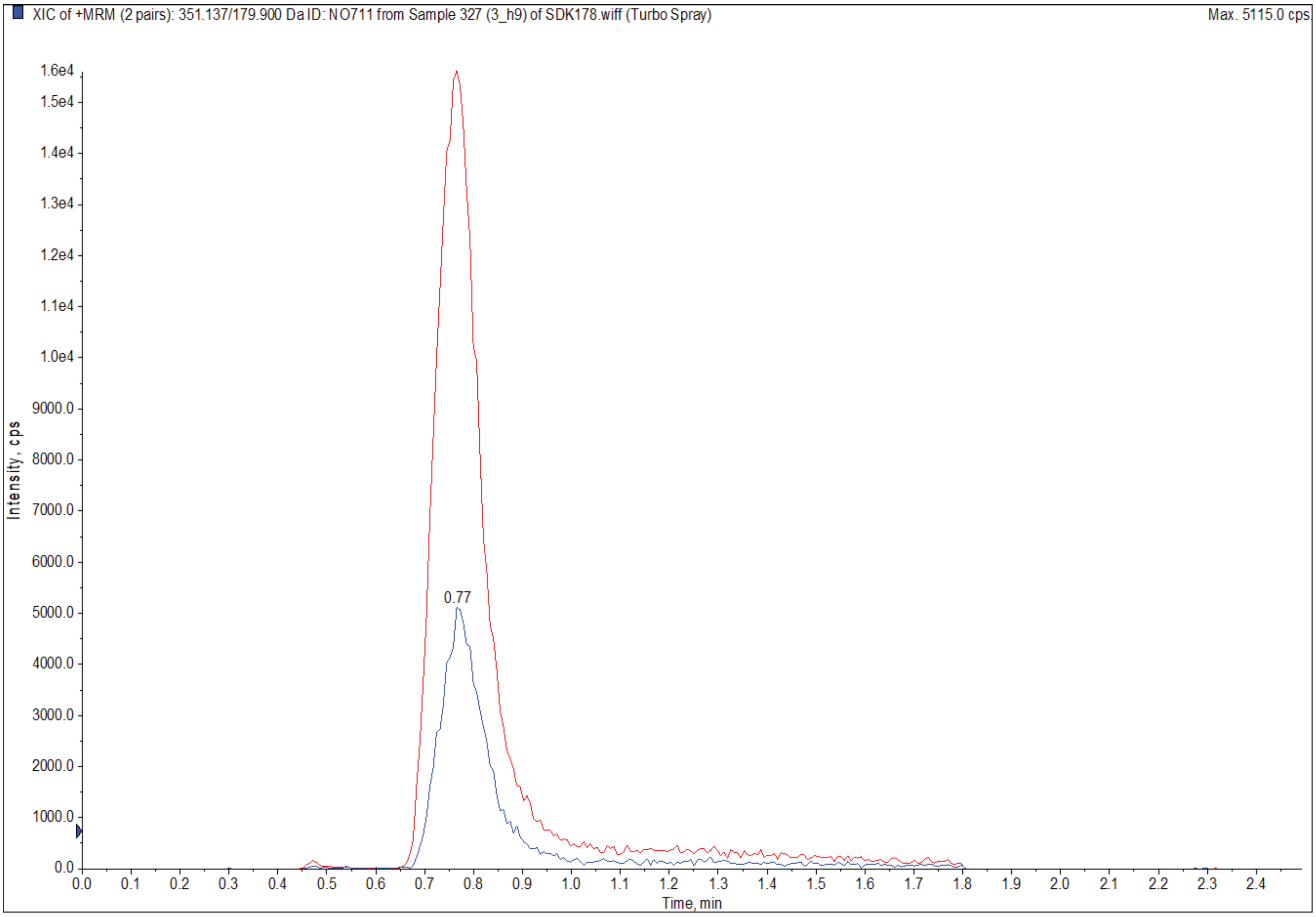

Fig S7n rGAT1$_{WT}$ non-specific binding at 640 nM NO711

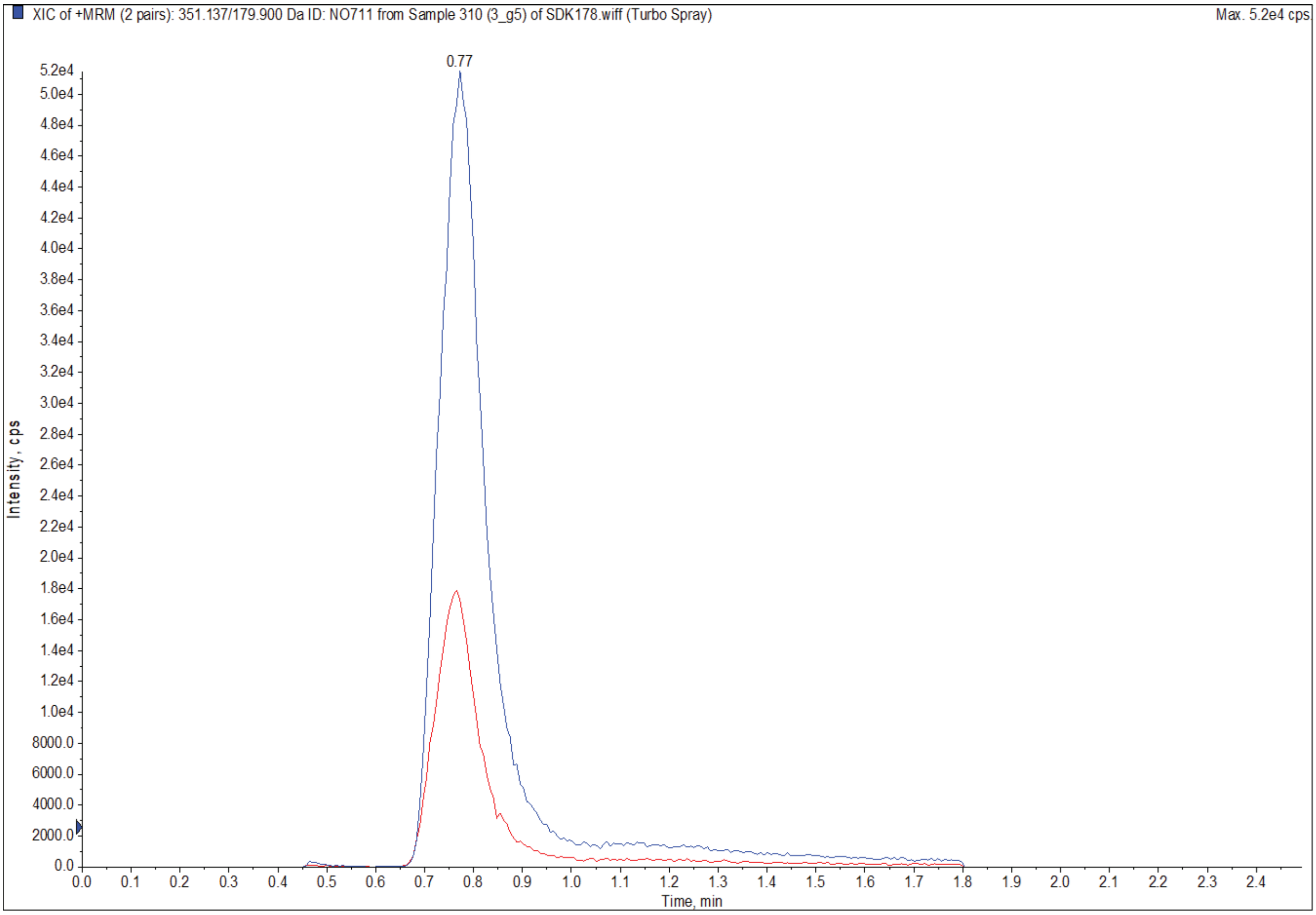

Fig S7o rGAT1$_{WT}$ total binding at 1280 nM NO711

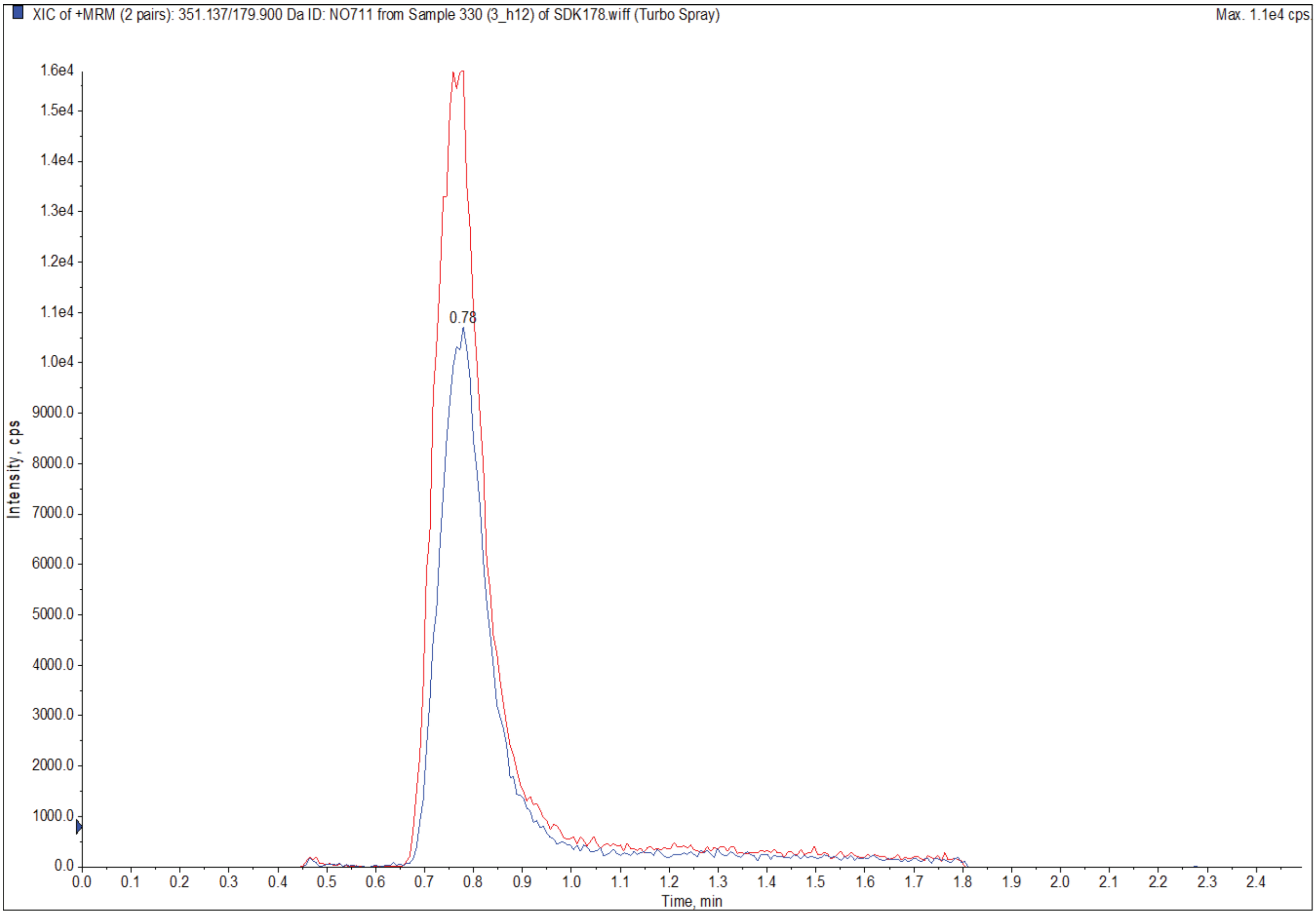

Fig S7p rGAT1$_{WT}$ non-specific binding at 1280 nM NO711

Fig. S8 - Raw chromatograms of GABA detection in HEK293 cells.

The mass transitions m/z 104.0/87.0 (blue), 104.0/69.0 (red), 110.0/93.0 (green) and 110.0/73.0 (grey), respectively for GABA and D6-GABA were recorded (note that the colors of the original chromatograms are not the same as shown in ED Fig 7). **a**, Chromatogram of pure HEK293 cell sample. **b**, Chromatogram of cell sample spiked with D6-GABA. **c**, Chromatogram of cell sample spiked with 25 nM GABA and D6-GABA. **d**, Chromatogram of cell sample spiked with 50 nM GABA and D6-GABA. **e**, Chromatogram of blank prepared in the absence of HEK293 cells. **f**, Chromatogram of 20 nM GABA standard prepared in the absence of HEK293 cells. Standard addition curve for GABA calculation (**g**, no GABA, **h**,10 nM GABA, **i**,25 nM GABA, **j**, 50 nM GABA, **k**, 100 nM GABA, **l**, 250 nM GABA).

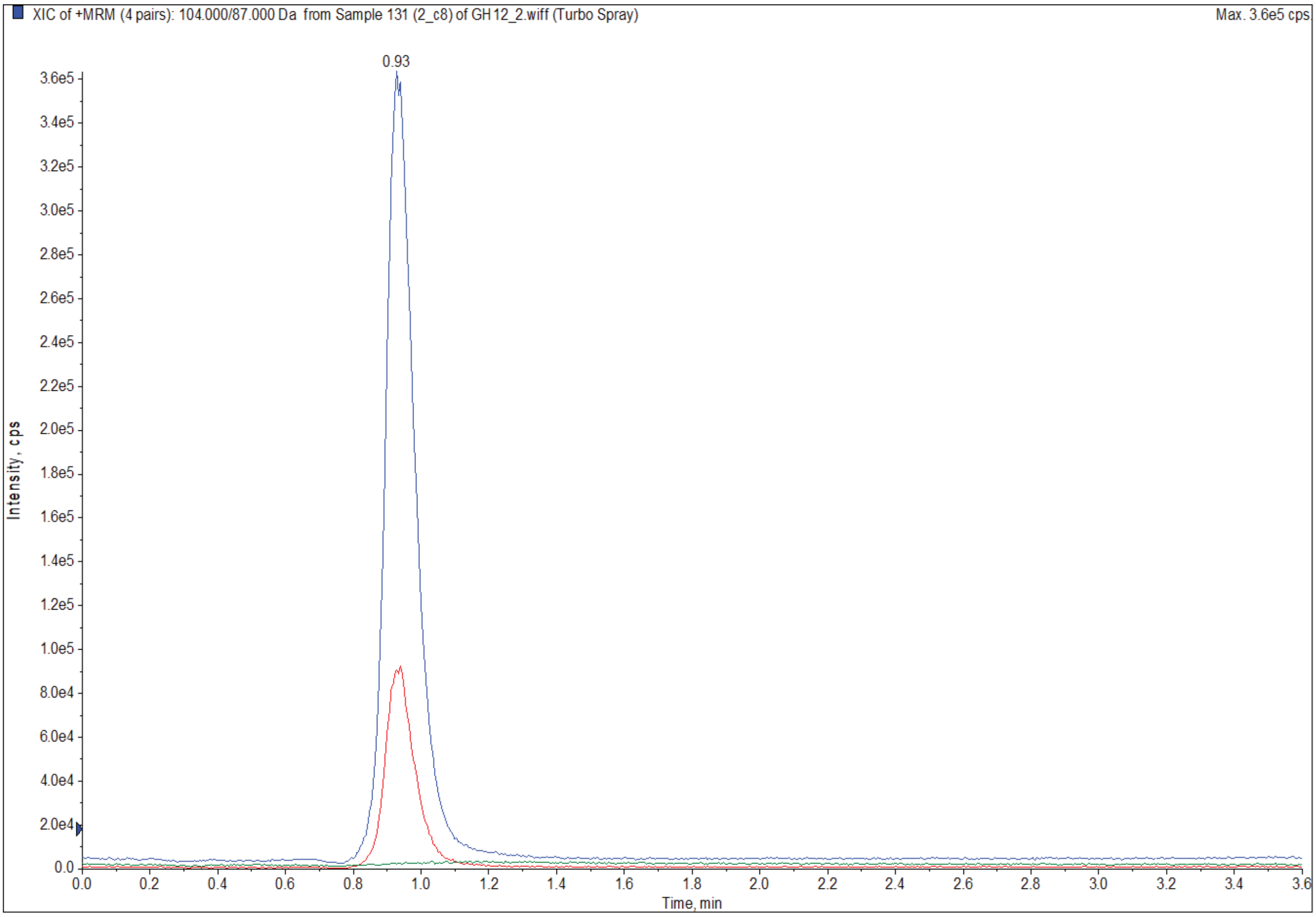

Fig S8a

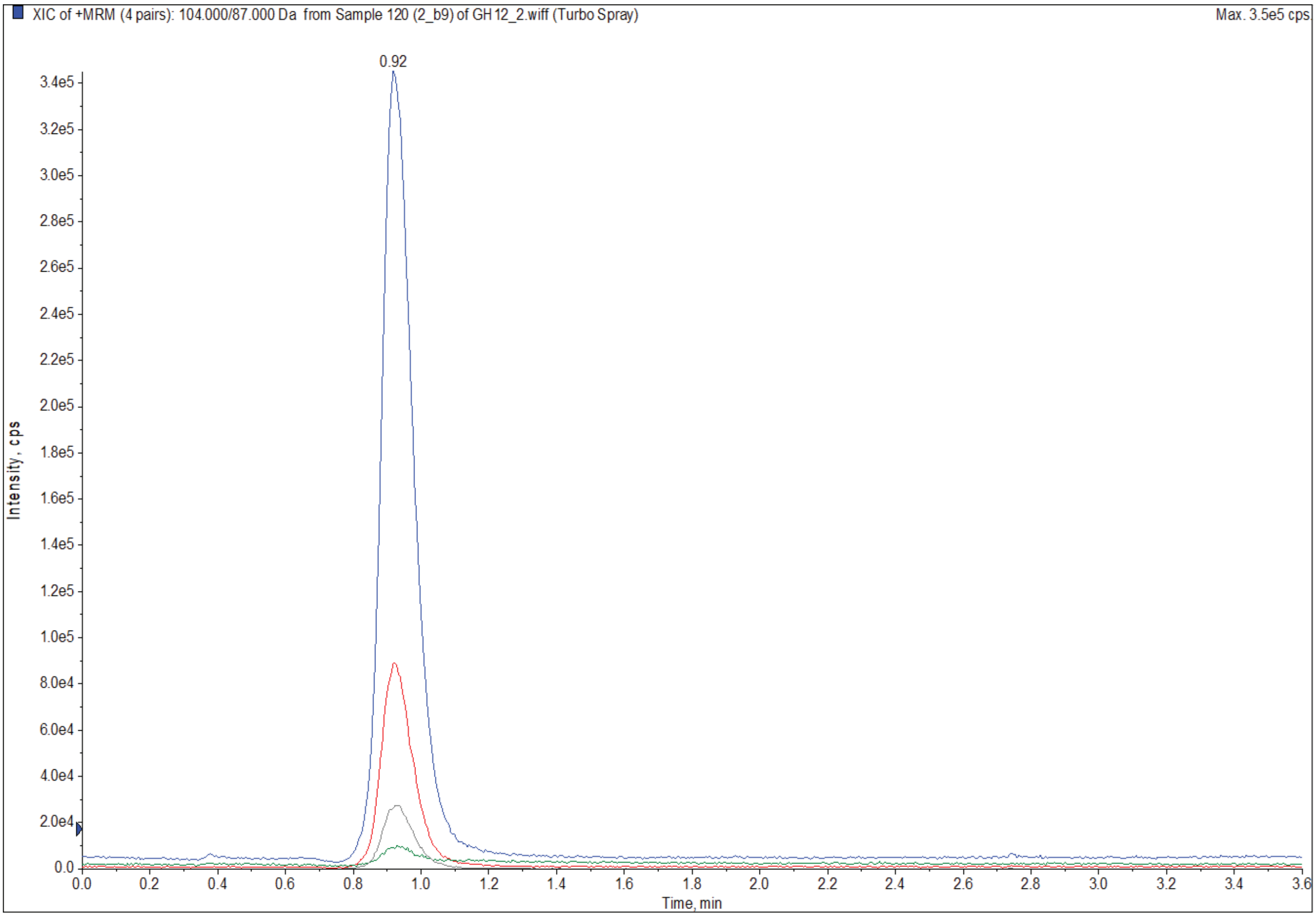

Fig S8b

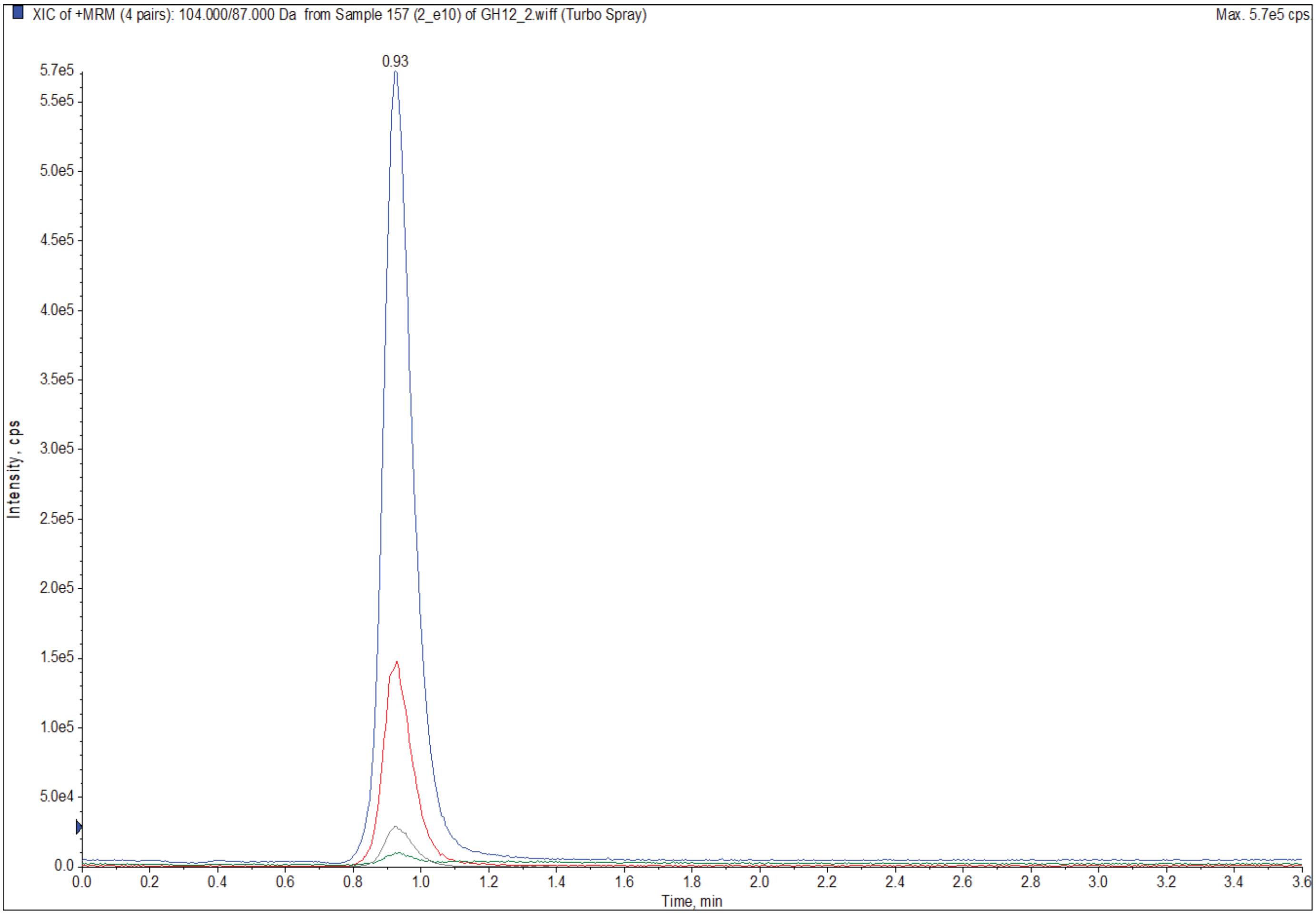

Fig S8c

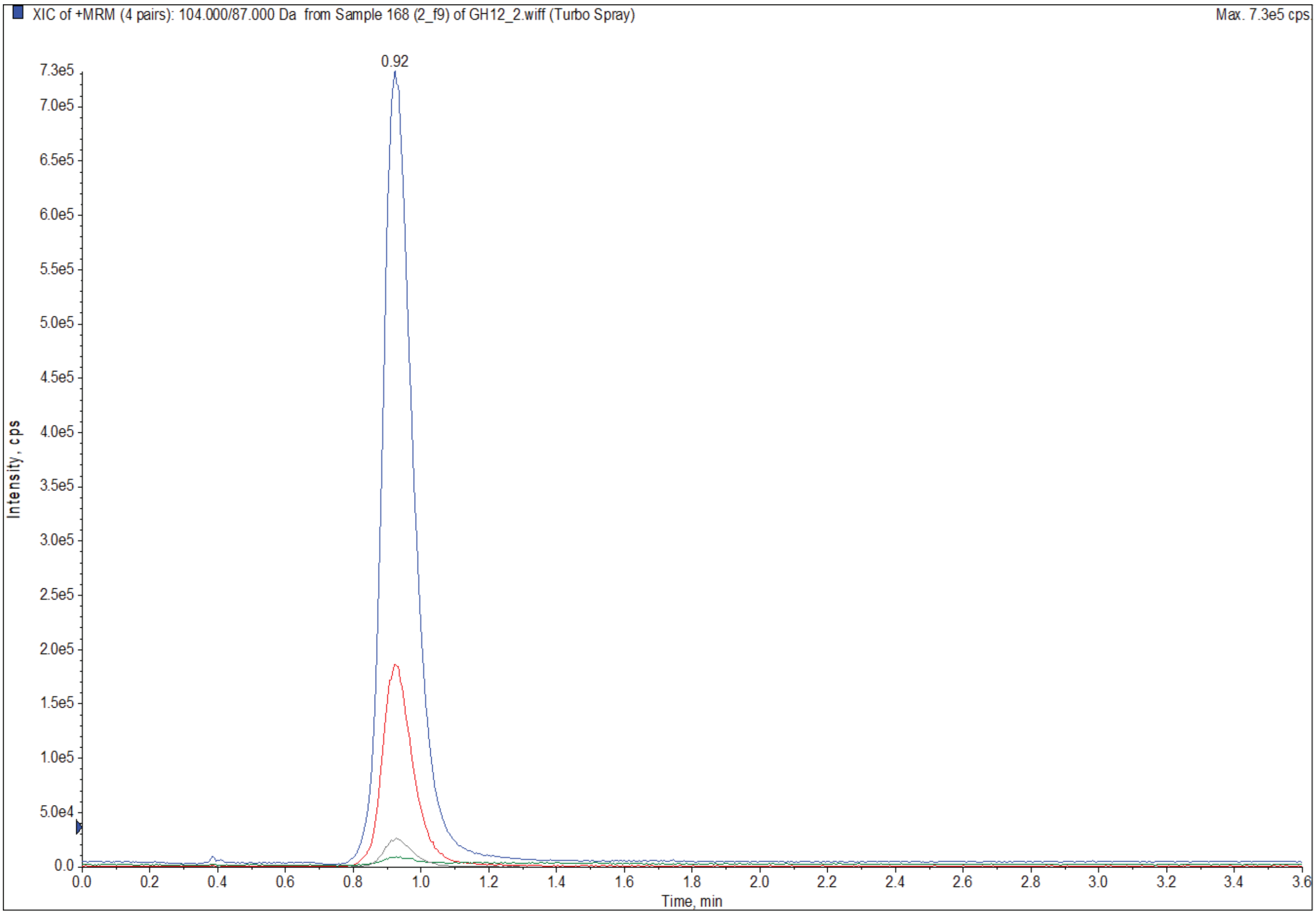

Fig S8d

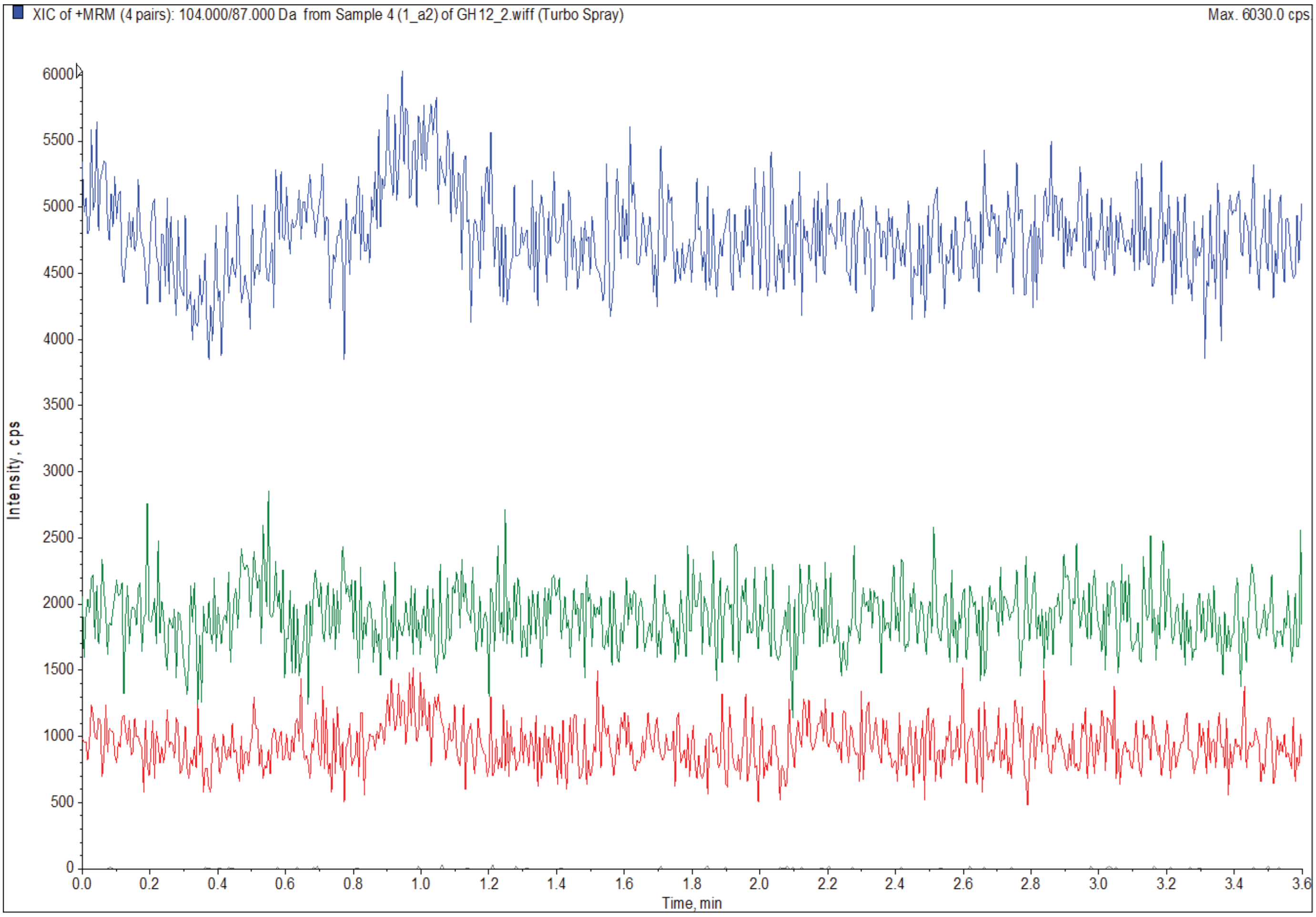

Fig S8e

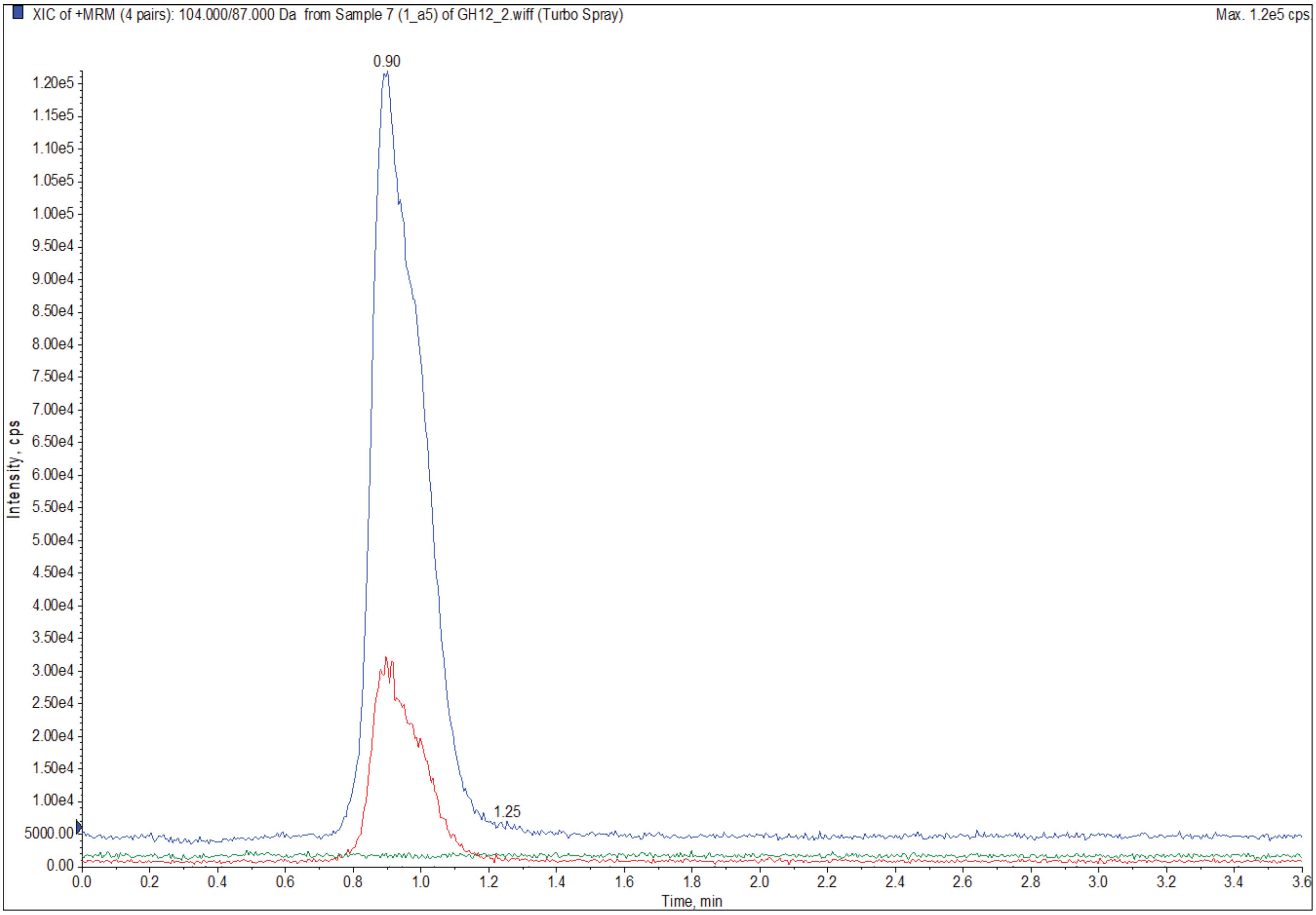

Fig S8f

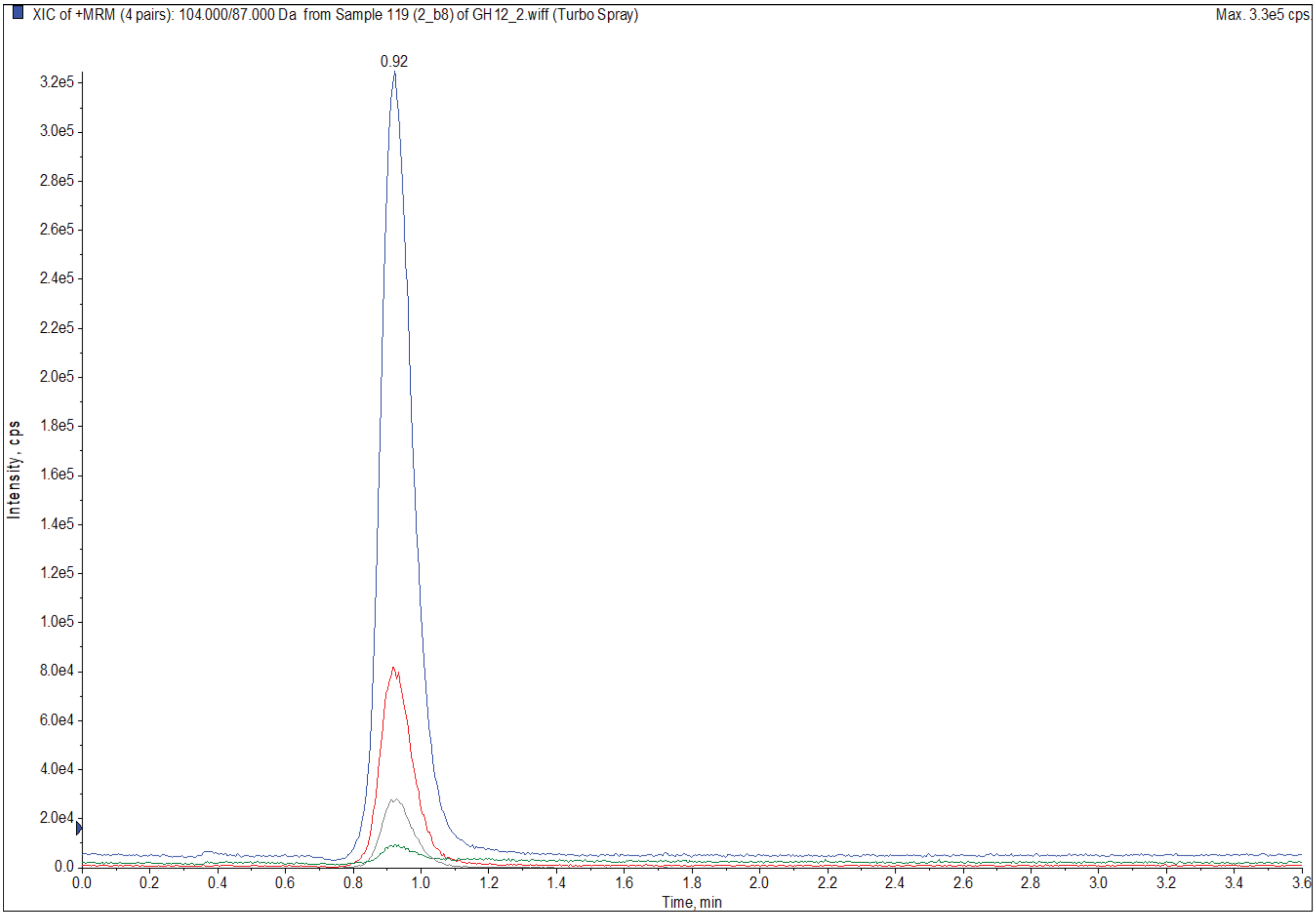

Fig S8g – no GABA

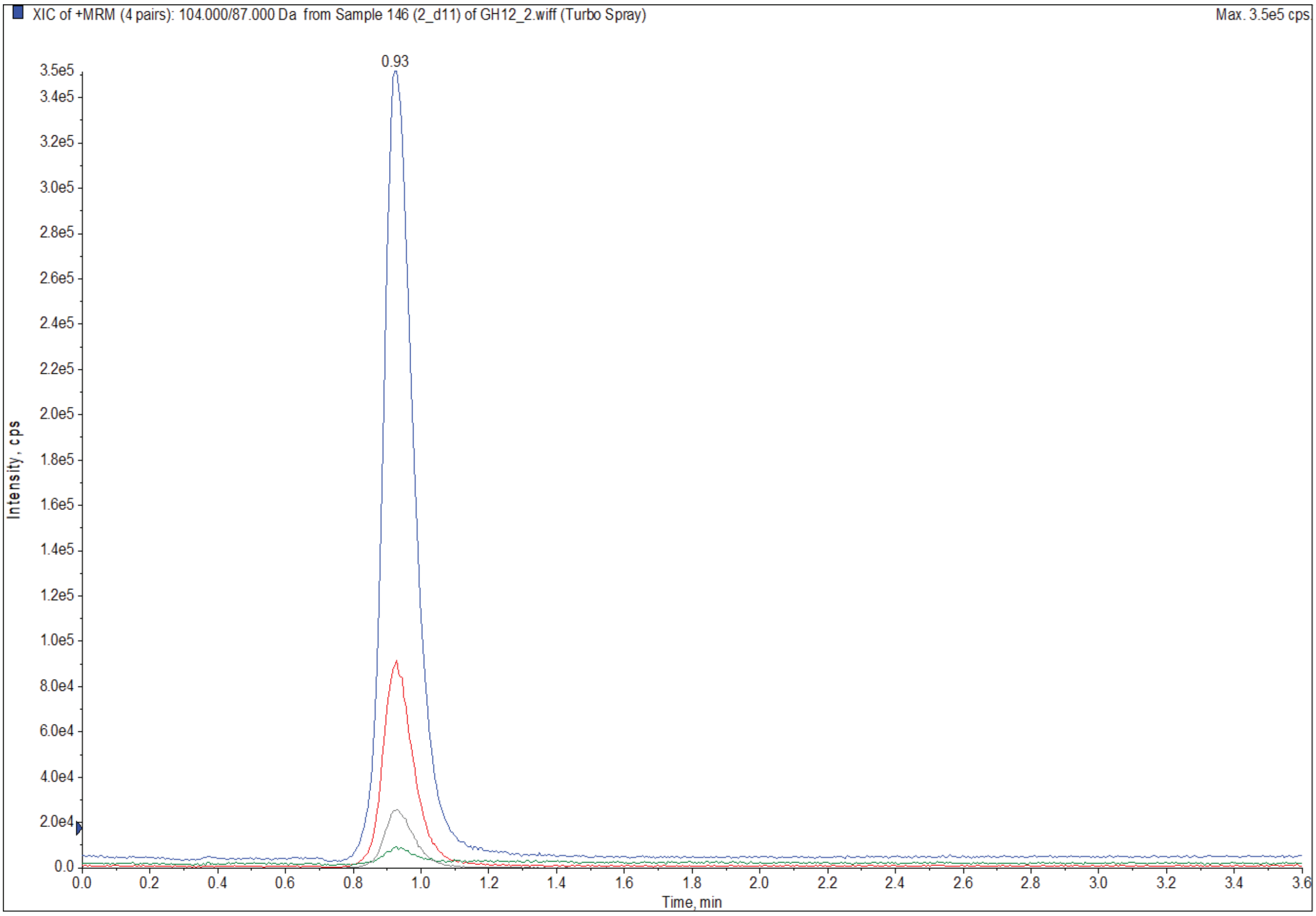

Fig S8h – 10 nM GABA

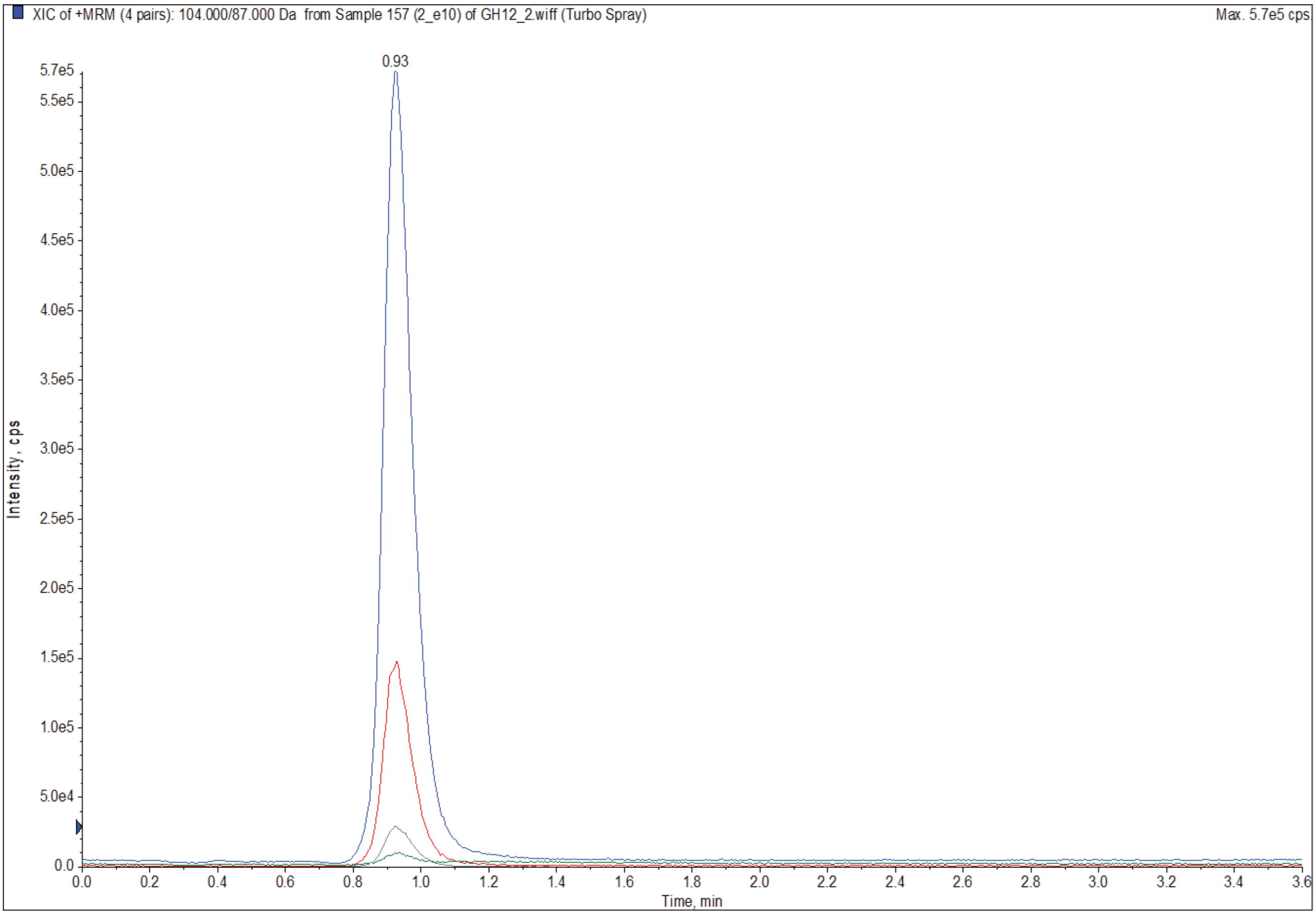

Fig S8i – 25 nM GABA

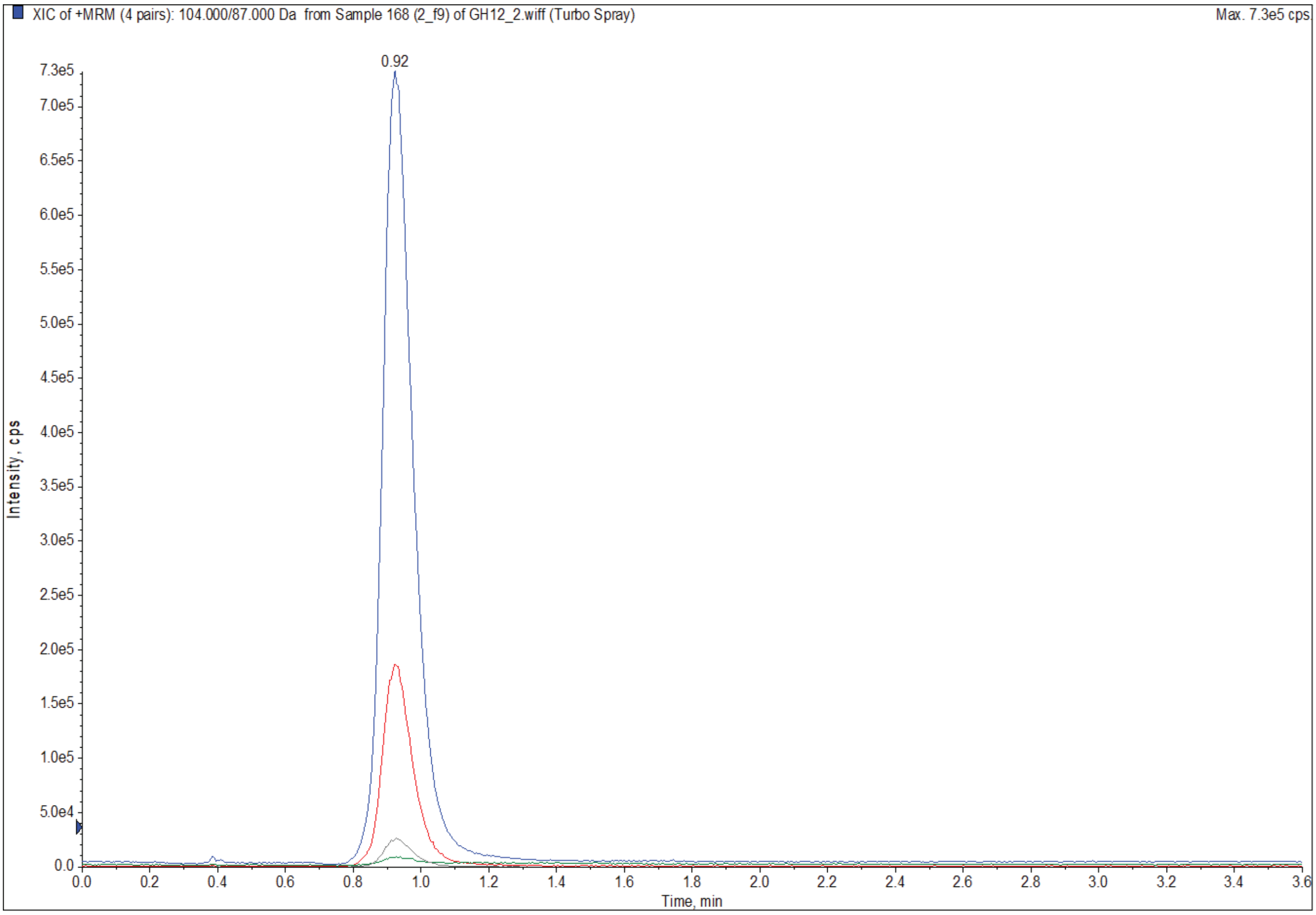

Fig S8j – 50 nM GABA

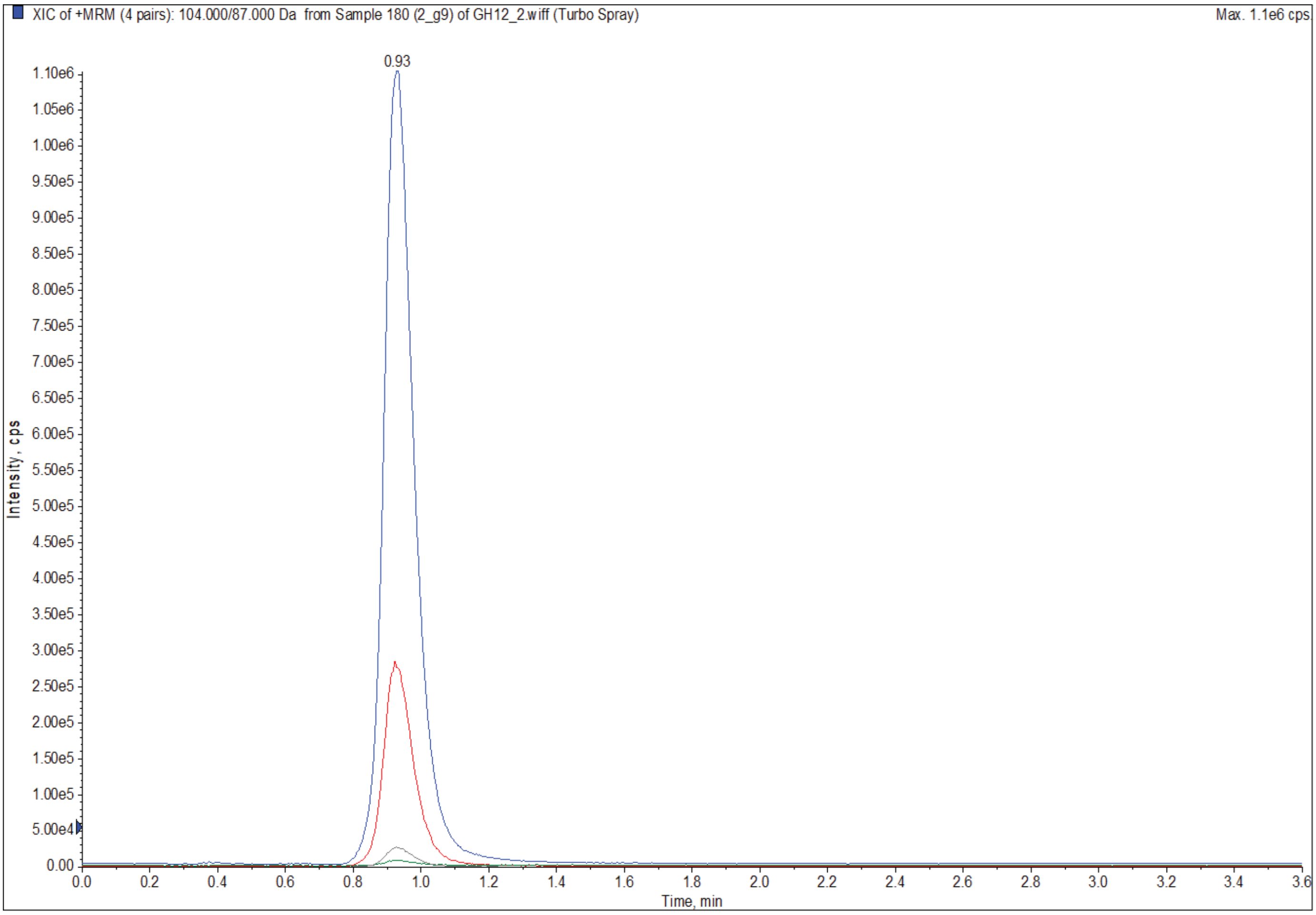

Fig S8k – 100 nM GABA

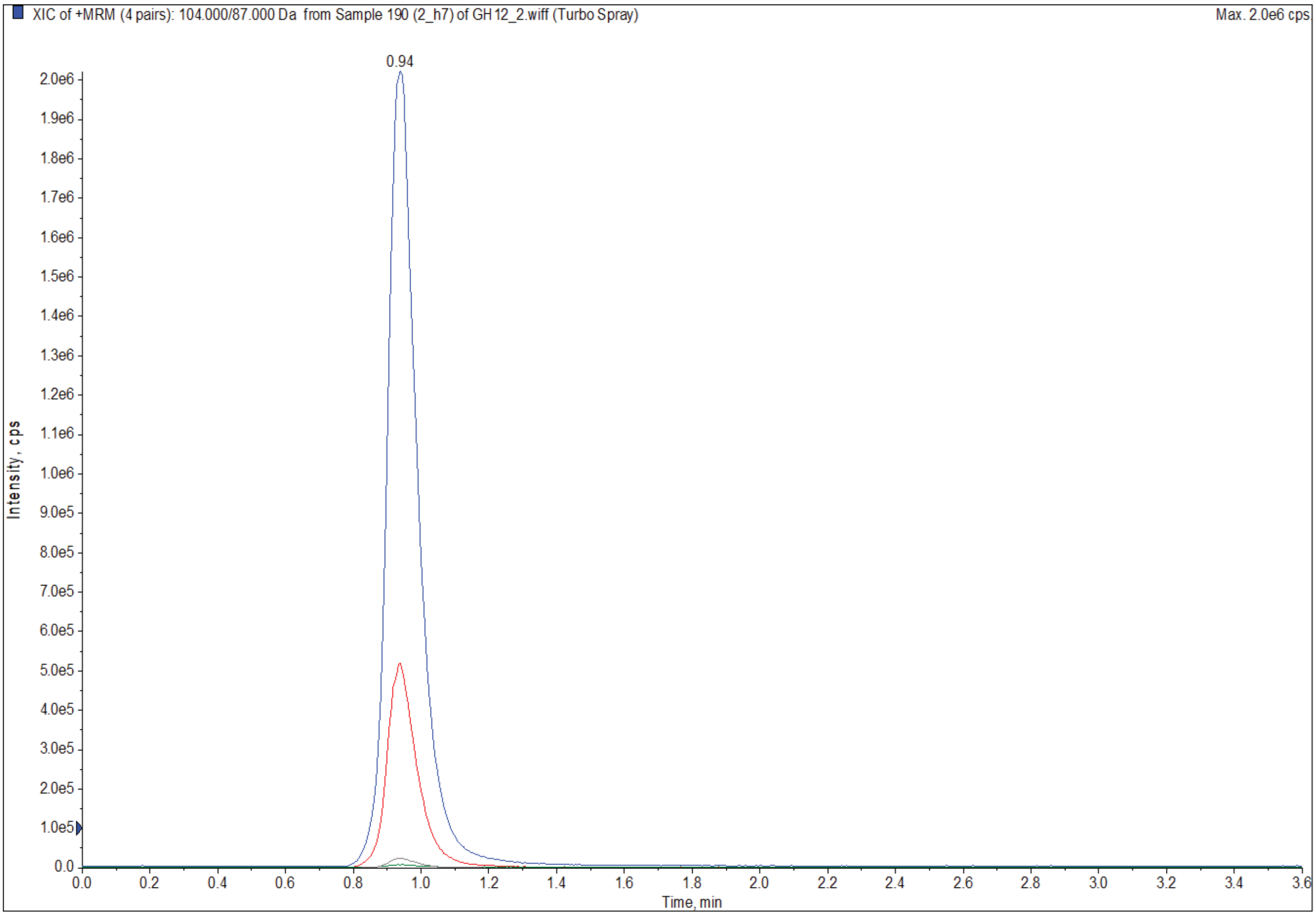

Fig S8l – 250 nM GABA
