# Supplementary material for: Cryo-EM structure of GABA transporter 1 reveals substrate recognition and transport mechanism
Source: Nat Struct Mol Biol. 2023 Jul 3;30(7):1023–32. doi: 10.1038/s41594-023-01011-w (PMC10352132; doi:10.1038/s41594-023-01011-w)
Supplement: Supplementary file 2 — Reporting Summary [file 41594_2023_1011_MOESM2_ESM.pdf]

nature portfolio

Corresponding author(s): Aravind Penmatsa

Last updated by author(s): Apr 9, 2023

Reporting Summary

Nature Portfolio wishes to improve the reproducibility of the work that we publish. This form provides structure for consistency and transparency in reporting. For further information on Nature Portfolio policies, see our Editorial Policies and the Editorial Policy Checklist.

Statistics

For all statistical analyses, confirm that the following items are present in the figure legend, table legend, main text, or Methods section.

n/a	Confirmed	
☐	☒	The exact sample size (*n*) for each experimental group/condition, given as a discrete number and unit of measurement
☐	☒	A statement on whether measurements were taken from distinct samples or whether the same sample was measured repeatedly
☒	☐	The statistical test(s) used AND whether they are one- or two-sided<br>*Only common tests should be described solely by name; describe more complex techniques in the Methods section.*
☒	☐	A description of all covariates tested
☒	☐	A description of any assumptions or corrections, such as tests of normality and adjustment for multiple comparisons
☐	☒	A full description of the statistical parameters including central tendency (e.g. means) or other basic estimates (e.g. regression coefficient) AND variation (e.g. standard deviation) or associated estimates of uncertainty (e.g. confidence intervals)
☒	☐	For null hypothesis testing, the test statistic (e.g. *F*, *t*, *r*) with confidence intervals, effect sizes, degrees of freedom and *P* value noted<br>*Give P values as exact values whenever suitable.*
☒	☐	For Bayesian analysis, information on the choice of priors and Markov chain Monte Carlo settings
☒	☐	For hierarchical and complex designs, identification of the appropriate level for tests and full reporting of outcomes
☒	☐	Estimates of effect sizes (e.g. Cohen's *d*, Pearson's *r*), indicating how they were calculated

Our web collection on statistics for biologists contains articles on many of the points above.

Software and code

Policy information about availability of computer code

Data collection	Cryo-EM data were collected through EPU 2.14.0 (eBIC, Diamond) using Ametek-Gatan Bio-quantum-K3 detector on a Titan Krios operating at 300keV.
Data analysis	CryoSPARC (version 3.0), GraphPad Prism (version 6.07; 5.01), Microsoft Excel (version 2210), Coot (version 0.9.6), UCSF Chimera (version 1.15), UCSF ChimeraX (version 1.4), Pymol (version 2.5.2) (Schrödinger), Gromacs (version 2020.4), ColabFold v1.5.2: AlphaFold2 using MMseqs2, phenix.real_space_refine (v1.20.1)

For manuscripts utilizing custom algorithms or software that are central to the research but not yet described in published literature, software must be made available to editors and reviewers. We strongly encourage code deposition in a community repository (e.g. GitHub). See the Nature Portfolio guidelines for submitting code & software for further information.

Data

Policy information about availability of data

All manuscripts must include a data availability statement. This statement should provide the following information, where applicable:

- Accession codes, unique identifiers, or web links for publicly available datasets
- A description of any restrictions on data availability
- For clinical datasets or third party data, please ensure that the statement adheres to our policy

The refined coordinates and maps of the rGAT1 structure in this study have been deposited in the PDB (PDB id 8GNK) and EMDB (EMD-34167). Raw data for

experiments is deposited along with the manuscript as a source data file and raw chromatograms for LC-ESI MS/MS data are provided in supplemental files. MD trajectories have been deposited at the following resource (https://osf.io/f9nr6/?view_only=a859d5ff464c404196eedb935373b0e3).

Human research participants

Policy information about studies involving human research participants and Sex and Gender in Research.

Reporting on sex and gender	Not Applicable
Population characteristics	Not Applicable
Recruitment	Not Applicable
Ethics oversight	Not Applicable

Note that full information on the approval of the study protocol must also be provided in the manuscript.

Field-specific reporting

Please select the one below that is the best fit for your research. If you are not sure, read the appropriate sections before making your selection.

☒ Life sciences ☐ Behavioural & social sciences ☐ Ecological, evolutionary & environmental sciences

For a reference copy of the document with all sections, see nature.com/documents/nr-reporting-summary-flat.pdf

Life sciences study design

All studies must disclose on these points even when the disclosure is negative.

Sample size	Biochemical experiments involving GABA uptake were done as two independent experiments each done in triplicate. All the six data points were used for calculation of biochemical parameters like KM and IC50. Binding studies using mass spectroscopy was done as four or three independent trials, each done in triplicate. One representative trial was used to report the dissociation constant for inhibitor. No sample size calculations were performed as the experiments done had very strong and consistent signal among biological replicates and had very low background values. Further no quantitative comparisons of constructs were done to necessitate t-tests of p-value estimates. These assays are performed with similar sample sizes in earlier studies as well.
Data exclusions	There are no data points that are excluded.
Replication	All the biochemical assays are performed in triplicates repeated two independent times as mentioned in the figure legends as well as in the methods section. Binding assays were also done in triplicate for each biological repeat and each experiement had three to four biological repeats.
Randomization	Samples were not randomized.
Blinding	Blinding is not applicable to the current study

Reporting for specific materials, systems and methods

We require information from authors about some types of materials, experimental systems and methods used in many studies. Here, indicate whether each material, system or method listed is relevant to your study. If you are not sure if a list item applies to your research, read the appropriate section before selecting a response.

Materials & experimental systems

n/a	Involved in the study	
☐	☒	Antibodies
☐	☒	Eukaryotic cell lines
☒	☐	Palaeontology and archaeology
☒	☐	Animals and other organisms
☒	☐	Clinical data
☒	☐	Dual use research of concern

Methods

n/a	Involved in the study	
☒	☐	ChIP-seq
☒	☐	Flow cytometry
☒	☐	MRI-based neuroimaging

Antibodies

Antibodies used	The fragment antigen binding (Fab) used in the current study is a recombinant version of Fab used in https://www.nature.com/articles/nature12533/. The methodology of heterologous expression and purification of Fab is detailed in the methods section and adopted from the previous study https://www.nature.com/articles/s41467-021-22385-9.
Validation	The activity of the recombinant Fab was validated by checking its ability to bind to the target protein and measuring the shifts in chromatogram with Fab bound and unbound protein as shown in figure 1C of the manuscript and eventually elucidating the structure of rGAT1-Fab complex.

Eukaryotic cell lines

Policy information about cell lines and Sex and Gender in Research

Cell line source(s)	HEK293SGnTI- cell lines from ATCC (CRL3022), HEK293 cells were from Leibniz Institute DSMZ-German Collection of Microorganisms and Cell Cultures, Braunschweig, Germany and SF9 cells from Gibco (11496015)
Authentication	No further authentication was performed after receipt.
Mycoplasma contamination	Not tested for mycoplasma contamination
Commonly misidentified lines (See ICLAC register)	The above cell lines are no commonly misidentified
