# Supplementary figures and images for: Cryo-EM structure of GABA transporter 1 reveals substrate recognition and transport mechanism
Source: Nat Struct Mol Biol. 2023 Jul 3;30(7):1023–32. doi: 10.1038/s41594-023-01011-w (PMC10352132; doi:10.1038/s41594-023-01011-w)

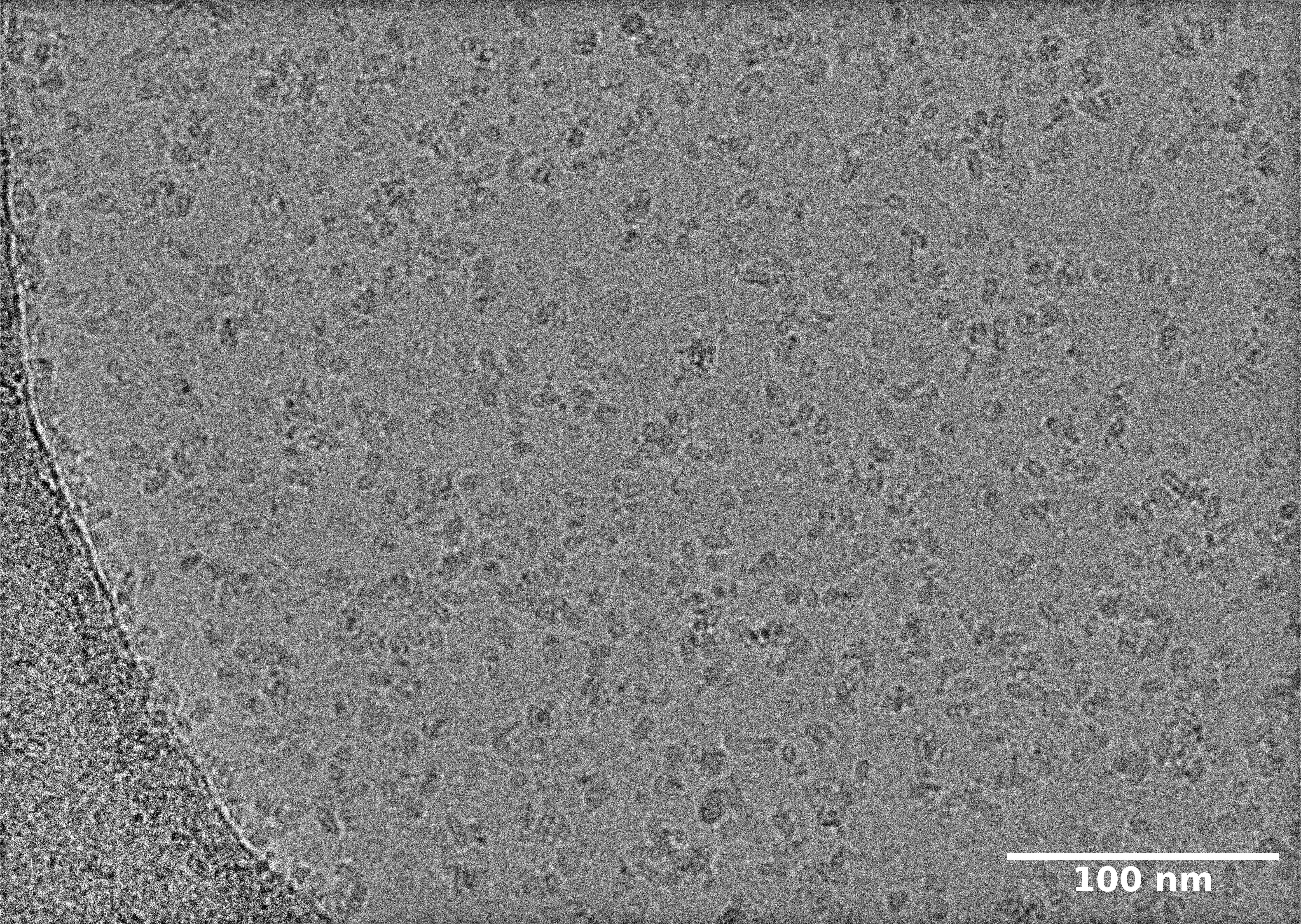

Supplement: Supplementary file 4 — Source Data Extended Data Fig. 4 contains a representative cryo-EM micrograph image, FSC curve image, rGAT1EM coordinates file and the rGAT1WT AlphaFold model file. [file 41594_2023_1011_MOESM4_ESM.zip › SourceData ExtendedDataFig4/ED-Fig.4b.jpeg]

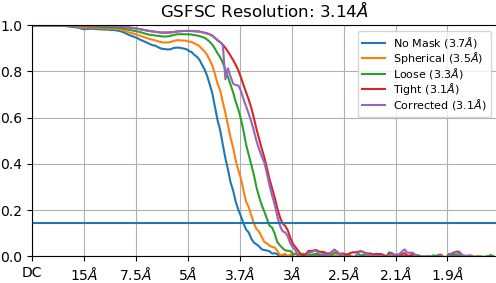

Supplement: Supplementary file 4 — Source Data Extended Data Fig. 4 contains a representative cryo-EM micrograph image, FSC curve image, rGAT1EM coordinates file and the rGAT1WT AlphaFold model file. [file 41594_2023_1011_MOESM4_ESM.zip › SourceData ExtendedDataFig4/ED-Fig4e.png]

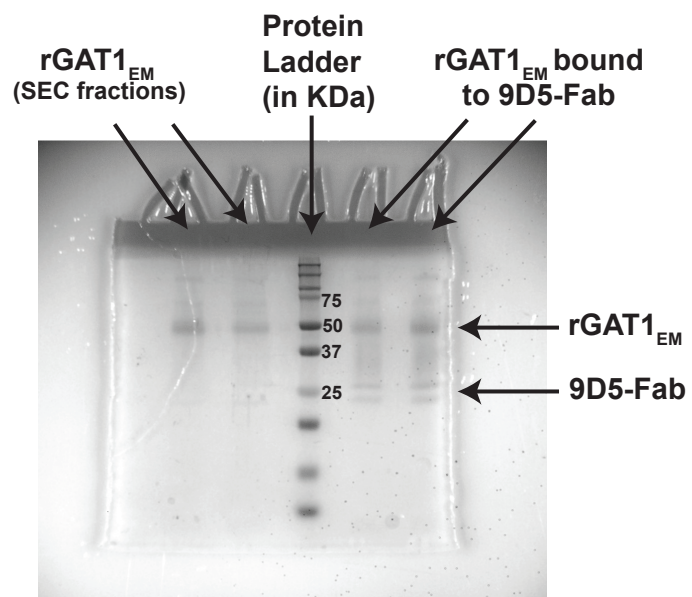

Fig ED 3a

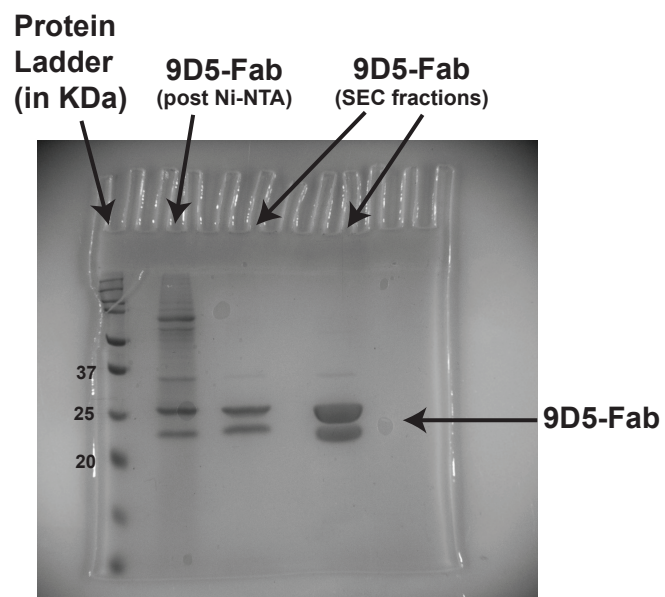

Fig ED 3b

Supplement: Source Data Extended Data Fig. 3 — Fig_ED3_gel_image has two images. Fig_ED3a contains a gel image of purified rGAT1EM. Fig_ED3b_data contains a gel image of purified 9D5. [file 41594_2023_1011_MOESM9_ESM.pdf]
