# Supplementary material for: Cryo-EM structure of GABA transporter 1 reveals substrate recognition and transport mechanism
Source: Nat Struct Mol Biol. 2023 Jul 3;30(7):1023–32. doi: 10.1038/s41594-023-01011-w (PMC10352132; doi:10.1038/s41594-023-01011-w)
Supplement: Supplementary file 3 — Peer Review File [file 41594_2023_1011_MOESM3_ESM.docx]

Peer Review Information

**Journal:** Nature Structural & Molecular Biology

**Manuscript Title:** Cryo-EM structure of GABA transporter 1 reveals substrate recognition and transport mechanism

**Corresponding author name(s):** Aravind Penmatsa

**Reviewer Comments & Decisions:**

**Decision Letter, initial version:**

| **Message:** | 18th Oct 2022  Dear Aravind,  Thank you again for submitting your manuscript "Cryo-EM structure of GABA transporter 1 reveals substrate recognition and transport mechanism". We now have comments (below) from the 2 reviewers who evaluated your paper. In light of those reports, we remain interested in your study and would like to see your response to the comments of the referees, in the form of a revised manuscript. Please be sure to address/respond to all concerns of the referees in full in a point-by-point response and highlight all changes in the revised manuscript text file. If you have comments that are intended for editors only, please include those in a separate cover letter.  We are committed to providing a fair and constructive peer-review process. Do not hesitate to contact us if there are specific requests from the reviewers that you believe are technically impossible or unlikely to yield a meaningful outcome.  We expect to see your revised manuscript within 6 weeks. If you cannot send it within this time, please contact us to discuss an extension; we would still consider your revision, provided that no similar work has been accepted for publication at NSMB or published elsewhere.  As you already know, we put great emphasis on ensuring that the methods and statistics reported in our papers are correct and accurate. As such, if there are any changes that should be reported, please submit an updated version of the Reporting Summary along with your revision.  Please follow the links below to download these files:  Reporting Summary: https://www.nature.com/documents/nr-reporting-summary.pdf  Please note that the form is a dynamic ‘smart pdf’ and must therefore be downloaded and completed in Adobe Reader.   When submitting the revised version of your manuscript, please pay close attention to our href="https://www.nature.com/nature-portfolio/editorial-policies/image-integrity">Digital Image Integrity Guidelines.</a>  Finally, please ensure that you retain unprocessed data and metadata files after publication, ideally archiving data in perpetuity, as these may be requested during the peer review and production process or after publication if any issues arise.  If there are additional or modified structures presented in the final revision, please submit the corresponding PDB validation reports.  SOURCE DATA: we urge authors to provide, in tabular form, the data underlying the graphical representations used in figures. This is to further increase transparency in data reporting, as detailed in this editorial (http://www.nature.com/nsmb/journal/v22/n10/full/nsmb.3110.html). Spreadsheets can be submitted in excel format. Only one (1) file per figure is permitted; thus, for multi-paneled figures, the source data for each panel should be clearly labeled in the Excel file; alternately the data can be provided as multiple, clearly labeled sheets in an Excel file. When submitting files, the title field should indicate which figure the source data pertains to. We encourage our authors to provide source data at the revision stage, so that they are part of the peer-review process.  Data availability: this journal strongly supports public availability of data. All data used in accepted papers should be available via a public data repository, or alternatively, as Supplementary Information. If data can only be shared on request, please explain why in your Data Availability Statement, and also in the correspondence with your editor. Please note that for some data types, deposition in a public repository is mandatory - more information on our data deposition policies and available repositories can be found below: https://www.nature.com/nature-research/editorial-policies/reporting-standards#availability-of-data  We require deposition of coordinates (and, in the case of crystal structures, structure factors) into the Protein Data Bank with the designation of immediate release upon publication (HPUB). Electron microscopy-derived density maps and coordinate data must be deposited in EMDB and released upon publication. Deposition and immediate release of NMR chemical shift assignments are highly encouraged. Deposition of deep sequencing and microarray data is mandatory, and the datasets must be released prior to or upon publication. To avoid delays in publication, dataset accession numbers must be supplied with the final accepted manuscript and appropriate release dates must be indicated at the galley proof stage.  While we encourage the use of color in preparing figures, please note that this will incur a charge to partially defray the cost of printing. Information about color charges can be found at http://www.nature.com/nsmb/authors/submit/index.html#costs  Nature Structural & Molecular Biology is committed to improving transparency in authorship. As part of our efforts in this direction, we are now requesting that all authors identified as ‘corresponding author’ on published papers create and link their Open Researcher and Contributor Identifier (ORCID) with their account on the Manuscript Tracking System (MTS), prior to acceptance. This applies to primary research papers only. ORCID helps the scientific community achieve unambiguous attribution of all scholarly contributions. You can create and link your ORCID from the home page of the MTS by clicking on ‘Modify my Springer Nature account’. For more information please visit please visit <a href="http://www.springernature.com/orcid">www.springernature.com/orcid</a>.  Please use the link below to submit your revised manuscript and related files:  [Redacted]  <strong>Note:</strong> This URL links to your confidential home page and associated information about manuscripts you may have submitted, or that you are reviewing for us. If you wish to forward this email to co-authors, please delete the link to your homepage.  We look forward to seeing the revised manuscript and thank you for the opportunity to review your work.  Kind regards, Florian  Dr Florian Ullrich Associate Editor, Nature Consulting Editor, Nature Structural & Molecular Biology ORCID 0000-0002-1153-2040    Referee expertise:  Referee #1: cryo-EM, transporters  Referee #2: transporter structure-function   Reviewers' Comments:  Reviewer #1: Remarks to the Author: The GABA transporter GAT1 terminates GABA signaling by electrogenic transport of neurotransmitter GABA in the central nervous system. GAT1 couples the uphill flux of substrate to co-transport of sodium and chloride ions. The manuscript “Cryo-EM structure of GABA transporter 1 reveals substrate recognition and transport mechanism” by SR. Nayad and D. Joseph et al. describes the structure of Rattus norvegicus GAT1 in an inward-facing state. The structure determination has been facilitated by a dDAT Fab molecule where the interaction site has been engineered in GAT1. There is no doubt that this study is significant with relevant findings. There are however, several concerns as described below.  I have a major issue with GABA binding mode. I cannot find any other structure of bacterial or human homologues of GAT1 that would support the binding mode of GABA in this structure. The site is very conserved, and the authors also refer to their previous study (ref. 22), but in that study as well, the carboxylate group of the inhibitors form the expected interactions and bind similar to amino acid/inhibitors bound structures of homologue transporters (see e.g., MhsT, LeuT structures with amino acids, GlyT1 with inhibitor, and dDAT like GAT1 structures in ref. 22). There should be stronger evidence that the density belongs to GABA in that certain biding mode. I am aware that the state is perhaps an intermediate state, but it is unlikely to trap GABA bound in that mode. I suggest to use computation/simulation methods to validate the binding mode.  In the abstract, discussion and main text, the turn in TM10 is referred to a 3-residue insertion in TM10 in GATs and BGT1, which is actually a one-residue insertion compared to other NSS, e.g., SERT, DAT, NET, GlyT1 and LeuT, and this extra flexibility in TM10 is also seen in other NSS structures. The insertion in the TM10 helix is rather the second Serine residue in the sequence YYASSG, as other residues are conserved among NSS (see e.g., the review: Structural and molecular aspects of betaine-GABA transporter 1 (BGT1) and its relation to brain function, Kickinger et al., Neuropharmacology, 2019. Please modify the discussion accordingly.  In Page 4, “…rGAT1 displays direct high-affinity interaction with inhibitors in cell membranes whereas the detergent solubilized transporter does not show…”, please provide the reference. (Or has it been concluded from an experiment performed by the authors? If so, please provide a figure and add the details in the methods section).  Page 6, “… the H-bond network in this region is more compact than similar structures observed for hSERT and hGlyT1, likely facilitating…” There are a few issues with this argument: based on what study it is concluded that formation of inward-facing state is easier in GATs? are there any kinetics supporting this statement? Also, the structures referred to of GlyT1 and SERT are bound to bulky inhibitors and difference in H-bond network is expected. Further, how does it compare to hGAT1 structure? Please provide a figure of H-bond network comparison as an extended data figure.  There are several minor comments which might be helpful for the authors to improve their manuscript.  Please spell out BGT1 as Betaine/GABA transporter BGT1  Page 4, paragraph 2, facilitated by the interactions with TM10… it is not clear what is interacting with what  Page 5, If the disulfide bridge between C164 and C173 is the conserved one in the NSS, please mention it in the text or figure legend.  Page 6, correct the mutation “D451” to D451E.  Page 7, … “However, the sodium site appears displaced from its traditional coordinating site between TM1 and TM6”. Please add an ED figure comparing the conventional and displaced Na binding site  Figures:  Figure 1. legends e and f, are difficult to understand, please mention the number of independent experiments and technical replicates, clearly. In figures presenting these and similar plots, please show individual data points and error bars. The reviewer suggests to show the average of technical replicates in independent experiments, as individual data points (so, if there are 3 independent experiments, there will be 3 data points).  Figure 4d, please add a line on how the distance between the ligand and membrane has been measured (could be the center of mass of the ligand).  Please mention the Local resolution estimate where ions, water molecule and ligand are discussed. This could be added to the respective figure legends.  Please show the density where residue rotamers or interactions in the binding pocket are discussed e.g., in figure 3 and figure 2 d, e, or as an extended data figure. (Density for the residues such as Y60, F294, Y140 in their respective figures or as an extended data figure).  In ED Figure 7 b, color T400 in blue  Please add hGAT1 to sequence alignment in Fig. S1.  In the methods section:  - Purification of Fab: Large volume of Sf9 cells were infected with high titer of virus…. Please be more precise. What was the MOI used? How much cells at what concentration where infected, etc  - GABA uptake and inhibition assay Please provide details on uptake/inhibition data processing, calculation of Km, Vmax, etc e.g., data were fit to which equation Please mention the number of independent experiments.  - MS Binding assay Please specify the concentrations used in the 2.5 nM to 1.28 uM and 40 nM to 1.28 uM ranges.    Reviewer #2: Remarks to the Author: The GAT1 structure presented in this manuscript raises several interesting questions. One of the most obvious is the fact that a structure obtained in the presence of Na+ and substrate was found to be in an inward-open conformation. Structures of the related transporters LeuT, dDAT and SERT obtained under similar conditions were all outward-facing, either occluded-out or open-out. The authors speculate that this could result from the insertion of three residues in TM10. It would be appropriate to expand on this, at least to describe which, if any, other aspects of the structure could account for the difference in conformation.  There is also a brief discussion of the effects of extracellular pathway mutations on transport activity and Na+ binding (page 6). Is it possible to relate the behavior of GAT1 in oocytes, for example, with its conformation? Is there a step preceding extracellular Na+ binding that is detectible (by a transient current, for example) that could correspond to a conformational transition from the inward- to outward-facing conformation? Such an observation could help to validate the inward-facing structure shown here and to relate it to the behavior of GAT1 in a cell membrane.  The lack of Na+ in the Na2 site is consistent with the open cytoplasmic pathway. What was the Na+ concentration in the sample prep? All I can gather is that it was between 50 mM in the antibody prep and 300 mM for the transporter. Is it possible that an increase in [Na+] would fill the Na2 site and close the pathway? Was this ever tested? The possibility should be discussed.  Could the differences in KM and inhibitor affinity between GAT1WT and GAT1EM be due to a conformational effect of mutation? Does binding of the antibody influence these values? If it did, and this would be easy to measure, it would explain the difference in affinity or KM, but also would present a problem for interpreting this structure as a native one.  Because GABA is a γ-amino acid, unlike α-amino acids that are transported by most members of this family, the coordination of the amino group is likely to differ. For example, in LeuT Ser256, which corresponds to Gly297 in GAT1. It would be useful to compare the interactions between substrate and coordinating residues in this structure with those of LeuT or MhsT.  The authors describe “movement” between this structure and an outward-facing structure. No outward-facing GAT-1 structure is available, and I don’t think the structure of Drosophila DAT with binding site residues changed to those of GAT1 is a good basis for a model. Did the author make a model based on LeuT or other members of the family? There are many places in the manuscript where such movements are implied without a point of reference. Without at least a model of outward-facing GAT1, these statements are hard to accept and should be either explained better or removed. There are some places in the manuscript where the authors describe a sequence of events (for example in the section “Opening of cytosolic gates…”) where they describe a sequence of events that transform the transporter from an unknown conformation to the structure they present. It is important to describe this process as speculation, not as observation. All we have is this structure and a proposed closed structure and the conversion between the two is not an experimental finding but rather the authors interpretation and should be presented as such and not stated as fact.  There are several places in the manuscript where this problem shows up. I am relying on the authors to correct them all. There is a place for speculation, but it needs to be presented as such. One observation that seems an interesting possibility for speculation is the interaction between the substrate carboxyl group, Na+, and Tyr140. In LeuT structures, the carboxyl group bridges the corresponding TM3 Tyr108 and Na+ bound at Na1. Here, the GABA carboxyl group is still coordinated with both Tyr104 and Na+, but it is no longer bridging the two, which directly coordinate with each other. However, the Na+ ion is further from Cl- than in structures of other members of this family, suggesting that it has started to move, together with substrate, towards the open cytoplasmic pathway. The distance between Na+ and Cl- in the other structures is about 6 Å or less but in this structure, they are 8 Å apart.  Why compare GABA binding site with that of biogenic amine transporters? Aren’t LeuT or other amino acid transporters better models? If you compare amino acids bound in LeuT with 5-HT bound to SERT, the differences are profound.  Minor points: Page 3, The rocking bundle hypothesis was an early and simplistic mechanism. It is now quite clear that the bundle (not rocking bundle) helices (1,2,6 and 7) pivot from the cytoplasmic face of the transporter to open and close the extracellular pathway but the cytoplasmic pathway opens by the movement of TM1a, which pivots from the center of the transporter to swing away from the rest of the bundle and open the pathway. In other words, the bundle does not rock to open the cytoplasmic pathway. The manuscript uses words like above and below to indicate position towards the extracellular or cytoplasmic surface. This needs to be clearly stated to avoid confusion. Page 3, The ibogaine binding site overlaps with the substrate binding site of SERT. My impression is that the same is true for tiagabine. It is a misstatement that the binding sites are below or beneath the substrate site. Page 5, What is the evidence that the first 38 residues of GAT1 are disordered? Page 5, KM is not affinity or apparent affinity. It is a kinetic constant that, only under some special conditions can equal the KD for substrate. Page 7, Instead of referring to the position of bound Na+ as “traditional”, it might be more useful to describe which residues it interacts with in other transporters or the distance from the bound Cl-. Page 7, Reference 13 is cited for LeuT. It is actually a reference to SERT. Page 8, The term “linker” for the unwound (or non-helical) region of TM1 or TM6 is confusing. Please use a more descriptive name. Page 8, Chloride is required for many transporters in this family. It’s role has been proposed to involve formation of the TM1-TM10 salt bridge, but there is no specific relationship between the charge on Cl- and GABA transport outside of that or the electrostatic interaction with Na+. Page 9, There are many LeuT structures where Y108, which corresponds to GAT1 Y140, interacts with the substrate carboxyl group, and it would be surprising if the GABA carboxyl did not coordinate Y140 in GAT1 in the outward-occluded or -open conformations. The statement in the middle of the first paragraph on page 9 should be modified so it doesn’t give the impression that the two do not interact in other states of the complex. Page 9, 2/3 from the top: …50 to 60. TM1a, in… …where TM1a is closer to TM7,… …N-terminus. TM1a could…position through the transport cycle. The corresponding residue, Y60, Page 10, 3rd line of discussion, …targets is a lengthy and resource… Page 11, middle, …site 2 is proposed to initiate the release of… Page 11, …that were first observed on the cytoplasmic face…  Reviewed by Gary Rudnick |
| --- | --- |

**Author Rebuttal to Initial comments**

**Responses to Reviewers**

Reviewer #1:
Remarks to the Author:
The GABA transporter GAT1 terminates GABA signaling by electrogenic transport of neurotransmitter GABA in the central nervous system. GAT1 couples the uphill flux of substrate to co-transport of sodium and chloride ions. The manuscript “Cryo-EM structure of GABA transporter 1 reveals substrate recognition and transport mechanism” by SR. Nayad and D. Joseph et al. describes the structure of Rattus norvegicus GAT1 in an inward-facing state. The structure determination has been facilitated by a dDAT Fab molecule where the interaction site has been engineered in GAT1. There is no doubt that this study is significant with relevant findings. There are however, several concerns as described below.

**Response: We thank the reviewer for providing constructive comments about our study and finding this study to be significant with relevant findings. We have tried to address all of his/her concerns in the responses below**.

I have a major issue with GABA binding mode. I cannot find any other structure of bacterial or human homologues of GAT1 that would support the binding mode of GABA in this structure. The site is very conserved, and the authors also refer to their previous study (ref. 22), but in that study as well, the carboxylate group of the inhibitors form the expected interactions and bind similar to amino acid/inhibitors bound structures of homologue transporters (see e.g., MhsT, LeuT structures with amino acids, GlyT1 with inhibitor, and dDAT like GAT1 structures in ref. 22).

**Response: The GABA binding mode was modeled as per the visualized density in the binding pocket. While the carboxylate is in proximity to the Tyr140 in the structure, it is not within the H-bond forming distance. We have further elaborated on this point in the later comments.**

There should be stronger evidence that the density belongs to GABA in that certain biding mode. I am aware that the state is perhaps an intermediate state, but it is unlikely to trap GABA bound in that mode. I suggest to use computation/simulation methods to validate the binding mode.

**Response: The structure we deposited has a clear density that fits GABA in the primary binding site. We also provided detailed analyses of GABA presence in HEK293 cells and also presence of GAD1 transcripts in HEK293 cells that could be a source of GABA for rGAT1. We do however understand the concern of the reviewer and have carried out multiple all-atom simulations of the GABA bound GAT1 in a POPC:POPE:Chol bilayer for 100 ns (Extended Data Fig. 9).**

**The following statement in the text, page 10, summarizes the simulation data, “We performed molecular dynamics simulations of zwitterionic GABA bound rGAT1 to evaluate it’s stability in the binding pocket. During the simulation, we could observe GABA carboxylate shifting closer to subsite A to form H-bond interactions with hydroxyl of Y140. It should be noted that the simulations have been performed in a lipid bilayer, where these transporters are expected to be dynamic. Despite this, GABA remained relatively stable in the binding pocket. In the simulations, the proximity of GABA carboxylate with Y140 prevents interactions of GABA amine group with A455 carbonyl and interacts with surrounding residues, particularly with the side chain of T400. Consequently, Y140F substitution led to a weakened GABA binding network, with GABA being extremely flexible or moving away from the binding pocket in the simulation time scales (Extended Data. Fig. 9a-f).”**

In the abstract, discussion and main text, the turn in TM10 is referred to a 3-residue insertion in TM10 in GATs and BGT1, which is actually a one-residue insertion compared to other NSS, e.g., SERT, DAT, NET, GlyT1 and LeuT, and this extra flexibility in TM10 is also seen in other NSS structures. The insertion in the TM10 helix is rather the second Serine residue in the sequence YYASSG, as other residues are conserved among NSS (see e.g., the review: Structural and molecular aspects of betaine-GABA transporter 1 (BGT1) and its relation to brain function, Kickinger et al., Neuropharmacology, 2019. Please modify the discussion accordingly.

**Response: The reviewer is right in pointing this out. We have now modified the text as follows to address the presence of a single residue insertion and cited two references by Dayan et al 2017 and Kickinger et al 2019 to suggest the presence of either S456 or G457 as an additional insertion that leads to the formation of a π-helix in TM10.**

**The text in page 6 is modified as follows, “TM10 has a single residue insertion, with either S456 or G457 depending on the alignment, unique to the GABA, taurine and creatine transporters at the centre of the bilayer^26,27^ that induces the widening of the helix to become a one turn π-helix^26^. Substitutions at this insertion are documented to hamper the uptake kinetics of GABA^26^. The side chain hydroxyl of the S456 in TM10 interacts with T290 hydroxyl in TM6 (3.4 Å) (Fig. 2d, inset) to enhance the closure of the extracellular gate. The interactions are similar to recently determined hGAT1 structure^21^, although the side chain positions and H-bond distances are modeled with greater precision in rGAT1 due to the improved resolution (Extended Data Fig. 6a and b).”**

In Page 4, “…rGAT1 displays direct high-affinity interaction with inhibitors in cell membranes whereas the detergent solubilized transporter does not show…”, please provide the reference. (Or has it been concluded from an experiment performed by the authors? If so, please provide a figure and add the details in the methods section).

**Response: The above statement was made in the light of ^3^H-tiagabine binding assays done using solubilized rGAT1 that displayed no counts in comparison the background despite the high reported affinity for GAT1 (figure below). We however do not have a clear positive control for these measurements and have therefore removed this statement from the text and rephrased the lines in the introduction. “The modified rGAT1 retains GABA uptake activity and displays direct high-affinity interaction with a GAT1 inhibitor, NO711, in cell membranes. The presence of the detergent solubilized transporter in the cytosol-facing state suggests the possibility of a conformational reorganization of the transporter during extraction from the plasma membrane.”**

Page 6, “… the H-bond network in this region is more compact than similar structures observed for hSERT and hGlyT1, likely facilitating…”
There are a few issues with this argument: based on what study it is concluded that formation of inward-facing state is easier in GATs? are there any kinetics supporting this statement?

**Response: The H-bond network within the extracellular gate is clearly more extensive in rGAT1 and hGAT1 structures in comparison to the hSERT, LeuT and hGlyT1 inward-open structures. This is stated both from the closer H-bond distances and also from the additional H-bond interactions induced by the insertion at TM10. The functional data were generated by investigating transporters with specific substitutions in the gating residues R69, Q291 and D451 that drastically affect the uptake activity and conformational equilibrium of the transporter as documented in references 28-30. We have also added a comparative figure to display H-bond network differences among inward open states of rGAT1, hGAT1, hGlyT1, hSERT and LeuT in Extended data fig. 6.**

**The following paragraph in page 6 summarizes the role of H-bond network in driving conformational changes.**

**“Disrupting residues vital for gating at the extracellular side through substitutions like R69K lead to compromised GABA uptake and most substitutions result in the loss of uptake activity in GAT1 suggesting their importance in ion-coupled GABA transport^28-30^. R69K, Q291N and D451E have been analyzed by using the two-electrode-voltage clamp methodology. Even though these conservative replacement mutants have impaired transport, they all exhibit capacitative transient currents^28-30^, reflecting the transition between empty-inward and empty-outward conformations, preceding the binding of extra-cellular sodium, which stabilizes the outward conformation^30,31^. The voltage dependence of transient currents by GAT1_WT_ indicates that under physiological conditions a significant proportion of the transporters is inward-open. In the mutants this voltage dependence is markedly left-shifted relative to those by rGAT1_WT_ indicating a much larger proportion of outward-open transporters, apparently as a result of a perturbed extracellular gate^28-30^. Therefore these transporters are “stuck” in an outward-open conformation and cannot cycle through inward-open conformations, resulting in impaired transport.”**

Also, the structures referred to of GlyT1 and SERT are bound to bulky inhibitors and difference in H-bond network is expected. Further, how does it compare to hGAT1 structure?
Please provide a figure of H-bond network comparison as an extended data figure.

**Response: A new ED figure 6 is now added to compare the H-bond networks in rGAT1 and hGAT1. We further provide the extracellular gate network in cytosol facing structures of hSERT, hGlyT1 and LeuT to provide a direct comparison. The bulky inhibitors, ibogaine and cmpd1 are bound towards the cytosolic gate of hSERT and hGlyT1, respectively and may not influence the H-bond interactions of the closed extracellular gate.**

There are several minor comments, which might be helpful for the authors to improve their manuscript.

Please spell out BGT1 as Betaine/GABA transporter BGT1
**Response: We have spelt this out in paragraph 1 of the introduction.**

Page 4, paragraph 2, facilitated by the interactions with TM10… it is not clear what is interacting with what

**Response: The paragraph forms part of the introduction where we mention the formation of a closed extracellular gate through H-bond interactions. The details of interactions are provided in a later section in page 6. We edited this line in the introduction as follows, “The rGAT1 structure displays a closed extracellular gate facilitated by the interactions between residues from the bundle of gating helices and the scaffold helices with a solvent accessible substrate-binding site facing the cytosol”.**

Page 5, If the disulfide bridge between C164 and C173 is the conserved one in the NSS, please mention it in the text or figure legend.

**Response: We have now highlighted the disulfide bond density as an inset in ED figure 5 and also mention “conserved” in the text (top of page 6) and legend.**

Page 6, correct the mutation “D451” to D451E.

**Response: D451E typo is corrected.**

Page 7, … “However, the sodium site appears displaced from its traditional coordinating site between TM1 and TM6”. Please add an ED figure comparing the conventional and displaced Na binding site
**Response: We have added a new panel ED. figure 8 (panel b) to show the displaced sodium from site1. The panel also describes the subsite organization within GAT1.**

Figure 1. legends e and f, are difficult to understand, please mention the number of independent experiments and technical replicates, clearly.

**Response: Both legends are now edited with details of independent experiments and technical replicates as suggested by the reviewer. The uptake kinetics and uptake inhibition experiments were done as two independent experiments each done in 3 technical replicates. The mass spectrometry-binding assay was done as multiple independent experiments each with three replicates. One representative trial is shown in Fig. 1g and ED. Fig. 2c respectively. The raw data for all four (rGAT1_EM_)/three (rGAT1_WT_) experiments is shown in figures S3 and S4. The legend is modified as follows, “e, ^3^H-GABA uptake assays were performed in two independent measurements each done in triplicate. Each point representing a mean of six measurements and error bars representing sem. The two independent datasets are displayed as Fig. S3 and S4. The *K*_M_ and *V*_max_ values are 11 μM and 2023 fmol/well/min for rGAT1_WT_ and 4.2 μM and 855.7 fmol/well/min for rGAT1_EM_. f, Measurement of tiagabine and NO711 inhibition potency against rGAT1_EM_ indicates an IC50 of 704 nM and 154.9 nM respectively. Uptake measurements were done as two independent experiments each performed in triplicate. Each data point in the graph represents an average of all six measurements with error bars representing SEM. g, Binding measurements of rGAT1_EM_ expressing membranes with NO711, a tiagabine analogue. The affinity (Kd) displayed by the interaction is 80 nM in comparison to 50 nM for rGAT1_WT_. Data points represent specific binding (means ± SD, calculated from total and non-specific binding measured by LC-MS shown in Fig.S3, S4.) of one trial of four (rGAT1_EM_)/ three (rGAT1_WT_) independent experiments, each performed in triplicate.**

In figures presenting these and similar plots, please show individual data points and error bars. The reviewer suggests to show the average of technical replicates in independent experiments, as individual data points (so, if there are 3 independent experiments, there will be 3 data points).

**Response: The legends are modified to reflect the number of independent experiments done to obtain biochemical information, as detailed above. FigS3 and Fig S4 are added as per the suggestion of the reviewer to distinguish independent experiments for measuring *K*_M_, IC50 and binding affinities. The uptake kinetics and uptake inhibition experiments were done as two independent experiments each done in 3 technical replicates. The binding assay done using mass spectrometry were done as multiple independent experiments each with three replicates. One representative trial is shown in Fig. 1g and ED. Fig. 2c, respectively displayed below.**

Figure 4d, please add a line on how the distance between the ligand and membrane has been measured (could be the center of mass of the ligand).

**Response: The legend for Figure 4d is modified as follows.** **“The center of mass of the ligands GABA, tiagabine, NO711, Cmpd1 and ibogaine respectively were calculated in Pymol and were used to measure the distance of the ligands from the surface of a predicted membrane bilayer. rGAT1_EM_ structure was inserted into a membrane bilayer containing cholesterol, POPC and POPE lipids. The head groups of lipids in the lower leaflet provided the z-axis coordinates to calculate the distance of the centre of mass of ligands from the bottom of the membrane.”**

Please mention the Local resolution estimate where ions, water molecule and ligand are discussed. This could be added to the respective figure legends.
**Response: We have included the region around the substrate and sodium ion in the primary binding pocket as an inset in the ED figure 4d. The local resolution estimates indicate a resolution range of 2.7-2.9Å. Legend for the figure has been modified accordingly as follows, “Insets show the binding pocket with GABA and sodium densities coloured as per local resolution in both top and bottom views reflecting a resolution in the range of 2.7-2.9 Å.”**

Please show the density where residue rotamers or interactions in the binding pocket are discussed e.g., in figure 3 and figure 2 d, e, or as an extended data figure. (Density for the residues such as Y60, F294, Y140 in their respective figures or as an extended data figure).

**Response: The density of the three residues and residues surrounding the binding pocket have been added in the following figures as mentioned by the reviewers**

**Figure 2e inset (rotamers for F293, F294 (TM6) and Y140 (TM3); Figure 4a inset (Y60, TM1a); ED figure 8F (binding site residues).**

In ED Figure 7 b, color T400 in blue

**Response: ED7 is now ED8. The T400 is part of TM8 that is yellow in color. The blue in the figure (panel b) represents TM3. We have now incorporated the TM8 (in yellow, panel b)) to clarify the color in the image panel. The T400 remains in yellow color as before.**

Please add hGAT1 to sequence alignment in Fig. S1.
**Response: The hGAT1 sequence is added to the alignment in Fig. S1.**

In the methods section:

- Purification of Fab: Large volume of Sf9 cells were infected with high titer of virus….
Please be more precise. What was the MOI used? How much cells at what concentration where infected, etc
**Response: Details of the cell numbers, volume and procedure of infection are now included in the methods. The following are the changes made in the relevant method section.**

**“Due to the absence of GFP in the construct, volume of virus used for infection of SF9 cells was optimized based on small scale infections at different volume ratios of virus:SF9 culture volume and protein yield was observed as shifts in the FSEC peak of target transporter tagged to GFP induced by 9D5 in the supernatant of small scale cultures. Based on these estimates 10-15 ml of P3 recombinant baculovirus was added to 2 Erlenmeyer tissue culture flasks each with 800ml of SF9 cells at a cell density of 2.2 million/ml.”**

- GABA uptake and inhibition assay
Please provide details on uptake/inhibition data processing, calculation of Km, Vmax, etc e.g., data were fit to which equation
Please mention the number of independent experiments.
**Response: Appropriate changes have been made to the methods section. The software used to analyze uptake (Michaelis-Menten) and Ki calculation (Cheng-Prusoff) have been added to the method section. We also clearly indicated that each of the plots are data from two independent measurements containing three technical replicates.”**

- MS Binding assay
Please specify the concentrations used in the 2.5 nM to 1.28 uM and 40 nM to 1.28 uM ranges.
**Response: The individual concentrations for the data points have been included in the manuscript (Methods: MS Binding Assay).**


Reviewer #2:
Remarks to the Author:
The GAT1 structure presented in this manuscript raises several interesting questions. One of the most obvious is the fact that a structure obtained in the presence of Na+ and substrate was found to be in an inward-open conformation. Structures of the related transporters LeuT, dDAT and SERT obtained under similar conditions were all outward-facing, either occluded-out or open-out. The authors speculate that this could result from the insertion of three residues in TM10. It would be appropriate to expand on this, at least to describe which, if any, other aspects of the structure could account for the difference in conformation.

**We thank the reviewer for finding this study to be interesting and providing multiple suggestions that have improved the manuscript. The reviewer is right in pointing out that structures of dDAT, hSERT crystallized in excess sodium yielded outward-open structures.**

**Response: We discuss/elaborate this point as a paragraph in the discussion on page 12 as follows.**

**“It is noteworthy that structures of the related transporters dDAT, hSERT and LeuT obtained under similar conditions were all outward-open or outward-occluded states ^11-13^. In both dDAT and hSERT, extensive thermostabilization of the transporters was performed to stabilize the inhibitor-bound, outward-open conformation^12,13^. There was no such stabilization of conformation employed in rGAT1. In LeuT it was possible to obtain an inward-occluded structure by perturbing the intracellular gate and when this was done together with the perturbation of the Na2 site, an inward-open apo-structure was obtained^15,43^. GABA interactions in rGAT1 are observed to close the extracellular gate through cysteine crosslinking studies^44^. A plausible explanation for the inside-open conformation observed in rGAT1 and hGAT1 structures could be due to the unique insertion of an extra amino acid (either S456 or G457) in TM10 that results in the formation of a π-helical segment and accentuates the H-bond network in GAT1 making it stronger than other members of SLC6 family.”**

**In the context of TM10, we have edited this as per the suggestion of Reviewer 1 to indicate the presence of a single amino acid insertion that induces a π-helix formation. Further, we have also compared the extracellular gate H-bond network among SLC6 members and LeuT in a new ED figure 6.**

**We have included an analysis of rGAT1 cytosolic gate residues and observe substitutions of hydrophobic residues that could weaken the hydrophobic interactions at the cytosolic half of the transporter. This is represented as a figure in supplementary, S2b inset.**

**Top of page 11:“The presence of an open cytosolic gate could be facilitated by substitutions of hydrophobic residues observed in rGAT1 in comparison to other biogenic amine transporters like hSERT. For instance, F347 (hSERT) is substituted to a leucine (L306, TM6b) in rGAT1. Similarly cysteine residues are observed in rGAT1 (C57 (TM1a), C102 (TM2)) instead of the hydrophobic residues in hSERT (V92, L137) (Fig. S2b, inset). These substitutions can alter the hydrophobic interactions within the cytosolic gate and likely enhance the propensity of rGAT1 to attain a cytosolic-facing conformation.”**

**We hope that the arguments presented above give a satisfactory rationale to the reviewer’s query about the basis for observing a cytosol-facing GAT1 structure.**

There is also a brief discussion of the effects of extracellular pathway mutations on transport activity and Na+ binding (page 6). Is it possible to relate the behavior of GAT1 in oocytes, for example, with its conformation? Is there a step preceding extracellular Na+ binding that is detectible (by a transient current, for example) that could correspond to a conformational transition from the inward- to outward-facing conformation? Such an observation could help to validate the inward-facing structure shown here and to relate it to the behavior of GAT1 in a cell membrane.

**Response: The following sentences have been added to the text on the lower half of page 6 and the top of page 7 to comment on the mutations at the extracellular vestibule and their effect on the GAT1 currents using oocyte measurements. “R69K, Q291N and D451E have been analyzed by using the two-electrode-voltage clamp methodology. Even though these conservative replacement mutants have impaired transport, they all exhibit capacitative transient currents**^28-30^**, reflecting the transition between empty-inward and empty-outward conformations, preceding the binding of extra-cellular sodium, which stabilizes the outward conformation**^30,31^. **The voltage dependence of transient currents by GAT1_WT_ indicates that under physiological conditions a significant proportion of the transporters is inward-open. In the mutants this voltage dependence is markedly left-shifted relative to those by rGAT1_WT_ indicating a much larger proportion of outward-open transporters, apparently as a result of a perturbed extracellular gate**^28-30^**. Therefore these transporters are “stuck” in an outward-open conformation and cannot cycle through inward-open conformations, resulting in impaired transport.”.**

The lack of Na+ in the Na2 site is consistent with the open cytoplasmic pathway. What was the Na+ concentration in the sample prep? All I can gather is that it was between 50 mM in the antibody prep and 300 mM for the transporter. Is it possible that an increase in [Na+] would fill the Na2 site and close the pathway? Was this ever tested? The possibility should be discussed.

**Response: The concentrations are indicated in the method sections page 16 i. Heterologous expression and purification of GAT1 and the sodium concentration for binding assay is mentioned in page 18 of method dealing with MS binding assays.**

**The concentration of NaCl in the sample was at 300 mM as the protein was complexed by adding a concentrated solution of 9D5 fab. The MS binding studies were done at a high concentration of 1M of NaCl as per previously optimized protocols that along with the membrane environment likely induces an outward –open conformation that would allow inhibitor interactions. This will be a strategy that we hope to explore for future structural studies.**

Could the differences in KM and inhibitor affinity between GAT1WT and GAT1EM be due to a conformational effect of mutation? Does binding of the antibody influence these values? If it did, and this would be easy to measure, it would explain the difference in affinity or KM, but also would present a problem for interpreting this structure as a native one.

**Response: We observe that there are no major structural differences in rGAT1 upon epitope transfer as observed through a comparison of the IL5 residues in rGAT1_EM_ with the unmodified hGAT1 inserted as a figure below reveals a near perfect overlap. The antibody binding does not seem to influence the conformational changes as observed with dDAT where it is observed to bind an outward-open transporter and in rGAT1 binds to an inward-open conformation.**

**While we cannot test the direct effect of Fab on the activity of transporter, the structure represents a functional native transporter given the clear presence of uptake activity and inhibitor interactions observed in our study. We further included an image in the comments (above) comparing the local structures of hGAT1 and rGAT1 around the IL5 region where a bulk of the epitope residue changes were made. Both the molecules show a well-overlapped structure in the region suggesting that the changes to IL5 are well accommodated and do not disrupt the local structure of the transporter.**

Because GABA is a γ-amino acid, unlike α-amino acids that are transported by most members of this family, the coordination of the amino group is likely to differ. For example, in LeuT Ser256, which corresponds to Gly297 in GAT1. It would be useful to compare the interactions between substrate and coordinating residues in this structure with those of LeuT or MhsT.

**Response: We explain this aspect of the reviewer’s comment through new figure panels in ED. Fig8. The following lines have been added to the discussion (top of page 13) to address this comment.**

**“In LeuT, which transports α-amino acids, the substrates interact with the hydroxyl of S256 (TM6), which is substituted to a glycine in GAT1 (G297) thereby compromising this interaction (Extended Data Fig. 8e, f). It is attractive to speculate that the location of the γ-amino group of GABA in the vicinity of TM10 accommodates the distance constraint on GABA in the binding pocket.”**

The authors describe “movement” between this structure and an outward-facing structure. No outward-facing GAT-1 structure is available, and I don’t think the structure of Drosophila DAT with binding site residues changed to those of GAT1 is a good basis for a model. Did the author make a model based on LeuT or other members of the family? There are many places in the manuscript where such movements are implied without a point of reference. Without at least a model of outward-facing GAT1, these statements are hard to accept and should be either explained better or removed. There are some places in the manuscript where the authors describe a sequence of events (for example in the section “Opening of cytosolic gates…”) where they describe a sequence of events that transform the transporter from an unknown conformation to the structure they present. It is important to describe this process as speculation, not as observation. All we have is this structure and a proposed
closed structure and the conversion between the two is not an experimental finding but rather the authors interpretation and should be presented as such and not stated as fact. There are several places in the manuscript where this problem shows up. I am relying on the authors to correct them all. There is a place for speculation, but it needs to be presented as such.

**Response: We built a rGAT1 outward open model using Alphafold2 that in all likelihood represents an outward open conformation given that the AI based prediction will incorporate all the previous information using LeuT, dDAT and hSERT outward open structures. We used this to remake all the figures and indicate angular changes in the figure panels. We do agree with the reviewer that we have to be careful in our interpretation, at multiple points in the discussion we highlight that conformational changes are inferred changes given the 3D model and accurate changes would be inferred when an outward open experimental structure of GAT1 is available in the future.**

**At the beginning of page 7 we have added the following lines, “The likely movements of the extracellular gate from outward-open state to the closed state can best be summarized by comparison of the inward-open structure with the rGAT1 AlphaFold2 model we generated in the outward-open conformation. A structural overlap of the rGAT1 outward-open AlphaFold2 model with the cytosol-facing rGAT1 cryoEM structure predicts the inward movement of TM1b and TM6a by 15° and 17° that allows formation of the close-knit H-bond interactions observed in the extracellular gate of GAT1 (Fig. 2e).**”

**In the middle of page 10: “****From the comparison of the inward-open structure with our AlphaFold2 model, we speculate that solvent access from the cytoplasm to the substrate-binding site is facilitated by the shifts in the unwound regions of TM1 and TM6 followed by the opening of the cytosolic gate through the angular movement of TM1a (Fig. 4a). Because of the transition from outward to inward-open states, the primary binding site position is also lowered toward to cytosolic half, reflected in a 3-4 Å downward displacement in the positions of TM1 and TM6 in addition to the angular movement observed in overlaps of the outward-open model of rGAT1 with the cytosol-facing structure of rGAT1_EM_ (Fig. 4a).”**

**In the discussion (lower part of page 13) we added the following lines, “During a reconstructed GABA reuptake cycle, based on multiple biochemical observations and structural comparisons between rGAT1 model in outward-open conformation and the cryoEM structure in cytosol facing state, two major conformational changes could take place.”**

One observation that seems an interesting possibility for speculation is the interaction between the substrate carboxyl group, Na+, and Tyr140. In LeuT structures, the carboxyl group bridges the corresponding TM3 Tyr108 and Na+ bound at Na1. Here, the GABA carboxyl group is still coordinated with both Tyr104 and Na+, but it is no longer bridging the two, which directly coordinate with each other. However, the Na+ ion is further from Cl- than in structures of other members of this family, suggesting that it has started to move, together with substrate, towards the open cytoplasmic pathway. The distance between Na^+^ and Cl^-^ in the other structures is about 6 Å or less but in this structure, they are 8 Å apart.

**Response: We concur with the reviewer and edited the text as follows at the beginning of page 9.** **“Chloride is suggested to aid in attracting Na^+^ to bind GAT1 and to provide the required anionic charge for GABA translocation^34,35^, but a recent study indicates that chloride interaction with its coordinating glutamine residue allows the formation of an ion pair between TM1 and TM10 which closes the extra-cellular pathway^36^. In the rGAT1 structure, the distance between chloride and sodium 1 is larger than in the other NSS structures at 8 Å versus 5 Å, suggesting that the cation has started to move. In contrast to sodium site 1, there was no evidence of any density at sodium site 2 where the coordination for Na^+^ is disrupted due to conformational changes in TM1, TM5 and TM8 (Fig. 3d, ED Fig. 8b).”**

**We also include the statement of the reviewer concerning Y140 interactions at the end of page 9 as follows, “Despite its importance for substrate recognition, Y140,^39^ while in the vicinity of GABA carboxyl group (4.1 Å) is not engaged in direct interactions with the carboxylate of GABA, which no longer bridges the Y140 with Na^+^ ion. It is however in proximity (3.6 Å) to the modeled Na^+^ ion in the structure (Fig. 3c).”**

Why compare GABA binding site with that of biogenic amine transporters? Aren’t LeuT or other amino acid transporters better models? If you compare amino acids bound in LeuT with 5-HT bound to SERT, the differences are profound.

**Response: We wanted to primarily highlight the shift of the trilobed binding site in biogenic amine transporters to the bean shaped binding site in GATs despite both being SLC6 members. This is primarily the reason for adding the panel. We have now included the binding site comparison with the LeuT as well to panel c and e in the extended data fig. 8.**

Minor points:
Page 3, The rocking bundle hypothesis was an early and simplistic mechanism. It is now quite clear that the bundle (not rocking bundle) helices (1,2,6 and 7) pivot from the cytoplasmic face of the transporter to open and close the extracellular pathway but the cytoplasmic pathway opens by the movement of TM1a, which pivots from the center of the transporter to swing away from the rest of the bundle and open the pathway. In other words, the bundle does not rock to open the cytoplasmic pathway.
The manuscript uses words like above and below to indicate position towards the extracellular or cytoplasmic surface. This needs to be clearly stated to avoid confusion.

**Response: the text as described by the reviewer has been added to the section in the lower part of page 3.**

**“The topology comprises a set of scaffold helices and a bundle consisting of two symmetric but discontinuous helices, TMs1 and 6 that together with TMs 2 and 7 moves relative to the scaffold helices to open and close the extracellular pathway. The cytoplasmic pathway opens by the additional movement of TM1a, which pivots from the center of the transporter to swing away from the rest of the bundle. Thereby a vestibule is opened which allows solvent access towards the cytosol^15,16^”.
We have also included a clear context of extracellular or intracellular whenever the words “above”, “beneath” or “roof” are included in the text.**

**For instance, at the top of page 4. “… the transporter “beneath” (towards the cytosol) the substrate-binding site.”**

**In page 7, para 2, “F294 that forms the “roof” of the binding site facing the extracellular vestibule”.**

Page 3, The ibogaine binding site overlaps with the substrate binding site of SERT. My impression is that the same is true for tiagabine. It is a misstatement that the binding sites are below or beneath the substrate site.

**Response: We have addressed this in two places:**

**Lower part of page 3: Competitive inhibitors are observed to interact with SLC6 members at the substrate-binding site to stabilize the outward-open conformation^13,19,20^ and non-competitive inhibitors have multiple interaction sites either towards the extracellular vestibule or at the cytosolic half of the transporter as observed in citalopram-bound hSERT, ibogaine-bound hSERT and cmpd1 bound hGlyT1^13,14,18^.**

**Middle part of page 11:** **The GABA position in rGAT1 is lower in comparison to the inhibitor bound, outward-open dDAT_GAT_ structures as a consequence of its transition to the inward-open state. The extra bulk of tiagabine bound to hGAT1, that of the bitopterin analogue, cmpd1 bound to hGlyT1 and ibogaine bound hSERT extends to positions that are proximal to the cytoplasm (Fig. 4c,d).**

Page 5, What is the evidence that the first 38 residues of GAT1 are disordered?

**Response: The AlphaFold2 models of both rGAT1 and hGAT1 display a disordered N-terminus (upper part of page 5). Given the general accuracy of prediction by the software for structured regions we stated in the manuscript that this stretch of the N-terminus is disordered. It has also been a general practice for crystallographic and cryoEM studies to delete these disordered stretches of the N-terminus that do not hamper the reuptake activity.**

Page 5, KM is not affinity or apparent affinity. It is a kinetic constant that, only under some special conditions can equal the KD for substrate.

**Response: We have made the appropriate corrections in the text (middle of page 5) and the statement reads as follows, “rGAT1_EM_ is a functional construct displaying ^3^H-GABA uptake activity with a lower *K_M_* for GABA (*K*_M_ of 4.2 μM) and lowered V_max_ in comparison to rGAT1_WT_ that has a *K*_M_ value of 11 μM”.**

Page 7, Instead of referring to the position of bound Na+ as “traditional”, it might be more useful to describe which residues it interacts with in other transporters or the distance from the bound Cl-.

**Response: In response to the reviewer’s comment we have made the following edit to the text (middle of page 8). “However, the sodium site appears displaced from coordinating site between TM1 and TM6 observed in the published NSS structures containing sodium at site 1. As observed in inhibitor-bound outward-open dDAT_GAT_^22^ and other SLC6 members, the Na^+^ coordination at site 1 in rGAT1 would comprise the carboxyl oxygen of the substrate, the side-chain oxygens of N66, S295, N327 and the main chain carbonyl oxygens of A61 and S295.”**

Page 7, Reference 13 is cited for LeuT. It is actually a reference to SERT.”

**Response: We have removed this reference from the location.**

Page 8, The term “linker” for the unwound (or non-helical) region of TM1 or TM6 is confusing. Please use a more descriptive name.

**Response: We have edited the word linker in most places in the manuscript to suggest unwound or non-helical region of TM1 and TM6.**

Page 8, Chloride is required for many transporters in this family. It’s role has been proposed to involve formation of the TM1-TM10 salt bridge, but there is no specific relationship between the charge on Cl- and GABA transport outside of that or the electrostatic interaction with Na+.

**Response: The following text is now added in the top of page 9: “Chloride is suggested to aid in attracting Na^+^ to bind GAT1 and to provide the required anionic charge for GABA translocation^34,35^, but a recent study indicates that chloride interaction with its coordinating glutamine residue allows the formation of an ion pair between TM1 and TM10 which closes the extra-cellular pathway^36^.**

Page 9, There are many LeuT structures where Y108, which corresponds to GAT1 Y140, interacts with the substrate carboxyl group, and it would be surprising if the GABA carboxyl did not coordinate Y140 in GAT1 in the outward-occluded or -open conformations. The statement in the middle of the first paragraph on page 9 should be modified so it doesn’t give the impression that the two do not interact in other states of the complex.

**Response: We introduced a statement at the beginning of page 10 to address this concern. “Interaction of GABA with Y140 could be more pronounced in alternate conformational states of GAT1 like the outward-open or outward-occluded states as GABA is recognized and transported across the bilayer.”**

**We further performed MD simulations (ED Fig. 9) in the manuscript to facilitate this depiction. The simulation data as suggested by reviewer 1 shows the interaction of GABA with Y140 over 100 ns and enhanced flexibility of GABA in the binding pocket in the Y140F substitution.**

Page 9, 2/3 from the top: …50 to 60. TM1a, in… …where TM1a is closer to TM7,…
…N-terminus. TM1a could…position through the transport cycle. The corresponding residue, Y60,

**Response: The text changes have been done.**
Page 10, 3rd line of discussion, …targets is a lengthy and resource…

**Response: Changed as suggested.**
Page 11, middle, …site 2 is proposed to initiate the release of…

**Response: Altered**
Page 11, …that were first observed on the cytoplasmic face…

**Response: The above typographical changes have been made in the revised manuscript.**

**Additional Changes:**

**In addition to the above suggested changes by the reviewers,**

**We have added an RT-PCR data for GAD1 to demonstrate the presence of glutamate decarboxylase transcripts in HEK293 cells.**

**We have further made appropriate edits to the manuscript to improve the flow and lucidity of the manuscript.**

**Method section is suitably modified to reflect all the experimental procedures carried out during revision.**

**Additional references have been added as required in the text.**

**Decision Letter, first revision:**

| **Message:** | 6th Jan 2023  Dear Dr. Penmatsa,  Thank you again for submitting your manuscript "Cryo-EM structure of GABA transporter 1 reveals substrate recognition and transport mechanism". I apologize for the delay in responding, which resulted from the difficulty in obtaining suitable referee reports. Nevertheless, we now have comments (below) from the 2 reviewers who evaluated your paper. In light of those reports, we remain interested in your study and would like to see your response to the comments of the referees, in the form of a revised manuscript.  You will see that while both reviewers are positive about the improvements made in revision, some of their concern remain unresolved. Specifically, reviewer #1 remains unconvinced by GABA density in the cryo-EM reconstruction. We recommend you consider their suggestions regarding further data processing to obtain a homogenous class with a stronger GABA density. Please be sure to address/respond to all concerns of the referees in full in a point-by-point response and highlight all changes in the revised manuscript text file. If you have comments that are intended for editors only, please include those in a separate cover letter.   We are committed to providing a fair and constructive peer-review process. Do not hesitate to contact us if there are specific requests from the reviewers that you believe are technically impossible or unlikely to yield a meaningful outcome.  We expect to see your revised manuscript within 6 weeks. If you cannot send it within this time, please contact us to discuss an extension; we would still consider your revision, provided that no similar work has been accepted for publication at NSMB or published elsewhere.  As you already know, we put great emphasis on ensuring that the methods and statistics reported in our papers are correct and accurate. As such, if there are any changes that should be reported, please submit an updated version of the Reporting Summary along with your revision.  Please follow the links below to download these files:  Reporting Summary: https://www.nature.com/documents/nr-reporting-summary.pdf  Please note that the form is a dynamic ‘smart pdf’ and must therefore be downloaded and completed in Adobe Reader.   When submitting the revised version of your manuscript, please pay close attention to our href="https://www.nature.com/nature-portfolio/editorial-policies/image-integrity">Digital Image Integrity Guidelines.</a> and to the following points below:  -- that unprocessed scans are clearly labelled and match the gels and western blots presented in figures. -- that control panels for gels and western blots are appropriately described as loading on sample processing controls -- all images in the paper are checked for duplication of panels and for splicing of gel lanes.  Finally, please ensure that you retain unprocessed data and metadata files after publication, ideally archiving data in perpetuity, as these may be requested during the peer review and production process or after publication if any issues arise.   If there are additional or modified structures presented in the final revision, please submit the corresponding PDB validation reports.  Please note that all key data shown in the main figures as cropped gels or blots should be presented in uncropped form, with molecular weight markers. These data can be aggregated into a single supplementary figure item. While these data can be displayed in a relatively informal style, they must refer back to the relevant figures. These data should be submitted with the final revision, as source data, prior to acceptance, but you may want to start putting it together at this point.  SOURCE DATA: we urge authors to provide, in tabular form, the data underlying the graphical representations used in figures. This is to further increase transparency in data reporting, as detailed in this editorial (http://www.nature.com/nsmb/journal/v22/n10/full/nsmb.3110.html). Spreadsheets can be submitted in excel format. Only one (1) file per figure is permitted; thus, for multi-paneled figures, the source data for each panel should be clearly labeled in the Excel file; alternately the data can be provided as multiple, clearly labeled sheets in an Excel file. When submitting files, the title field should indicate which figure the source data pertains to. We encourage our authors to provide source data at the revision stage, so that they are part of the peer-review process.  Data availability: this journal strongly supports public availability of data. All data used in accepted papers should be available via a public data repository, or alternatively, as Supplementary Information. If data can only be shared on request, please explain why in your Data Availability Statement, and also in the correspondence with your editor. Please note that for some data types, deposition in a public repository is mandatory - more information on our data deposition policies and available repositories can be found below: https://www.nature.com/nature-research/editorial-policies/reporting-standards#availability-of-data  We require deposition of coordinates (and, in the case of crystal structures, structure factors) into the Protein Data Bank with the designation of immediate release upon publication (HPUB). Electron microscopy-derived density maps and coordinate data must be deposited in EMDB and released upon publication. Deposition and immediate release of NMR chemical shift assignments are highly encouraged. Deposition of deep sequencing and microarray data is mandatory, and the datasets must be released prior to or upon publication. To avoid delays in publication, dataset accession numbers must be supplied with the final accepted manuscript and appropriate release dates must be indicated at the galley proof stage.  While we encourage the use of color in preparing figures, please note that this will incur a charge to partially defray the cost of printing. Information about color charges can be found at http://www.nature.com/nsmb/authors/submit/index.html#costs  Nature Structural & Molecular Biology is committed to improving transparency in authorship. As part of our efforts in this direction, we are now requesting that all authors identified as ‘corresponding author’ on published papers create and link their Open Researcher and Contributor Identifier (ORCID) with their account on the Manuscript Tracking System (MTS), prior to acceptance. This applies to primary research papers only. ORCID helps the scientific community achieve unambiguous attribution of all scholarly contributions. You can create and link your ORCID from the home page of the MTS by clicking on ‘Modify my Springer Nature account’. For more information please visit please visit <a href="http://www.springernature.com/orcid">www.springernature.com/orcid</a>.  Please use the link below to submit your revised manuscript and related files:  [redacted]  <strong>Note:</strong> This URL links to your confidential home page and associated information about manuscripts you may have submitted, or that you are reviewing for us. If you wish to forward this email to co-authors, please delete the link to your homepage.  We look forward to seeing the revised manuscript and thank you for the opportunity to review your work.  Sincerely,  Katarzyna Ciazynska (she/her) Associate Editor Nature Structural & Molecular Biology https://orcid.org/0000-0002-9899-2428   Referee expertise:  Referee #1: cryo-EM, transporters  Referee #2: structural biology, transporters   Reviewers' Comments:  Reviewer #1: Remarks to the Author: Thanks to the authors for providing further details and improving the manuscript.  I do not, however, still agree that the density assigned to GABA is very clear: The GABA fit into the density is not great (Figure 3b). Looking at the map, the density to which a Na ion is assigned is visible at around 9.0 rmsd (sigma level), however at this rmsd the density for GABA is very weak (and not continuous). My concern remains whether this density belongs to GABA. It is Very unlikely that the density for ion is stronger than the density for the bound ligand. Specially that the binding mode is not really stabilized through interactions with surrounding residues (and as stated before, the carboxyl group binding site is conserved from bacteria to human in this family of transporters and the different binding site raises concern).  Have the authors tried to co-purify the transporter with GABA? Are there any other components in expression/purification solutions that can fit into this density?  There seems to be above 1,000,000 particles used for final refinement. If it has not already been tried, I would suggest trying to clean this particle stack further, e.g., by doing ab initio classification with a greater number of classes (e.g, try 3, 5, 7) and cleaning further with heterogenous refinement. There might be a more homogenous class of particles as a subset of these 1,000,000 particles with robust ligand binding that can participate in stronger density. A reason for rather weak density of the ligand could be partial binding, again as the network of interactions does not support this binding. I would assume with a smaller, but "cleaner" particle stack, the authors could reach a similar resolution, if not better.    Reviewer #2: Remarks to the Author: With the exception of one section, which I think needs more explanation, most of my suggestions concerning this revision are minor. I list them here in the order they appear in the manuscript.  Line Comment 74 vestibule or in the 76 interactions in the 91-2 with an antigen-binding fragment (Fab) 93 reveals a closed 97 The observation that detergent-solubilized transporter adopts a cytosol-facing conformation  104 Results 129 construct catalyzing 3H-GABA uptake with 145 This appears to be one paragraph ending at line 231. If so, please break it up for clarity  177 Mutating residues 179 Is transport blocked (or just reduced) by these mutations? It is not clear from the way it is written.  183 This addition referring to the oocyte voltage-clamp studies is important, but it is impossible for a reader to follow the logic without digesting the cited papers first. It needs more explanation. How are the transient currents induced? What is the interpretation of a left- or right-shift in the voltage dependence, etc. I don’t know whether it’s feasible to include the explanation required for a naïve reader to be able to follow the arguments. If the authors can do that, great. If not, just summarize the conclusions of the voltage-clamp studies.  244 There needs to be a distinction between the ion (Na+) and the site (Na1 or Na2). It might be helpful to define the difference. In any case, I think it will be less confusing if the location of an ion in the structure is not described as a site unless the ion is bound at one of the established Na+ sites. For example, “the location of Na+ was displaced from the Na1 site by 3Å”.  265 There is no requirement for charges moved across the membrane to be balanced. GAT1 transports 1 net positive charge inward in each cycle, GlyT2 transports 2 and SERT transports none. I suggest changing this line to “…GAT1 and to be symported with GABA”  268 Reference 29 could also be cited at the end of this sentence. 279 proposed to be required for the transition 389 both dDAT and SERT X-ray structures 391 employed in the cryo-EM structures of SERT (refs 18 and Yang et al. 2021) or rGAT1. 395 gate through cysteine modification (also could cite refs 29 and 36 here) |
| --- | --- |

**Author Rebuttal, first revision:**

Reviewer #1:
Remarks to the Author:
Thanks to the authors for providing further details and improving the manuscript.

I do not, however, still agree that the density assigned to GABA is very clear: The GABA fit into the density is not great (Figure 3b). Looking at the map, the density to which a Na ion is assigned is visible at around 9.0 rmsd (sigma level), however at this rmsd the density for GABA is very weak (and not continuous). My concern remains whether this density belongs to GABA. It is Very unlikely that the density for ion is stronger than the density for the bound ligand. Specially that the binding mode is not really stabilized through interactions with surrounding residues (and as stated before, the carboxyl group binding site is conserved from bacteria to human in this family of transporters and the different binding site raises concern).

**Response: We appreciate the concern of the reviewer and have extensively performed suggested refinement steps (described later). The newly refined map for GABA fits into the linear density with interactions at subsite A that are conventionally associated with the GABA substrate including Y140, T400 and the ion density. The GABA molecule due to its unbranched alkane carbon chain containing backbone can be highly flexible that causes a likely weakening of the density near the linear methyl carbon atoms. The carboxylate that is more electron dense retains a density similar to the ion density upto a high rmsd. As suggested by the reviewer 2 we also indicate in the manuscript that modeled Na^+^ is displaced from site 1 which is not occupied in the structure.**

**The newer position of the GABA molecule and the difference in the densities between the earlier and current coordinates is described in a figure below. In the newer map the GABA has a marginally improved fit with a cc of 0.72 in comparison to the old coordinates that had a cc of 0.68, shown below at the same view and density contour levels (5σ). The carboxylate could also be modeled completely inside the density and is within interaction distance of the Y140 hydroxyl. In a similar context, a recent study on hSERT has a serotonin molecule bound in the cytosol-facing state with the amine group interacting at 3.6 angstroms distance from D98 which is the neurotransmitter recognition site in subsite A of hSERT. (Yang and Gouaux, 2021, Sci. Adv. doi: 10.1126/sciadv.abl3857).**

Have the authors tried to co-purify the transporter with GABA? Are there any other components in expression/purification solutions that can fit into this density?
**Response: We have clearly mentioned in the manuscript that we did not add GABA after purification of rGAT1 in the following lines, “The observed density fits well for a molecule of GABA (Fig. 3a) although GABA was not added during the purification process. We suspected that GABA could be produced and released from HEK293 cells that we used for expression of rGAT1”. We therefore performed extensive characterization of HEK293 cell lysates to establish that GABA is indeed produced in large quantities within the cells that can interact with the overexpressed transporter during extraction from cell membranes. We have employed highly sensitive ESI-MS/MS experiments and quantitative real time pCR to demonstrate the presence of GABA and the GABA producing glutamate decarboxylase 1 enzyme within HEK293 cells, respectively. It is rather difficult to speculate which other purification components may fit into the density as the buffer components and the additives used are substantially larger than the density observed and other cellular components that can bind GAT1 would be difficult to predict particularly given that GAT1 is a highly specific transporter for GABA which is a γ-aminoacid.**

There seems to be above 1,000,000 particles used for final refinement. If it has not already been tried, I would suggest trying to clean this particle stack further, e.g., by doing ab initio classification with a greater number of classes (e.g, try 3, 5, 7) and cleaning further with heterogenous refinement. There might be a more homogenous class of particles as a subset of these 1,000,000 particles with robust ligand binding that can participate in stronger density. A reason for rather weak density of the ligand could be partial binding, again as the network of interactions does not support this binding. I would assume with a smaller, but "cleaner" particle stack, the authors could reach a similar resolution, if not better.

**Response: The reviewer is right in pointing out the large size of the particle set used for the final refinement. As per the suggestion, we have extensively tried ab-initio and heterogenous refinement in multiple steps and identified a subset of particles amounting to ~870,000 particles with a highly homogenous particle distribution that could be used for final reconstruction (figure below). This particle set yielded a marginal improvement in resolution and also the density of the substrate molecule allowed for a cleaner positioning of GABA. Further dividing the particle set led to compromised resolution of the reconstruction down to 3.5 Å that was not helpful. Moreover, unlike the previously refined structure, we could model the GABA carboxylate functional group at a distance of 3.6 angstroms from Y140 hydroxyl group and the GABA interacts closer to other moeities like S396 and T400 in the binding pocket. This is also reinforced in the additional round of MD simulations reperformed with the newer coordinates in the revised manuscript where GABA forms close H-bond networks near the subsite A in proximity with the Y140, T400 and the Na^+^ ion and the GABA molecule drifts away when Y140 is substituted. We thank the reviewer for this suggestion and have accordingly modified the manuscript to highlight these interactions and we hope that the revised coordinates around the substrate binding pocket are acceptable to the reviewer. We have also correspondingly updated the pdb files in the database.**

**Reviewer #2:**
Remarks to the Author:
With the exception of one section, which I think needs more explanation, most of my suggestions concerning this revision are minor. I list them here in the order they appear in the manuscript.

**Response: We thank the reviewer for the suggestions and edits and we have incorporated them in enitirety in the revised manuscript.**

Line Comment
74 vestibule or in the
76 interactions in the
91-2 with an antigen-binding fragment (Fab)
93 reveals a closed
97 The observation that detergent-solubilized transporter adopts a cytosol-facing conformation

104 Results
129 construct catalyzing 3H-GABA uptake with
145 This appears to be one paragraph ending at line 231. If so, please break it up for clarity.

**Response: We have clarified all the above points and included the edits in the revised manuscript.**

177 Mutating residues
179 Is transport blocked (or just reduced) by these mutations? It is not clear from the way it is written.

**Response: We have clarified this statement and include the statement “blocked” instead of compromised in the manuscript.**

183 This addition referring to the oocyte voltage-clamp studies is important, but it is impossible for a reader to follow the logic without digesting the cited papers first. It needs more explanation. How are the transient currents induced? What is the interpretation of a left- or right-shift in the voltage dependence, etc. I don’t know whether it’s feasible to include the explanation required for a naïve reader to be able to follow the arguments. If the authors can do that, great. If not, just summarize the conclusions of the voltage-clamp studies.

**Response: We summarized the literature from the voltage clamp studies as follows, “Mutating residues vital for gating at the extracellular side through substitutions like R69K lead to blocked GABA uptake and most substitutions result in the loss of uptake activity in GAT1 suggesting their importance in ion-coupled GABA transport^28-30^. R69K, Q291N and D451E have been analyzed by using the two-electrode-voltage clamp methodology. Even though these conservative replacement mutants have impaired transport, they all exhibit sodium-dependent capacitative transient currents^28-30^. These currents are observed in the absence of GABA upon jumping the membrane potential from a holding potential to a range of membrane potentials. They reflect the transition between empty-inward and empty-outward conformations, preceding the binding of extra-cellular sodium, which stabilizes the outward conformation^30,31^. The voltage dependence of these transient currents indicates that in the mutants much higher potentials (inside positive) are required to “push-off” the sodium than in GAT1_WT_, In other words the external gate mutants have an increased apparent affinity for extracellular sodium apparently as a result of a perturbed extracellular gate^28-30^. Therefore, these transporters are “stuck” in an outward-open conformation and cannot cycle through inward-open conformations, resulting in blocked transport.”**

244 There needs to be a distinction between the ion (Na+) and the site (Na1 or Na2). It might be helpful to define the difference. In any case, I think it will be less confusing if the location of an ion in the structure is not described as a site unless the ion is bound at one of the established Na+ sites. For example, “the location of Na+ was displaced from the Na1 site by 3Å”.

**Response: We agree with the reviewer and make this correction through the manuscript and do not refer to the Na+ density as site 1.**

265 There is no requirement for charges moved across the membrane to be balanced. GAT1 transports 1 net positive charge inward in each cycle, GlyT2 transports 2 and SERT transports none. I suggest changing this line to “…GAT1 and to be symported with GABA”
**Response: The above line has been edited.**

268 Reference 29 could also be cited at the end of this sentence.

**Response: Cited as per suggestion. Some of the references would be shifted as we had to restrict our citations to 60 as per the journal policy.**
279 proposed to be required for the transition
389 both dDAT and SERT X-ray structures
391 employed in the cryo-EM structures of SERT (refs 18 and Yang et al. 2021) or rGAT1.
395 gate through cysteine modification (also could cite refs 29 and 36 here)

**Response: The above modifications have been inc**

**Decision Letter, second revision:**

| **Message:** | Our ref: NSMB-A46818B  7th Mar 2023  Dear Dr. Penmatsa,  Thank you for submitting your revised manuscript "Cryo-EM structure of GABA transporter 1 reveals substrate recognition and transport mechanism" (NSMB-A46818B). It has now been seen by the original referee and their comments are below. The reviewers find that the paper has improved in revision, and therefore we'll be happy in principle to publish it in Nature Structural & Molecular Biology, pending minor revisions to satisfy the referees' final requests and to comply with our editorial and formatting guidelines.  We are now performing detailed checks on your paper and will send you a checklist detailing our editorial and formatting requirements in about a week. Please do not upload the final materials and make any revisions until you receive this additional information from us.   Thank you again for your interest in Nature Structural & Molecular Biology Please do not hesitate to contact me if you have any questions.  Sincerely,  Katarzyna Ciazynska (she/her) Associate Editor Nature Structural & Molecular Biology https://orcid.org/0000-0002-9899-2428   Reviewer #1 (Remarks to the Author):  Thanks to the authors' efforts, all of my comments have been addressed with only one exception. I am still concerned about locating a Na ion in the respective density (which is too strong for an ion, at 3.1 Å cry EM map). I have attached a screen shot (rmsd: 5.0) with other possibilities. Where Na is located at the moment, is where one would expect the carboxyl group would bind (compare it with e.g. LeuT, MhsT bound to amino acids). If the authors can exclude any sort of carboxyl group binding at this location, such as partial occupancy of GABA, or acetate (if used in any buffers), I have no other comments. But I will appreciate one last effort from the authors, to rule that out. |
| --- | --- |

**Author Rebuttal, second revision:**

**Response to reviewer**

Thanks to the authors' efforts, all of my comments have been addressed with only one exception. I am still concerned about locating a Na ion in the respective density (which is too strong for an ion, at 3.1 Å cry EM map). I have attached a screen shot (rmsd: 5.0) with other possibilities. Where Na is located at the moment, is where one would expect the carboxyl group would bind (compare it with e.g. LeuT, MhsT bound to amino acids). If the authors can exclude any sort of carboxyl group binding at this location, such as partial occupancy of GABA, or acetate (if used in any buffers), I have no other comments. But I will appreciate one last effort from the authors, to rule that out.

Response: We thank the reviewer and we attempt to answer this query with the following analyses. As suggested, we have extensively explored other possibilities of different compounds and potential substrates to fit in the density into which we modelled Na+ in close proximity to GABA but away from Na Site 1. We tried positioning compounds like acetate, Tris, L-alanine, β-alanine (substrate), betaine (substrate) and GABA (substrate) at this position and evaluate the fits of these respective molecule and correlate the real space correlation coefficient as a measure of the individual fits. It is fairly obvious from the figure below that while the carboxylate moiety fits into this density, the rest of the molecule does not fit or has severe clashes with the surrounding residues. While acetate has a decent fit into this density as pointed out by the reviewer, we have not used any acetate in our buffer components. Na^+^ on the other hand is present to a level of 300mM in the solution which prompted us to model Na^+^ in this position which also has a higher CC value compared to all other tested compounds. We therefore rule out using this analyses that any other substrate or its equivalent could fit into this density within the structure. We mention this in the text in the following lines in the “**Since this density appeared strong for a single Na^+^ ion multiple small molecules were tested at this density for potential interactions (Fig. S2). Among the tested compounds only the Na^+^ ion, present at 300 mM in the buffer, fit with highest correlation coefficient (0.6).” The following figure has been included as supplementary figure S2.**

Figure 1. Real space refined positions of Na^+^, L-alanine, β-alanine, GABA, Tris, Acetate, betaine positioned into the density next to the bound GABA molecule.

Figure 2. Correlation coefficient differences of the fits of GABA at the density where it is clearly modelled vs at the additional density into which it was fit to analyse quality.

**Final Decision Letter:**

| **Message:** | 4th May 2023  Dear Dr. Penmatsa,  We are now happy to accept your revised paper "Cryo-EM structure of GABA transporter 1 reveals substrate recognition and transport mechanism" for publication as an Article in Nature Structural & Molecular Biology.  Acceptance is conditional on the manuscript's not being published elsewhere and on there being no announcement of this work to the newspapers, magazines, radio or television until the publication date in Nature Structural & Molecular Biology.  Over the next few weeks, your paper will be copyedited to ensure that it conforms to Nature Structural & Molecular Biology style. Once your paper is typeset, you will receive an email with a link to choose the appropriate publishing options for your paper and our Author Services team will be in touch regarding any additional information that may be required.  After the grant of rights is completed, you will receive a link to your electronic proof via email with a request to make any corrections within 48 hours. If, when you receive your proof, you cannot meet this deadline, please inform us at rjsproduction@springernature.com immediately.  You will not receive your proofs until the publishing agreement has been received through our system.  Due to the importance of these deadlines, we ask that you please let us know now whether you will be difficult to contact over the next month. If this is the case, we ask you provide us with the contact information (email, phone and fax) of someone who will be able to check the proofs on your behalf, and who will be available to address any last-minute problems.  To assist our authors in disseminating their research to the broader community, our SharedIt initiative provides all co-authors with the ability to generate a unique shareable link that will allow anyone (with or without a subscription) to read the published article. Recipients of the link with a subscription will also be able to download and print the PDF.  As soon as your article is published, you can generate your shareable link by entering the DOI of your article here: <a href="http://authors.springernature.com/share">http://authors.springernature.com/share<a>. Corresponding authors will also receive an automated email with the shareable link  Note the policy of the journal on data deposition: http://www.nature.com/authors/policies/availability.html.  Your paper will be published online soon after we receive proof corrections and will appear in print in the next available issue. You can find out your date of online publication by contacting the production team shortly after sending your proof corrections. Content is published online weekly on Mondays and Thursdays, and the embargo is set at 16:00 London time (GMT)/11:00 am US Eastern time (EST) on the day of publication. Now is the time to inform your Public Relations or Press Office about your paper, as they might be interested in promoting its publication. This will allow them time to prepare an accurate and satisfactory press release. Include your manuscript tracking number (NSMB-A46818C) and our journal name, which they will need when they contact our press office.  About one week before your paper is published online, we shall be distributing a press release to news organizations worldwide, which may very well include details of your work. We are happy for your institution or funding agency to prepare its own press release, but it must mention the embargo date and Nature Structural & Molecular Biology. If you or your Press Office have any enquiries in the meantime, please contact press@nature.com.  You can now use a single sign-on for all your accounts, view the status of all your manuscript submissions and reviews, access usage statistics for your published articles and download a record of your refereeing activity for the Nature journals.  If you have not already done so, we strongly recommend that you upload the step-by-step protocols used in this manuscript to the Protocol Exchange. Protocol Exchange is an open online resource that allows researchers to share their detailed experimental know-how. All uploaded protocols are made freely available, assigned DOIs for ease of citation and fully searchable through nature.com. Protocols can be linked to any publications in which they are used and will be linked to from your article. You can also establish a dedicated page to collect all your lab Protocols. By uploading your Protocols to Protocol Exchange, you are enabling researchers to more readily reproduce or adapt the methodology you use, as well as increasing the visibility of your protocols and papers. Upload your Protocols at www.nature.com/protocolexchange/. Further information can be found at www.nature.com/protocolexchange/about.  An online order form for reprints of your paper is available at <a href="https://www.nature.com/reprints/author-reprints.html">https://www.nature.com/reprints/author-reprints.html</a>. Please let your coauthors and your institutions' public affairs office know that they are also welcome to order reprints by this method.  Please note that <i>Nature Structural & Molecular Biology</i> is a Transformative Journal (TJ). Authors may publish their research with us through the traditional subscription access route or make their paper immediately open access through payment of an article-processing charge (APC). Authors will not be required to make a final decision about access to their article until it has been accepted. <a href="https://www.springernature.com/gp/open-research/transformative-journals"> Find out more about Transformative Journals</a>  **Authors may need to take specific actions to achieve <a href="https://www.springernature.com/gp/open-research/funding/policy-compliance-faqs"> compliance</a> with funder and institutional open access mandates.** If your research is supported by a funder that requires immediate open access (e.g. according to <a href="https://www.springernature.com/gp/open-research/plan-s-compliance">Plan S principles</a>) then you should select the gold OA route, and we will direct you to the compliant route where possible. For authors selecting the subscription publication route, the journal’s standard licensing terms will need to be accepted, including <a href="https://www.springernature.com/gp/open-research/policies/journal-policies">self-archiving policies</a>. Those licensing terms will supersede any other terms that the author or any third party may assert apply to any version of the manuscript.   In approximately 10 business days you will receive an email with a link to choose the appropriate publishing options for your paper and our Author Services team will be in touch regarding any additional information that may be required.  You will not receive your proofs until the publishing agreement has been received through our system.  If you have any questions about our publishing options, costs, Open Access requirements, or our legal forms, please contact ASJournals@springernature.com  Sincerely,   Katarzyna Ciazynska (she/her) Associate Editor Nature Structural & Molecular Biology https://orcid.org/0000-0002-9899-2428   Click here if you would like to recommend Nature Structural & Molecular Biology to your librarian: http://www.nature.com/subscriptions/recommend.html#forms |
| --- | --- |
